# Dynamic neuronal ensembles encode burst-suppression revealed by cortex-wide optical-electrical interfaces

**DOI:** 10.1038/s41467-026-72454-0

**Published:** 2026-04-29

**Authors:** Guihua Xiao, Mo Yang, Lingbo Li, Xintong Yao, Jingyu Xie, Xinyue Wang, Jinyu Zang, Yan Zhao, Tianyi Fang, Shuying Wu, Wandi Qi, Shipeng Lin, Wenxi Sun, Ting Lei, Bo Hong, Jiamin Wu, Qionghai Dai, Xiaochuan Dai

**Affiliations:** 1 Beijing National Research Center for Information Science and Technology, Tsinghua University, Beijing, China; 2 Department of Automation, Tsinghua University, Beijing, China; 3 Institute for Brain and Cognitive Sciences, Tsinghua University, Beijing, China; 4 School of Biomedical Engineering, Tsinghua University, Beijing, China; 5 Key Laboratory of Polymer Chemistry and Physics of Ministry of Education, School of Materials Science and Engineering, Peking University, Beijing, China; 6 IDG/McGovern Institute for Brain Research, Tsinghua University, Beijing, China

**Keywords:** Computational neuroscience, Cognitive neuroscience, Extracellular recording

## Abstract

Burst suppression is widely observed across cortical regions during reversible or pathological unconsciousness, yet its neuronal organization remains incompletely understood. Here we present an integrated Cortex-wide Optical-electrical Dual-modal Explorer (CODE) system to examine neuronal dynamics during burst suppression under isoflurane anesthesia in the mouse. We identified distinct cortex-wide neuronal ensembles that alternately associate with burst or suppression events, exhibiting dynamic neuronal recruitment and reactivation during anesthesia. Burst events were marked by highly synchronized neuronal activity early in the burst phase with high functional connectivity, whereas suppression events displayed more asynchronous, temporally distributed activity with reduced connectivity. Transitions between these states involved sequential, directionally organized propagation across cortical regions. ECoG bursts propagated from bilateral sensory cortices to motor areas within tens of milliseconds with increasing synchrony with calcium activity. Furthermore, we established a robust metric linking ECoG and calcium signals, revealing state-dependent interpretability. These findings reveal the single-neuron-to-population architecture of burst suppression and illustrate how integrated optical–electrical measurements enable high-resolution, large-scale interrogation of cortical dynamics.

## Abstract (web summary)

To directly examine how burst-suppression emerges across large neuronal populations, here authors present a Cortex-wide Optical-electrical Dual-modal Explorer (CODE) system. This integrated optical–electrical recording system reveals how large-scale neuronal ensembles organize burst and suppression activity across the mouse cortex during anesthesia, uncovering coordinated dynamics underlying cortical state transitions.

## Introduction

Burst-suppression is a defining electrophysiological signature of profoundly altered brain states^1,2^, commonly observed during general anesthesia, coma, hypothermia, sleep, and early infantile epileptic encephalopathy.^1–3^ Characterized by alternating periods of high-amplitude cortical bursts and quiescent suppressions, this pattern is strongly correlated with the depth of unconsciousness^4,5^. Despite extensive clinical observations and physiological significance, the underlying mechanisms governing burst-suppression transitions—particularly at the single-neuron level—remain poorly understood. Two primary hypotheses have been proposed: the hypersensitivity hypothesis^6^, which posits that cortical neurons exhibit heightened excitability during bursts; and the metabolic hypothesis^7,8^, suggesting that suppression arises from transient reductions in neuronal energy availability. However, these models have largely assumed spatially homogeneous dynamics across the cortex and consistent involvement of fixed neuronal populations. These assumptions are largely derived from macro-scale electrophysiological recordings or optical measurements limited to small neuronal subsets, leaving the cortex-wide cellular organization of burst-suppression insufficiently explored. Therefore, capturing large-population neuronal dynamics across broader cortical regions with sufficient spatiotemporal resolution is essential for unraveling the mechanisms of burst-suppression transitions. This highlights the need for integrated measurement strategies that span simultaneously multiple spatial and temporal scales.

Electroencephalogram (EEG) and ECoG recordings have traditionally portrayed burst-suppression as a globally synchronized process^9–12^. However, recent observations of structured spatiotemporal activity patterns, including traveling waves across cortical areas^13^, suggest that burst-suppression may involve organized mesoscale dynamics rather than uniform global transitions. In recent years, there have been increasing efforts to integrate optical imaging with surface electrophysiology using transparent electrodes based on graphene^14–17^, metal nanoparticles^18,19^, carbon nanotubes^20^, hydrogel^21,22^, and PEDOT-based systems^23–25^. These studies have enabled multimodal measurements and have been optimized for specific experimental goals, such as pairing two-photon cellular-resolution calcium imaging with transparent ECoG arrays^14,20,26^ or wide-field monitoring with limited cellular specificity^27^. In addition, several studies have explored the inference of calcium or spiking activity directly from surface electrophysiology, revealing both the potential and limitations of such cross-modal prediction approaches^28–30^. Together, these efforts motivate the application of integrated multimodal strategies to directly examine how burst-suppression emerges across large neuronal populations.

Recent advances in mesoscale calcium imaging combined with genetically encoded indicators have extended the FOV for observing cortical population activity^31^. Our previously developed real-time, ultra-large-scale, high-resolution (RUSH) system^32^ enables single-cell calcium imaging across a 10 × 12 mm² cortical area at a 5.1 gigapixels per second. While such optical measurements provide extensive spatial coverage, electrophysiological recordings remain essential for resolving the fast temporal dynamics that define burst-suppression^33–35^. In this study, we developed the Cortex-wide Optical-electrical Dual-modal Explorer (CODE), which integrates a transparent 32-channel ECoG array with the RUSH imaging platform. CODE simultaneously delivers single-cell spatial resolution, large FOV over 10 × 12 mm², sub-millisecond temporal resolution (25 μs), and long-term recording stability of at least 7 weeks. By replacing the mouse skull with a transparent ECoG window, CODE enables concurrent calcium imaging of over 10^4^ neurons and high-speed electrophysiological recordings across the dorsal cortex.

Using this integrated multimodal measurement strategy, we investigated the neuronal organization and spatiotemporal dynamics of anesthesia-induced burst-suppression at the cortex-wide scale. With CODE, we identified and tracked two functionally distinct neuronal ensembles comprising over 11,000 burst-related and 7000 suppression-related neurons. These ensembles exhibit spatially distributed yet temporally structured participation, with evolving recruitment and reactivation across individual burst and suppression events. Such dynamics challenge the prevailing notion of spatial homogeneity during burst-suppression. Importantly, burst phases are marked by tightly synchronized neuronal activity and elevated functional connectivity, while suppression phases display asynchronous, spatially diffuse activation with weakened coupling. Leveraging CODE’s synchronous optical-electrical recording, we further uncovered a directional propagation pattern of neuronal activation, progressing from bilateral sensory to medial motor regions, accompanied by a progressive increase in calcium–ECoG synchrony. To quantitatively bridge these two modalities, we established a robust predictive model that infers calcium signals from ECoG features. The resulting cross-modal interpretability was strongly state-dependent—highest during burst, lower in suppression, and minimal during awake states. Together, our findings uncover the mesoscale architecture of burst-suppression transitions and shed light on the neuronal mechanisms underlying loss and recovery of consciousness. Beyond anesthesia, CODE demonstrates its potential for probing cortex-wide spatiotemporal dynamics across diverse brain states and neurological disorders.

## Results

### Simultaneous cortex-wide ECoG and calcium imaging with CODE system

**Fig. 1:**
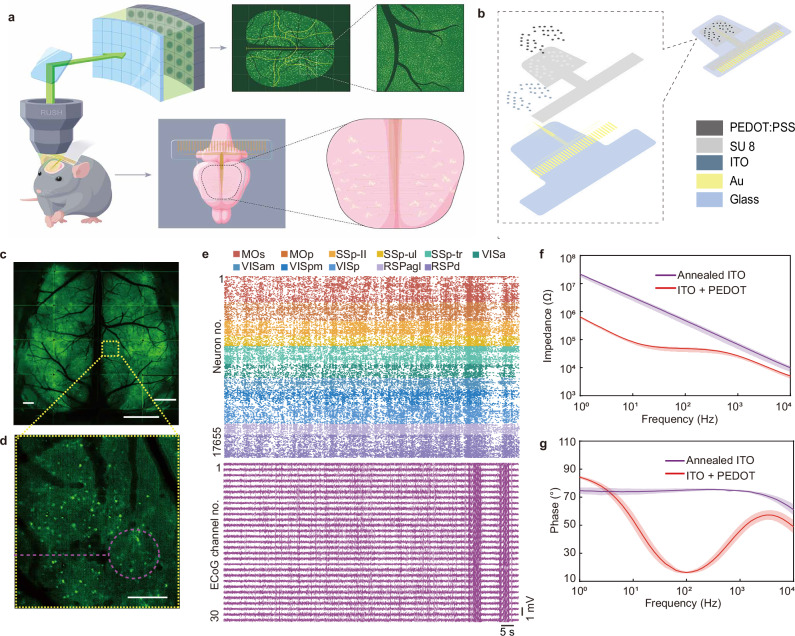
CODE system for simultaneous cortex-wide ECoG and large-scale calcium imaging. **a** Schematic diagram of the CODE system, which integrates the RUSH imaging platform (top) for subcellular resolution calcium imaging with a custom transparent 32-channel ECoG array (bottom) for high-speed electrophysiological recordings. The electrodes are distributed across multiple cortical regions of the mouse brain. **b** Fabrication process of the transparent ECoG array, consisting of five functional layers. **c** Wide-field calcium imaging of the mouse cortex via CODE, showing clear visualization of individual neurons. Scale bar, 2 mm. **d** Magnified view of the region outlined in (**c**), with the purple dashed circle indicating an electrode site on the transparent ECoG array. Scale bar, 200 μm. **e** Representative traces of simultaneously recorded calcium fluorescence (top) involving MOs, MOp, SSp-ll, SSp-ul, VISa, VISam, VISpm, VISp, RSPagl, RSPd regions and electrophysiological signals (bottom) from the 32-channel ECoG array, illustrating temporal synchronization between optical and electrical modalities. **f**–**g** Electrochemical impedance spectroscopy (EIS) characterization of ITO and PEDOT:PSS-modified electrodes, showing the impedance magnitude (**f**) and phase (**g**) at 1 kHz. Shaded areas represent mean ± SEM. Source data are provided as a Source Data file.

To simultaneously capture neural activity across the entire cortex with high temporal and spatial resolution, we developed the CODE system as illustrated in Fig. 1a. Built upon our previously established RUSH imaging platform^32^ and integrated a custom-designed transparent ECoG array, CODE allows cortex-wide, subcellular resolution calcium imaging while concurrently capturing high-speed local field potentials. This dual-modality configuration allows examination of the spatiotemporal relationship between burst-suppression dynamics and single-neuron activity.

In this configuration, a transparent 32-channel ECoG array was placed over the dorsal cortex to permit cortex-wide local field potential recording while maintaining optical access. This array spans 28 cortical regions and is fabricated on a laser-cut glass substrate shaped to match the mouse skull (Fig. 1b). It consists of a glass substrate, gold interconnects, indium tin oxide (ITO) electrode sites, an SU-8 encapsulation layer, and a PEDOT:PSS modification layer (Fig. 1b). Narrow gold interconnects (as small as 2 μm) minimize visual interference, while ITO provides optical transparency and electrical conductivity. PEDOT:PSS coating reduces electrode impedance without substantially affecting optical clarity. This architecture supports stable optical access together with multi-site electrophysiological recordings over extended experiments (see Methods, Supplementary Fig. 1a, b). Optical clarity was confirmed after implantation, with cortical vasculature remaining clearly visible (Supplementary Fig. 1c, d).

In Rasgrf2-2A-dCre/Ai148D transgenic mice expressing GCaMP6f in layer 2/3 pyramidal neurons^36^, individual cells directly beneath or adjacent to electrode sites are both clearly resolved (Fig. 1c, d). Individual neurons were registered to the Allen Mouse Brain Common Coordinate Framework using retinotopic mapping, enabling standardized cortical region alignment. Calcium signals from approximately 18,000 neurons (Fig. 1e, top) were extracted and denoised^37,38^, and temporally aligned with ECoG signals from 28 cortical regions (Fig. 1e, bottom). To accommodate the high data throughput (~ 2 TB per 10 min), a customized processing pipeline was used for neuron detection, background rejection, view stitching, and artifact removal from vascular motion^32^.

Electrode performance was characterized via electrochemical impedance spectroscopy. Bare ITO electrodes exhibit an average impedance of 67.7 ± 16.7 kΩ at 1 kHz, which is significantly reduced to 26.8 ± 2.5 kΩ after PEDOT:PSS coating (Fig. 1f, g), a range suitable for reliable cortical surface electrophysiological recording^39^. Cyclic voltammetry revealed enhanced cathodic charge storage capacity^40^, as indicated by increased shaded area under the curve (Supplementary Fig. 1e), supporting the potential for future stimulation applications. Together, the CODE system provides concurrent cellular-resolution calcium imaging and cortex-wide electrophysiological recordings, establishing the experimental basis for subsequent burst-suppression analyses.

### Characterization of CODE system performance

**Fig. 2:**
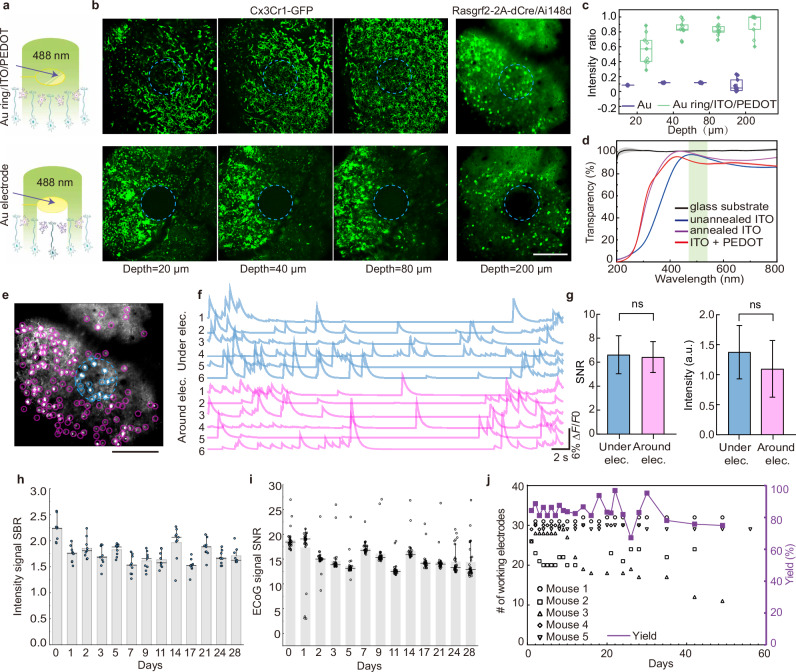
System characterization of the transparent optical-electrical neural interface in chronic recording. **a** Schematic diagrams highlighting the light pathway through traditional opaque gold electrodes (top) and the transparent ECoG electrode site (bottom). The transparent ECoG electrode minimizes light obstruction, enhancing imaging fidelity. **b** In vivo imaging conducted with a two-photon microscope on Cx3Cr1-GFP and Rasgrf2-2A-dCre/Ai148D transgenic mice at various depths through an opaque gold electrode (top) and transparent ECoG electrode (bottom). The electrode site is marked with red dashed circles. Scale bar: 200 μm. Representative images from a single imaging session are shown. Data were obtained from four mice with different genotypes, including experimental (Cx3Cr1-GFP and Rasgrf2-2A-dCre/Ai148D) and control animals. **c** Comparison of intensity contrast between opaque gold electrodes and transparent ECoG electrodes at various imaging depths. Box plots show the median (center line), the interquartile range (box), and whiskers extending to 1.5 × the interquartile range from the box limits. Scattered points represent individual measurements (*n* = 9 per depth for 20, 40, and 80 μm; *n* = 15 for 200 μm). The transparent electrodes exhibit superior transparency and minimal signal attenuation across all imaging depths. **d** Transmittance analysis of bare glass substrate, unannealed ITO (50 nm), annealed ITO, and ITO/PEDOT electrodes. The 485–515 nm wavelength range, which is relevant to the excitation and emission wavelengths in calcium imaging, is highlighted in green. **e** Single-neuron footprints identified from calcium imaging, with locations of highly active neurons indicated by blue (under electrode) and purple circles (around electrode). Scale bar: 200 μm. **f** Fluorescence traces of neurons located directly beneath (top) and around (bottom) the recording electrode, demonstrating clear temporal profiles with no significant interference from the electrode. **g** Comparative analysis of signal-to-noise ratio (SNR) and fluorescence intensity of calcium signals under and surrounding the transparent electrode (mean ± SEM), confirming consistent signal quality in both regions. Around electrode: *n* = 142 neurons for SNR and *n* = 141 neurons for intensity; under electrode: *n* = 13 neurons for SNR and *n* = 12 neurons for intensity. Neurons were recorded from a single mouse during one imaging session. Statistical significance was determined using an unpaired t-test with two-sided Welch’s correction (*p* = 0.68 for SNR, *p* = 0.0577 for intensity). **h** Signal-to-background ratio (SBR) of fluorescence signals from Cx3Cr1-GFP mice over time (mean ± SEM), showing stable performance and minimal signal degradation over extended recording periods. Data were collected from *n* = 9 regions of interest (ROIs) in a single mouse. **i** Signal-to-noise ratio (SNR) evolution of electrophysiological signals recorded from 32 sites in a single implanted ECoG array over time (mean ± SEM), demonstrating high-quality chronic electrophysiological recordings. Data were collected from *n* = 32 recording channels in a single mouse. **j** The effective electrode number in 5 mice, indicating long-term electrode functionality and stability. Statistical comparisons between groups were performed using a two-sided unpaired *t* test, and all tests were two-sided. ns: not significant. Error bars: SEM. Source data are provided as a Source Data file.

For high-quality, large-scale, dual-modal brain recording, the ECoG interface requires sufficient optical transparency, particularly at the electrode sites. In contrast to conventional opaque metal electrodes, which substantially attenuate light transmission, we validated electrode transparency across multiple imaging depths (see “Methods”, Fig. 2b, c and Supplementary Video 1), using two-photon synthetic aperture microscopy (2pSAM)^41^. Clear microglial structures and distinct GCaMP6f-labeled neurons could be visualized both beneath and adjacent to the electrodes, whereas imaging through conventional gold electrodes markedly attenuated fluorescence signals. Quantitative analysis showed higher fluorescence intensity across all imaging depths (Fig. 2c). Spectral transmittance measurement further showed that ITO achieves ~ 97% transmission in the 485–515 nm range—corresponding to the excitation and emission spectra of both GFP and GCaMP6f—while PEDOT:PSS coating modestly reduced transmittance to ~ 90% at electrode sites (Fig. 2d). This level of transparency supports accurate fluorescence detection even directly beneath electrode areas.

To further assess imaging fidelity at the single-cell level, we extracted calcium signals from neurons located directly beneath and adjacent to electrode sites (Fig. 2e–g). Morphological identification confirmed clear visualization of individual somata under the electrodes (Fig. 2e). Sample calcium traces from these neurons exhibited consistent temporal profiles (Fig. 2f), and quantitative analyses showed no significant differences in signal-to-noise ratios (SNR) or calcium event amplitude between neurons under versus near electrode regions (Fig. 2g, Independent two-sample t-test, n.s.: not significant). These results confirm that the CODE system preserves single-neuron imaging fidelity.

We next evaluated the long-term performance of the system. Following implantation, microglial fluorescence signal-to-background ratio (SBR) remained above 1.5 for at least four weeks (Fig. 2h), while the ECoG signal SNR consistently exceeded 10 throughout the recording period (Fig. 2i). By seven weeks post-implantation, more than 75% of electrodes remained functional (Fig. 2j). Electrode impedance, measured by EIS, remained below 0.8 MΩ at 1 kHz throughout the seven-week period (Supplementary Fig. 1f), demonstrating stable impedance and recording performance over extended experimental durations. Moreover, we directly evaluated electrophysiological artifacts under continuous and pulsed illumination conditions (Supplementary Fig. 2) and found that any light-induced effects were negligible with no discernible artifacts detected under the illumination conditions tested.

### Cortex-wide dynamic neuron ensembles associated with burst-suppression rhythm

Burst-suppression is a hallmark cortical activity pattern associated with deep anesthesia and unconsciousness. Isoflurane, a widely used inhaled anesthetic, reliably induces this pattern due to its rapid onset and reversibility. Here, we employed isoflurane to examine burst-suppression dynamics at single-neuron resolution using the CODE system.

**Fig. 3:**
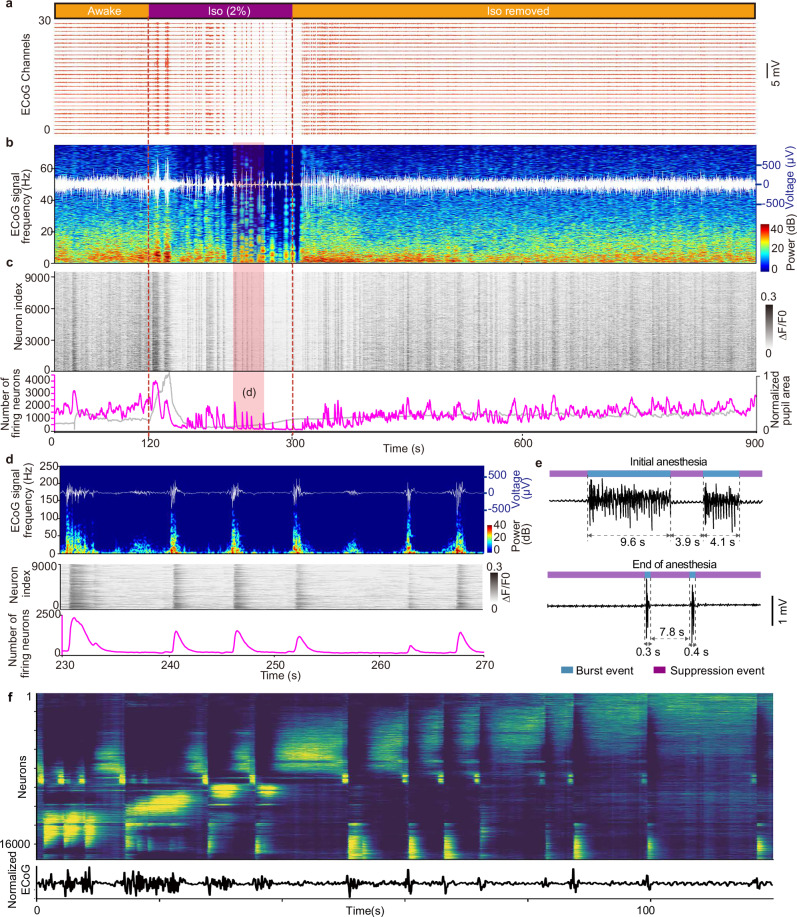
Cortex-wide dynamic neuron ensembles respond to isoflurane-induced burst-suppression rhythm in an on-off pattern. **a**–**c**. Multichannel ECoG signals (**a**) and the ECoG waveform and its corresponding spectrogram from a representative electrode (channel 25) (**b**) the fluorescence trace of cortical neurons during loss of consciousness, and the corresponding number of activated neurons across different consciousness stages and the normalized pupil area (**c**) changes throughout various isoflurane stages. **d** Enlarged view from (**a**–**c**) which is marked with pink. **e** Typical ECoG waveform in the initial and end of anesthesia shows characteristic burst suppression events. **f** Normalized rastermap of cortical calcium neuronal activity from a representative mouse (top), and corresponding normalized ECoG waveform of one channel (bottom) throughout the anesthesia period, showing neurons alternatively activated as an electrophysiological burst-suppression rhythm (see “Methods”). Notably, distinct groups of neurons can be observed in the rastermap: those in the upper portion are predominantly active during suppression phases, whereas those in the lower portion are preferentially activated during burst periods. Source data are provided as a Source Data file.

To monitor burst-suppression at both population and cellular scales, we recorded ECoG and calcium signals simultaneously in head-fixed mice under controlled modulation of isoflurane anesthesia (see “Methods”). Mice were positioned on a treadmill with a gas catheter placed near the nostrils, enabling precise isoflurane delivery and fine control over the animals’ level of consciousness. Each trial consisted of a 2-minute awake baseline, a 3-minute 2% isoflurane-induced anesthesia period, and a subsequent recovery period following anesthetic withdrawal. During this protocol, ECoG signals, calcium activity and pupil size were recorded concurrently across the dorsal cortex (Fig. 3a–c).

This multi-modal paradigm revealed pronounced state-dependent changes in cortical dynamics. Isoflurane onset was associated with a significant decrease in neuronal activity, evidenced by diminished ECoG signal amplitude, broadband spectral power suppression (Fig. 3a, b), a reduced number of active neurons (Fig. 3c), and significant pupil constriction (lower right in Fig. 3c). These effects gradually reversed during the recovery phase. Zoomed-in analysis (Fig. 3d and Supplementary Video 2) showed that burst onset was tightly associated with synchronous calcium transients. As anesthesia deepened, burst-suppression patterns became increasingly prominent, characterized by progressively shorter bursts and lengthened suppression intervals (Fig. 3e and Supplementary Fig. 3)—consistent with alternating periods of cortical excitation and quiescence.

To further investigate whether specific neurons were differentially engaged during burst and suppression events, we analyzed neuronal activity using a normalization- and correlation-based sorting method (Supplementary Fig. 4a–c; see “Methods”). This analysis identified two neuronal ensembles with distinct activity preferences: one exhibiting preferential activation during bursts, and the other during suppressions (Fig. 3f and Supplementary Fig. 4d). These two neuronal ensembles appear to be closely coupled to the electrical rhythms of burst-suppression, and display dynamically alternating activation patterns that accompany transitions between burst and suppression states. In addition, we tested different transgenic mouse lines targeting excitatory (CaMKII-Cre/Ai162d, Supplementary Fig. 5a) and inhibitory (Vgat-Cre/GCaMP8s, Supplementary Fig. 5b) neurons under anesthesia, illustrating the applicability of this approach to neuron-type-specific investigations.

### Dynamic recruitment and activation patterns during burst-suppression

**Fig. 4:**
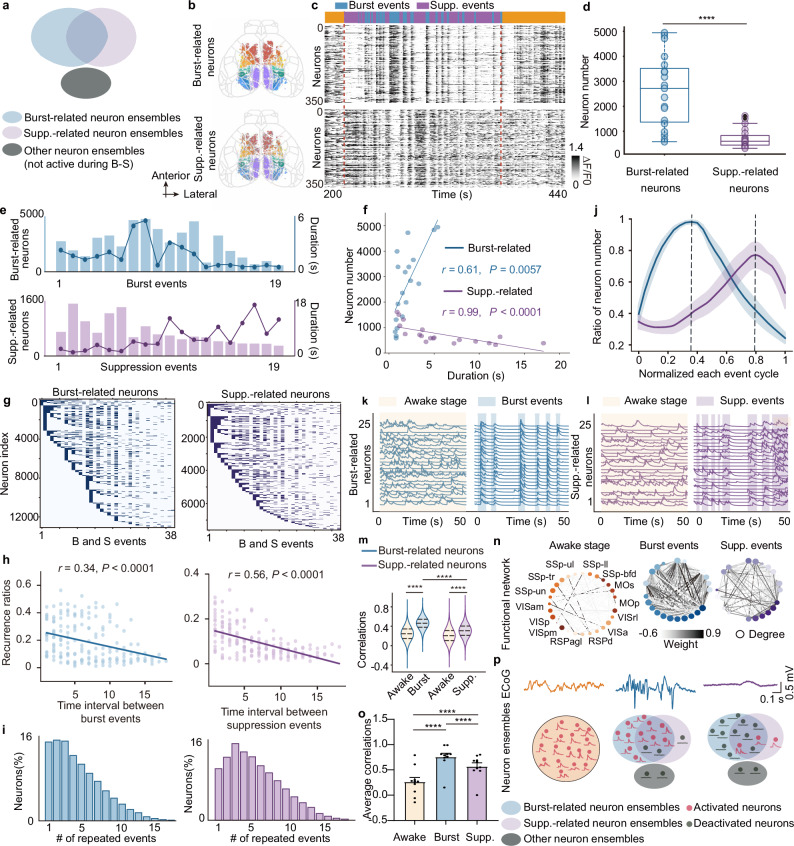
Dynamic recruitment of neuronal ensembles during burst and suppression events. **a** The schematic illustrates the classification of neurons into burst-related, suppression-related, and other neuron ensembles. **b** Two kinds of neurons distributed across wide cortex involving MOs, MOp, SSp-ll, SSp-ul, VISa, VISam, VISpm, VISp, RSPagl, RSPd regions that respond to burst events, suppression events, and awake stages. **c** The typical fluorescent trace of the two kinds of neurons. **d** Comparison of the number of different neuronal groups in each burst or suppression event. Box plots show the median (center line), the interquartile range (box), and whiskers extending to 1.5 × the interquartile range from the box limits. Circles represent individual burst or suppression events (*n* = 19 events). **e** Number of neurons activated during bursts (Burst-related neurons, top) and suppressions (Suppression-related neurons, bottom), showing rhythmic fluctuations synchronized with each phase in an experimental mouse, alongside the duration of each burst (blue) and suppression (purple) phase depicted with solid lines. **f** Correlation between the number of active neurons and event durations, showing the different modulation by the size of their respective active population between burst and suppression. Circle, individual event. r, Pearson correlation coefficient. *p*(burst) = 0.0057, *p*(supp) < 0.0001. **g** Neuronal ensemble dynamics of burst-related neurons (left) and suppression-related neurons (right), visualizing by newly recruited and recurrently recalled neurons across 38 successive events. **h** The relationship between recurrence ratio and time interval between burst events (left) and suppression events (right). r, Pearson correlation coefficient. *p*(burst) < 0.0001, *p*(supp) < 0.0001. **i** Number proportion of neurons responding repeatedly to burst (left) and suppression (right) events within fixed 10-frame segments to full event durations, illustrating inconsistent participation of the majority of neurons in all events. **j** The distribution of peak time of responsive neuron number within each burst or suppression across 4 mice (mean ± SEM). **k**,** l** Representative neuronal trace across different stages, showing synchronous burst neuron firing (**k**) and asynchronous suppression neuron firing (**l**). **m** The violin plot compares the average correlations within burst-related neurons and suppression-related neurons, respectively, across 19 events at different brain states. **n** The functional network at the brain region level in different stages. **o** Comparison of average functional correlations between burst events, suppression events and awake stages (mean ± SEM). **p** Schematic representation of ensemble-level connectivity governing the generation and modulation of local field potential features during burst-suppression transitions. Statistical significance was assessed using two-sided Mann-Whitney U tests for panels (**d**, **m** and o). ****: *p* < 0.0001; Source data are provided as a Source Data file.

To elucidate the dynamic organization of neuronal ensembles underlying burst-suppression transitions at single-cell resolution, we systematically analyzed their recruitment patterns, temporal activation patterns, and functional connectivity across distinct brain states. Neurons were classified across the entire burst-suppression cycles based on their firing behavior (Fig. 4a–c, see “Methods”), yielding three functional categories: burst-related neurons, active during high-voltage bursts; suppression-related neurons, active during inter-burst suppressions; and other neurons, inactive during either event. Burst- and suppression-related neurons were consistently observed across animals (Supplementary Fig. 6a). At the population level, these neuronal classes exhibited alternating on–off dynamics across burst-suppression cycles (Fig. 4c), confirming that distinct neuronal ensembles selectively encode different cortical states.

To examine ensemble dynamics over the full anesthesia session, we quantified the number of active neurons during individual burst and suppression events. Both ensembles exhibited time-varying sizes across events (Fig. 4d, e, Two-sided Mann-Whitney U rank test applied in comparing overall number of burst-related and suppression-related neurons with *W* = 330 and *P* < 0.0001, Supplementary Fig. 6a, b). Notably, burst duration and ensemble size gradually declined over time, whereas suppression periods lengthened while involving fewer active neurons (Fig. 4f and Supplementary Fig. 6c). This inverse relationship suggests that ensemble size correlates with event duration in a state-dependent manner. Importantly, this correlation persisted regardless of different temporal analysis windows—from short 10-frame segments to full event durations (Supplementary Fig. 6d).

Spatially, ensemble configurations varied across time and anesthesia depth. Neurons active during bursts (Supplementary Fig. 7a) and suppressions (Supplementary Fig. 7b) were broadly distributed across the cortex, with no fixed spatial pattern as anesthesia progressed (Supplementary Video 3). To track transitions across events, we visualized ensemble activity across 38 successive events. This analysis revealed a mix of newly recruited and recurrently recalled neurons in each phase (Fig. 4g). Notably, the recurrence probability decreased with longer inter-event intervals, indicating that recently recruited neurons were more likely to be recalled in the subsequent events (Fig. 4h). Such a time-dependent decline in reactivation likelihood suggests a gradual reduction in excitability or recruitment potential of both burst- and suppression-related neurons over successive events. Consistent with this, most neurons did not participate in all events; ~ 50–60% of burst- and suppression-related neurons responded fewer than five times (Fig. 4i), indicating dynamic and sparse participation.

Temporally, burst-related neurons activated early within bursts, peaking within the first 20–40% of burst duration (Fig. 4j). In contrast, suppression-related neurons exhibited delayed, gradual activation, with peak activity near 80% of the suppression phase. Burst-related ensembles showed a narrow activation peak, indicating highly synchronized firing, whereas suppression-related activity was more temporally dispersed, reflecting asynchronous dynamics. These activation patterns were consistent across animals (Supplementary Fig. 8a).

To probe the mechanisms underlying these distinct temporal and spatial dynamics, we examined pairwise functional connectivity during different events (Fig. 4k–n). Neurons tend to exhibit more synchronous activity during bursts (Fig. 4k, right), but asynchronously during suppressions (Fig. 4l, right). On the contrary, neuronal activity in the awake state appears randomly (Fig. 4k, l, left). Correlation analysis confirmed that burst-related neurons exhibited significantly stronger functional connectivity than suppression-related neurons, both of which exceeded correlation levels observed during wakefulness (Fig. 4m, Two-sided Mann-Whitney U rank test with *P* < 0.0001).

We further compared whole-brain network topology across awake, burst, and suppression states (Fig. 4n; Supplementary Fig. 8b, c and Supplementary Video 4). Both burst and suppression exhibited enhanced whole-brain connectivity and more defined modular network structures than awake states, with bursts showing the highest levels of global connectivity (Supplementary Fig. 8b, bottom; Supplementary Fig. 8c, top). Network topology during bursts also showed greater regional heterogeneity, featuring prominent hub nodes and densely connected modules (Fig. 4n, o, Two-sided Mann-Whitney U rank test with *P* < 0.0001; Supplementary Fig. 8c, bottom).

Together, these observations suggest that these distinct functional connectivity profiles underlie the divergent activation dynamics of burst- and suppression-related ensembles (Fig. 4p). During bursts, stronger intra-ensemble coupling promotes synchronized activity, giving rise to the large-amplitude ECoG waveforms that typify bursts. In contrast, suppression-related neurons are more weakly and diffusely connected, resulting in asynchronous calcium dynamics and attenuated electrophysiological signals. These findings provide a mechanistic framework linking ensemble-level architecture to large-scale cortical state transitions during burst-suppression.

### Propagation of neuronal activity during suppression-to-burst transition

Given our observation that burst-related neurons tend to activate earlier than suppression-related neurons within each event, we next examined how neuronal activity is spatially distributed across the cortex during suppression-to-burst transitions. We characterized cortex-wide dynamics at both the single-neuron and population levels by leveraging the combination of cortex-wide maps and millisecond-scale dynamics.

**Fig. 5:**
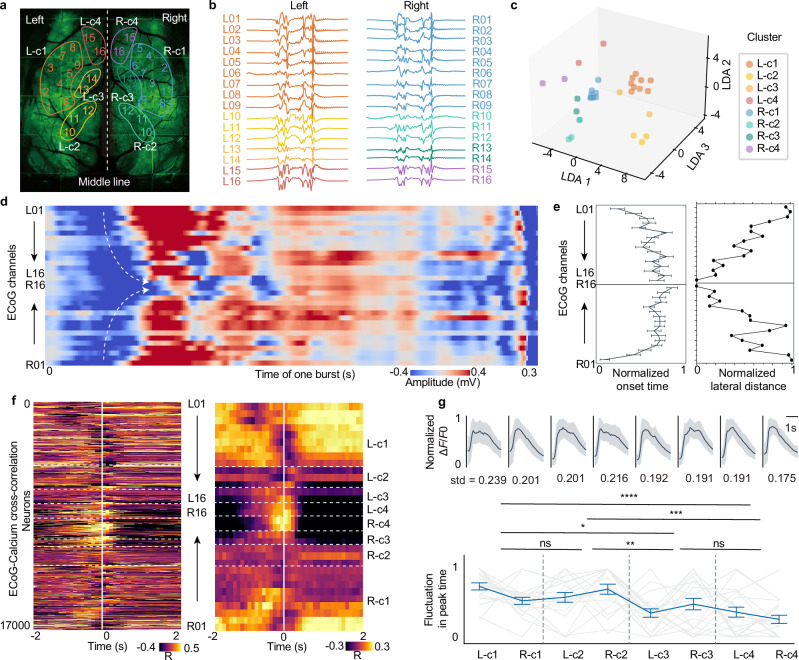
Propagation of neural activity from sensory to motor cortex during suppression-to-burst transitions. **a**–**c** Categorization of the 32-channel electrode array into eight groups based on their anatomical location (**a**) verified by supervised clustering of electrodes (**c**) according to their electrophysiological activity profiles (**b**). **d** Organized 32-channel recordings from lateral sensory to frontal motor regions during burst onset, showing a significant directional propagation of burst waveforms. **e** Burst onset latency for each channel across all bursts in a typical mouse (mean ± SEM, *n* = 19) (left) and corresponding lateral cortical position relative to the midline (right), confirming a robust inverse correlation between them. **f** Cross-correlation between ECoG and individual neuronal activities (left) within a ± 2 second window surrounding burst onset, and averaged cross-correlations for each electrode (right), showing synchronized neuronal-ECoG coupling with distinct temporal lags between medial and lateral cortical regions. **g** The averaged calcium activity traces (top), along with the statistical distribution of neuronal phase delay from burst onset to peak calcium response for each cluster (bottom). Data are presented as mean ± SEM (*n* = 19). Gray lines, individual burst event. Two-sided Wilcoxon signed-rank test is used in (**g**). *p*(c1 vs c2) = 0.7093, *p*(c1 vs c3) = 0.0286, *p*(c1 vs c4) < 0.0001; More detailed statistical information recorded in Source Data file. ns: not significant (*p* > 0.05), *: *p* < 0.05, **: *p* < 0.01, ***: *p* < 0.001, ****: *p* < 0.0001; Source data are provided as a Source Data file.

We first grouped the 32-channel electrode array based on anatomical location, and assessed this grouping via supervised clustering of electrophysiological activity, resulting in eight spatially coherent clusters (Fig. 5b, c). Permutating these clusters from lateral sensory to frontal motor regions revealed a consistent propagation of burst waveforms from lateral to medial cortex at each event onset (Fig. 5d and Supplementary Fig. 9a). Bursts initiated in bilateral lateral sensory cortices and propagated medially and anteriorly with measurable temporal delays. Quantitative analysis confirmed a robust inverse correlation between burst onset latency and lateral cortical position relative to the midline (Fig. 5e and Supplementary Fig. 9b).

To examine how single-neuron activity aligned with this propagation pattern, we computed cross-correlations between ECoG signals and calcium traces from neurons located within a 13 μm radius of each electrode, within a ± 2 s window centered on burst onset (Fig. 5f, left, Supplementary Fig. 10a, see “Methods”). Averaged cross-correlations revealed stronger ECoG–neuron coupling in medial motor cortex, while lateral sensory regions showed phase offsets and lower synchrony (Fig. 5f, right). Mapping these spatial coupling patterns onto the Allen Brain Atlas revealed a low–high–low coupling strength transition around electrodes L-15/16 and R-15/16 (Supplementary Fig. 10b).

We then visualized calcium activity from neurons within each electrophysiologically defined cluster (Fig. 5g, Two-sided Wilcoxon signed-rank test between lateral sensory cortex and medial motor cortex with *W* = 92 and *P* < 0.0001). Neurons in the lateral sensory cortex (L-c1 and R-c1) showed broad, dispersed activation peaks, whereas those in the medial motor cortex (L-c4 and R-c4) exhibited sharply peaked responses time-locked to burst onset. These firing patterns mirrored the lateral-to-medial propagation seen in ECoG and suggested a progressive increase in synchrony across the cortical surface. Extending this analysis across four brain states—awake, burst, suppression, and recovery—revealed that ECoG–neuron coupling was significantly stronger during burst and suppression states than during awake or recovery phases (Supplementary Fig. 11a–c), indicating enhanced temporal coordination during unconscious states.

Building on our characterization of cortex-wide electrical and calcium dynamics during burst–suppression, we further examined millisecond-scale spatiotemporal patterns in ECoG signals aligned to cortex-wide maps. During the suppression-to-burst transition, ECoG activity exhibited temporally ordered spatial patterns across channels (Supplementary Fig. 12a, b); in contrast, same analysis applied to ECoG signals during the sustained burst phase revealed more spatially heterogeneous patterns (Supplementary Fig. 12c). These observations indicate that the spatiotemporal organization of ECoG activity differs across phases of burst–suppression, with more structured patterns emerging transiently around state transitions.

Together, these findings reveal a robust lateral-to-medial propagation of neuronal activation during suppression-to-burst transitions, accompanied by increasing synchrony between local field potentials and single-neuron calcium dynamics. This seemingly paradoxical synchronizing-while-propagating phenomenon may emerge from the denser and more cohesive network of burst-related neurons, in contrast to suppression-related neurons. Previous studies have identified the prefrontal cortex as a central hub for multisensory integration^42,43^. During sleep or anesthesia, interactions between the visual cortex and frontal areas such as the anterior cingulate cortex (ACA), as well as information flow from the frontal to retrosplenial cortex (RSC), become disrupted^44,45^. Burst-related synchronization reflects a globally coordinated spiking pattern across the cortex, in which cortical activity shifts from a relatively suppressed state to a more active state during bursts. These findings bridge cellular-scale dynamics with mesoscale electrical signatures and offer a mechanistic framework for understanding the spatiotemporal architecture of burst-suppression.

### Cross-modal signal prediction using shared variance components

Building on our previous findings—showing that burst-associated neuronal activity exhibits stronger synchrony, cortex-wide propagation, and tight coupling between local field potentials and single-cell dynamics—we next asked whether such structured population activity enables reliable inference across recording modalities, and how this cross-modal predictability varies across brain states.

**Fig. 6:**
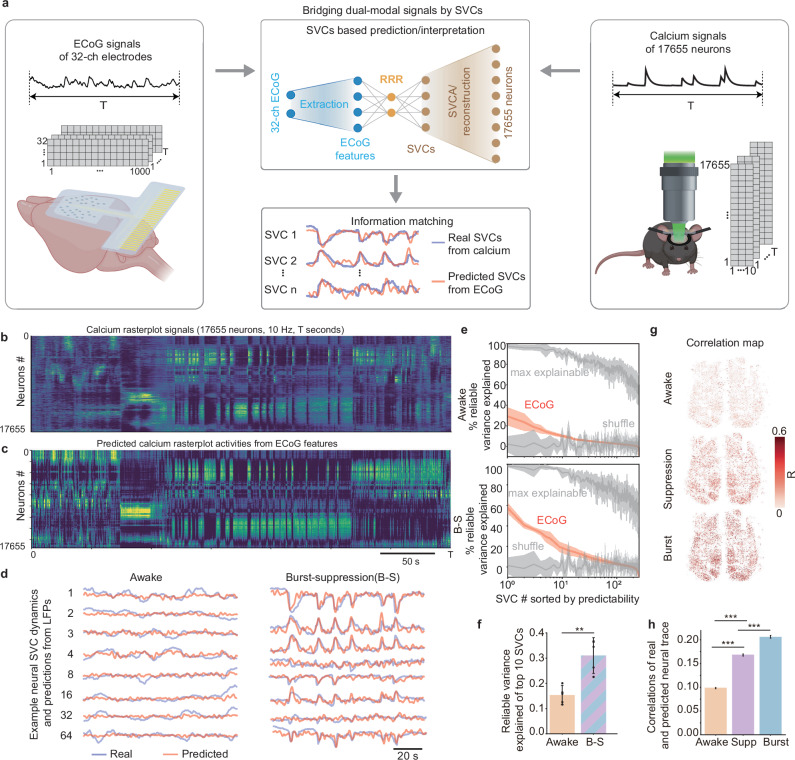
Bridging dual-modal signals via shared variance components (SVCs). **a** Schematic of the data processing pipeline illustrating how SVCs serve as quantitative intermediates for predicting and interpreting calcium activity (right panel) from ECoG signals (left panel). Specifically, SVCs predicted from ECoG signals using reduced-rank regression (RRR) were reconstructed into the neuronal space (middle panel). Created in BioRender. Dai, X. (2026) https://BioRender.com/7q3qess. **b** Original neuronal calcium traces. **c** Predicted calcium activity inferred from ECoG signals. **d** Comparison of real (calcium-derived) and predicted (ECoG-derived) neural SVCs during awake and burst-suppression events. **e**,** f** Quantitative evaluation at the SVC level. **e** Cross-validated fraction of variance explained for successive SVCs predicted from ECoG signals (red), alongside the corresponding reliable variance (gray; i.e., maximum explainable variance). The top and bottom panels represent awake and burst-suppression conditions, respectively. **f** Summary statistics for (**e**): comparison of explained reliable variance for the top 10 SVCs between awake and burst-suppression events (*n* = 3 mice). **g**, **h** Quantitative evaluation at the single-neuron level. **g** Correlation heatmaps between real neuronal activity and reconstructed activity derived from predicted SVCs across the dorsal cortex, shown for the awake (top), suppression (middle), and burst (bottom) states. The maps illustrate a progressive increase in prediction accuracy and interpretability across states, following the pattern: burst > suppression > awake. **h** Summary statistics for (**g**): comparison of correlation values between real and predicted neural traces across awake, burst, and suppression states. Each data point represents an individual neuron. A total of *n *= 17,655 neurons were recorded from a single mouse in a single imaging session. Statistical comparisons between groups were performed using independent two-sample *t* tests, all tests were two-sided. **: *p* < 0.01, ***: *p* < 0.001; Error bars: SEM. Source data are provided as a Source Data file.

To address this, we established a computational framework to predict population-level calcium dynamics from mesoscopic ECoG signals (Fig. 6a; see “Methods”). This framework combines shared variance component analysis (SVCA)^46–48^ (Supplementary Fig. 13a, b) with a predictive model, reduced rank regression (RRR)^46,49^. First, we extracted 18 temporal and spectral features from each of the 32 ECoG channels, yielding a 576-dimensional feature space. In parallel, SVCA was applied to a calcium imaging dataset (> 17,000 neurons), reducing dimensionality while preserving key population-level variance. Crucially, SVCA is invertible, allowing reliable reconstruction of single-neuron activity from low-dimensional SVC components (SVCs) with minimal information loss (Supplementary Fig. 14a, b). We then trained an RRR model to map ECoG features onto calcium-derived SVCs, which were subsequently used to reconstruct predicted single-neuron calcium traces. The resulting predicted calcium raster (Fig. 6c) closely matched the ground truth (Fig. 6b), and this result was consistent across animals (Supplementary Fig. 14c).

To quantify the accuracy of this cross-modal prediction across different brain states, we assessed both the fraction of reliable variance captured by SVCs (Fig. 6d–f) and the correlation between actual and predicted neuronal activity (Fig. 6g, h). Predicted SVCs aligned more closely with true SVCs during burst-suppression states compared to the awake state (Fig. 6d). Notably, the leading SVC component accounted for only ~ 30% of the reliable variance during the awake state (Fig. 6e, top), but approximately 60% during burst-suppression (Fig. 6e, bottom). Across animals, the top 10 predicted SVCs explained significantly more reliable variance during burst-suppression than during the awake state (Fig. 6f, Independent two-sample *T* test with *P* < 0.01). At the single-neuron level, spatial correlation heatmaps (Fig. 6g) and averaged correlation values (Fig. 6h, Independent two-sample T-test with *P* < 0.001) confirmed that ECoG-to-calcium prediction accuracy was highest during bursts and lowest in the awake states.

Together, these results establish a principled cross-modal inference framework based on shared variance components. The SVCs serve as a low-dimensional representation of population dynamics and provide a quantitative basis for evaluating the interpretability between optical and electrical signals. Critically, the ability to predict single-neuron calcium activity from mesoscale ECoG signals is highly state-dependent—peaking during bursts due to elevated synchrony and connectivity, and declining during asynchronous awake states. These findings suggest that burst-related activity reflects a highly organized cortical regime in which macro- and micro-scale signals converge. The state-dependent variation in cross-modal prediction accuracy likely reflects the fundamental shift in the complexity of cortical dynamics between unconscious (burst-suppression) and awake states. The higher predictability during anesthesia is consistent with a collapse into lower-dimensional, more stereotyped dynamics, while the lower predictability in the awake state mirrors the high-dimensional, complex neural activity associated with consciousness.

## Discussion

In this study, we investigated the trans-scale organization of neuronal activity during burst-suppression transition using a cortex-wide optical-electrical dual-modal approach. By combining simultaneous imaging of over 17,000 single neurons and 32-channel ECoG recording across the dorsal cortex with high spatiotemporal resolution, we uncovered three previously underappreciated features of burst-suppression dynamics: (1) the presence of distinct neuronal populations selectively active during burst versus suppression phases, (2) alternating synchronization and desynchronization patterns of neuronal firing aligned with burst-suppression waveform structure, and (3) regionally heterogeneous, rapidly propagating activity during suppression-to-burst transitions. Together, these findings challenge prevailing assumptions about unconscious brain states as a globally uniform and passive state, instead revealing a structured spatiotemporal reorganization of cortical activity during anesthesia.

The CODE system—by integrating high-resolution optical and electrical recordings across the entire cortex—enabled identification of two functionally distinct neuronal ensembles that are alternately recruited during bursts and suppressions. While suppressions have traditionally been interpreted as near-complete cortical quiescence^50^, inferred from flat EEG and global metabolic decline^51,52^, our data reveal that suppression phases are not periods of uniform silence. Instead, we observed globally distributed, alternating ensemble dynamics reminiscent of competing cortical coalitions^53^. In particular, we identified a previously undefined population of “suppression-related” neurons, whose weak and asynchronous activity likely renders them difficult to detect using recording with limited spatial coverage or smaller neuronal sampling. These neurons’ selective activation during suppression events indicates that suppression is not a state of complete silence, as implied by the macroscopic electrical signal.

We further found that the degree of neuronal synchronization was closely linked to mesoscopic field potential features. High synchrony and functional connectivity gave rise to large-amplitude ECoG events, whereas asynchronous activity during suppressions led to signal cancellation and flatline waveforms. This finding prompted us to investigate whether ECoG signals can reflect underlying neuronal dynamics in a state-dependent manner. To test this, we developed a predictive framework linking ECoG features to calcium activity via SVCs. Cross-modal inference was significantly more accurate during bursts than suppressions or wakefulness, highlighting enhanced macro–micro signal coherence in synchronized states. In contrast, during wakefulness, ongoing sensory and cognitive processing likely contributed to decorrelated activity. Therefore, the degree to which large-scale population activity can be predicted from macroscopic ECoG signals may provide an indirect, yet informative, signature of the complexity of cortical dynamics, which is intrinsically linked to brain state. Recent evidence suggests that high-frequency bands can encode detailed neural activity originating from deeper cortical layers—information that is inaccessible to surface calcium imaging^28^. Our current ECoG-based predictions, especially during awake states when neural activity becomes high-dimensional and less correlated, may be constrained by the spatial resolution and limited bandwidth of the standard ECoG array used here. Looking forward, the use of next-generation, high-density microelectrode arrays with smaller electrode sites and broader recording bandwidths may allow future studies to capture these high-frequency signals, including spiking-related activity and high-gamma oscillations. Such advances could enable more accurate cross-modal inference of neural population dynamics, even during wakefulness, by providing a more complete electrophysiological representation of local neural computation.

In addition, the trans-scale spatiotemporal resolution of CODE allowed us to track the global propagation of activity during suppression-to-burst transitions. Previous work has reported traveling waves during seizures^12,13^, other pathological states^54^, and during awake sensory processing^55^, yet cortical propagation under anesthesia remained unexplored. We found that bursts consistently initiate in lateral sensory or visual areas and propagate medially toward motor regions, forming a peripheral-to-central activation sequence. Remarkably, this propagation was accompanied by increasing synchrony between calcium and ECoG signals, revealing a “synchronizing-while-propagating” phenomenon. This observation directly challenges the view of burst-suppression as a globally synchronous state and suggests that cortical transitions—even under deep anesthesia—retain organized spatial dynamics. Such propagation reliably accompanies transitions from suppression to bursts, suggesting that cortical activity is not random but instead follows structured dynamics during anesthetized unconsciousness.

By combining the recording of large population calcium activity and ECoG across wide cortical areas, CODE allowed us to define two distinct neuronal ensembles and compare their distribution, firing patterns, and dynamics. Moreover, we observed and predicted a propagation pathway during burst onset, from bilateral sensory to medial motor regions, across different brain states. Our work pioneers a holistic research framework for full-cortical multimodal studies, introducing detection and analysis methods to investigate neural activity transitions during burst suppression. In addition, beyond its advantages for in vivo multimodal recording, the CODE system also provides a unique solution for aligning in vivo and ex vivo data—a persistent challenge in histological studies. Traditional tissue processing causes deformation and swelling, complicating the correlation of functional recordings with post hoc molecular labeling. In the CODE platform, however, the embedded electrodes and traces serve as stable fiduciary markers after fixation and clearing. This inherent registration enables accurate spatial mapping between in vivo activity and ex vivo cellular or molecular readouts, offering a promising approach for linking neural dynamics to genetically defined cell types. (Supplementary Fig. 15)

The CODE system was essential for revealing these dynamic processes—from ensemble alternation and synchrony transitions to cortex-wide propagation. By enabling simultaneous large-scale single-cell imaging and multi-site electrophysiology, CODE provides a multi-scale perspective on brain dynamics that is unattainable with single-modality or small-FOV methods. Future technical advances—such as increasing electrode density through vertical stacking^56,57^, implementing active multiplexing^58^, or incorporating cell-type-specific fluorescent labeling^59–62^—could further refine the spatial and functional granularity of this platform. Altogether, CODE opens avenues for dissecting state transitions, probing cortical coordination, and guiding neuromodulatory interventions in altered or pathological brain states.

## Methods

### Mouse

All animal experiments were conducted in accordance with the ethical guidelines of Tsinghua University and were approved by the Institutional Animal Care and Use Committee (IACUC) of Tsinghua University, Beijing, China. Mice were housed in the Laboratory Animal Research Center of Tsinghua University under controlled environmental conditions, including a constant temperature of 24 °C, relative humidity of 50%, and a 12 h light/dark cycle (lights on from 7:00 a.m. to 7:00 p.m.), to ensure animal welfare and compliance with institutional standards.

Experimental mice (8–16 weeks old, 20–30 g) were generated by crossing Rasgrf2-2A-dCre mice (JAX 022864) with Ai148 (TIT2L-GC6f-ICL-tTA2)-D mice (JAX 030328), both obtained from The Jackson Laboratory. Mice of both sexes were randomly selected for the experiments. Thy1-YFP transgenic mice (JAX #003709) were used to perform in vitro tissue clearing and fluorescence imaging to map the in vivo signals. Transgenic mice expressing GCaMP specifically in excitatory (CaMKII-Cre/Ai162d) (JAX #005359, JAX #031562) and inhibitory (Vgat-Cre/GCaMP8s) (JAX #028862, JAX #037717) were also used. Trimethoprim (TMP; 0.25 mg/g body weight; Sigma) was administered via intraperitoneal injection for two consecutive days to mice of both sexes.^36^

### Surgery

Surgical procedures for neural interface implantation were adapted from established protocols^63^, with modifications to accommodate our transparent CODE interface. All surgical instruments and work surfaces were sterilized using high-temperature autoclaving or ethanol disinfection to maintain aseptic conditions.

Mice were anesthetized with 2% isoflurane and secured in a stereotaxic apparatus (model 68018, RWD Life Science). After scalp removal, the skull was carefully excised to expose the dorsal cortex and accommodate the full field of view required for calcium imaging. The cortical surface was rinsed with sterile saline before interface placement.

A transparent ECoG neural interface, replacing the traditional crystal skull window, was aligned and placed directly over the cortex. The interface was sealed to the skull rim using tissue adhesive (Vetbond^TM^, 3 M). A stainless steel screw implanted into the frontal bone served as a ground reference electrode. A custom-designed head-post was affixed to the remaining skull using dental acrylic, providing mechanical stability for subsequent recordings.

### Perfusion and tissue harvest

After implanting the CODE device onto the thy1-YFP mouse to ensure the device attached stably to the brain, mice were deeply anesthetized and transcardially perfused with approximately 20 ml of ice-cold 0.9% phosphate-buffered saline (PBS), followed by 20 ml of freshly prepared 4% paraformaldehyde (PFA) in PBS.

The head was then post-fixed in 4% PFA at 4 °C for 24 h. To preserve the precise spatial relationship between the electrode array and the underlying dorsal brain tissue, the ventral adhered brain tissue and skull were carefully dissected en bloc. All surrounding soft tissue was meticulously removed, leaving the skull - electrode - brain complex intact.

### Brain tissue clearing

All the brains are cleared by the CLARITY method with the Polymerization and electrophoresis cleaning equipment(Logos System)^64^. First, 12.5 mg of the polymerization initiator was added to a centrifuge tube containing 5 ml hydrogel solution and mixed thoroughly. The whole-brain tissue was immersed in the mixture and placed in a refrigerator at 4 °C for 24 h. Then, the centrifuge tube with the lid removed was placed in the polymerization system to react for 3 h at 37 °C and 90 kPa. Next, the whole-brain tissue was removed and eluted three times with PBS for 1 h at a time. The whole-brain tissue was then placed in the electrophoresis cleaning system at 37 °C for 8 h at a current of 1.2 A and a peristaltic pump speed of 100 rpm. Next, the sample was cleaned for 24 h with 1 × PBS in a 37 °C constant temperature shaker (40 rpm), and refresh above 10 times. Finally, the sample was immersed in 5 mL mounting solution (37 °C and 40 rpm) overnight, and refresh the solution one time. After that, the whole-brain tissue was completely transparent. Finally, the sample was placed in 30 mL mounting solution to get better RI matching.

The cleared brains were imaging with LiTone XL light-sheet microscope (Light Innovation Technology), which was equipped with two-sided 5 × /NA0.14 illumination optics and 10 × /0.6 NA detection optic. The volume data was acquired at 1.36 μm × 1.36 μm × 3 μm pixel size. Further image processing was performed by LitScan 3.3.0 and Imaris11.1 software.

### Ex vivo fluorescence imaging

The cleared, index-matched specimen was imaged using a light-sheet fluorescence microscope (UltraMicroscope II, Miltenyi Biotec, equipped with a Lavision BioTec LiT XL light-sheet unit). The sample was suspended in an imaging chamber filled with RIMS. A 488 nm laser was used to excite YFP fluorescence, which was collected through a suitable emission filter (e.g., 525/50 nm). High-resolution z-stacks were acquired with a step size of 2–5 µm, capturing the complete 3D structure of the labeled neurons in relation to the transparent electrode sites and conductive traces embedded within the sample.

### Transparent electrode array fabrication and characterization

#### ECoG Electrode array fabrication

The fabrication of the transparent electrode array involved the sequential deposition and patterning of gold (Au), indium tin oxide (ITO), and a top-layer SU-8 encapsulation. A clean 0.5 mm-thick, 4-inch glass wafer was first coated with AZ-601 photoresist and patterned via standard photolithography. After plasma treatment to enhance surface cleanliness and adhesion, a 5 nm chromium adhesion layer and a 100 nm gold layer were deposited via thermal evaporation. A lift-off process using N-methyl-2-pyrrolidone (NMP) removed excess metal, resulting in gold interconnects as narrow as 2 μm, minimizing visual obstruction of underlying neurons.

To define the electrode sites, ITO was deposited using magnetron sputtering due to its high optical transparency and electrical conductivity^65,66^. An annealing step was applied to reduce sheet resistance and improve optical transmittance. An SU-8 layer (SU-8 2002) was then spin-coated, photolithographically patterned, and hard-baked at 190 °C to insulate the interconnects while exposing the ITO electrode sites. Each array was laser-cut to match the shape of the cranial window, ensuring precise cortical coverage.

For electrical interfacing, the electrodes were bonded to a flexible flat cable (FFC) using anisotropic conductive film (ACF) and hot pressing. The FFC was then connected to a zero-insertion-force (ZIF) connector on a custom-designed printed circuit board (PCB), which interfaced with a 32-channel Plexon headstage for signal acquisition (Supplementary Fig. 1).

#### ITO Sputtering, patterning, and annealing

ITO deposition was performed using a magnetron sputtering system (ULVAC, QAM-4W-STS) and patterned via lift-off. AZ-601 photoresist was spin-coated and patterned via photolithography (MA/BA8 Gen3, SUSS). After plasma treatment, ITO was sputtered at a base pressure below 1.0 × 10⁻⁵ Pa using a gas mixture of Ar (100 sccm) and O₂ (2 sccm), with a DC power of 100 W. The film thickness was controlled at 100 nm by adjusting the deposition duration.

After deposition, the wafer was immersed in NMP to lift off unwanted ITO, yielding cleanly defined electrode sites. The patterned wafer was then annealed under nitrogen flow at 450 °C for 2 h (ANNEALSYS, AS-One100). This step reduced sheet resistance to below 40 Ω/ϒ and improved optical transmittance.

#### PEDOT Spin-coating and patterning

To reduce electrode impedance while preserving transparency, a PEDOT:PSS layer was applied via spin-coating and patterned by reactive ion etching (RIE)^23,24,67^ The solution contained 95 wt% PEDOT:PSS (PH1000, Clevios), 5 wt% ethylene glycol, and two drops of dodecylbenzenesulfonic acid (DBSA), and was sonicated for 20 min before being filtered through a 0.8 or 1.2 μm PTFE membrane. The first 10 drops were discarded to ensure consistency. Immediately prior to spin-coating, 1 wt% (3-glycidyloxypropyl)trimethoxysilane (GOPS) was added.

Spin-coating was performed in two stages: 500 rpm for 5 s followed by 1500 rpm for 30 s. The coated substrates were then baked on a hot plate at 110 °C for 60 seconds. This coating-baking cycle was repeated three times to achieve a final film thickness of approximately 200 nm. Subsequently, the wafer was hard-baked at 100 °C for 1 h and soaked in deionized water overnight to enhance film stability.

For patterning, AZ-601 photoresist was spin-coated and patterned via photolithography. RIE was performed using a SHL 100-RIE system (Beijing SHL Semi. Equipment Co., Ltd) with a gas mixture of CF_4_ and O_2_ (flow rates: 5 sccm and 100 sccm, respectively) under a pressure of 40 mTorr and 150 W RF power. Residual photoresist was removed with acetone, completing the patterning.

#### Electrochemical Impedance Spectroscopy and Cyclic Voltammetry (CV)

Electrochemical impedance spectroscopy (EIS) and cyclic voltammetry (CV) were performed using a PalmSens 4.0 Potentiostat/Galvanostat/Impedance Analyzer (PalmSens, Netherlands), operated with PS Trace 5.9 software. A standard three-electrode configuration was used, comprising an Ag/AgCl reference electrode, a platinum wire counter electrode with a large surface area, and the test sample as the working electrode. All measurements were conducted in 1 × phosphate-buffered saline (PBS, pH 7.4) at room temperature.

EIS measurements were carried out across a frequency range of 0.1 Hz to 10 kHz, with ten data points per decade to ensure high resolution. CV measurements were performed from – 0.4 V to + 0.8 V at a scan rate of 0.05 V/s, beginning from the open-circuit potential and initially scanning in the positive direction. To ensure repeatability and reliability of results, each sample underwent a minimum of three complete scan cycles.

#### Signal-to-Noise Ratio (SNR) calculation for ECoG signals

The Signal-to-Noise Ratio (SNR) of the ECoG recordings was quantified in the frequency domain using the power spectral density (PSD). For each channel, PSD estimates were obtained using Welch’s method with 1-s Hamming windows and 50% overlap. The SNR was computed as:

1 $${SNR}=10\times {\log }_{10}(\frac{{P}_{s}}{{P}_{n}})$$

where ${{{{\rm{P}}}}}_{s}$ represents the average signal power within the frequency band of interest (1–100 Hz), corresponding to the range containing physiologically relevant ECoG activity. Noise power P_*n*_ was estimated from the same recording by calculating the mean PSD within a high-frequency band (500–1000 Hz), where neural activity is negligible, and the spectrum reflects baseline noise. This high-frequency band is commonly used to approximate the noise floor in electrophysiological recordings^68^. The resulting SNR provides a channel-specific measure of recording quality and was used for data inclusion and quality assessment.

### Head-fixed imaging and electrophysiological recordings

#### Mouse habituation

The recovered mice were head-fixed on a custom-built treadmill setup (Labmark) that permitted free movement of the limbs while stabilizing the head. To acclimate the mice to the apparatus, they underwent head-fixation training for 1 h per day over three consecutive days prior to experimental sessions involving modulation of consciousness.

#### RUSH System imaging

The RUSH imaging system^32^ consists of 35 sCMOS cameras arranged in a matrix to simultaneously capture brain-wide calcium events. The spherical field is divided into 5 × 7 sub-fields of view (sub-FOVs), which were then imaged through a relay lens array and 35 CMOS cameras. The RUSH imaging platform enables a 10 × 12 mm^2^ field of view with a uniform resolution of ~ 1.20 μm. The numerical aperture (NA) is 0.35. It utilizes a fluorescence excitation wavelength of 470 ± 20 nm and detects emissions at 525 ± 20 nm. The imaging frame rate was set at 10 Hz.

#### Two-photon imaging

A two-photon synthetic aperture microscopy (2pSAM)^41^ with needle-like beams to obtain 3D subcellular imaging across a volume of 688 × 88 × 100 μm^3^ at 30 volumes/s, corresponding to a data throughput of 1.57 giga-voxels/s/channel, was used to capture the detail fluorescence under the electrode and glass. This system supports imaging of subcellular dynamics at a millisecond scale for over 100,000 large volumes in deep tissue, with three orders of magnitude reduction in photobleaching. The FOV is 700 × 700 μm^2^, the resolution is 0.48 μm in the lateral axis and 1.3 μm in axial axis.

#### ECoG Signals acquisition

Neural electrical signals were acquired using a 32-channel Plexon headstage connected to the OmniPlex Neural Recording Data Acquisition System (Plexon, USA). The electrical signals were amplified, filtered, and digitized using a 32-channel Plexon headstage. Ground/reference wires were attached to stainless steel screws, drilled into mice skull. The sampling rate was set at 40 kHz and down sampled to 1 kHz for ECoG signals analyzing.

#### Data synchronization

Calcium imaging, electrophysiology, and pupil-tracking data streams were synchronized using transistor-transistor logic (TTL) pulses. A TTL pulse was triggered with each image frame and simultaneously recorded by the OmniPlex system to ensure precise temporal alignment across modalities.

### Large-scale neuron analysis

#### Calcium signal extraction and preprocessing

Two-photon calcium imaging data were processed using the CNMFE algorithm for initial neuron segmentation. Post-processing steps were implemented via custom MATLAB scripts. Raw fluorescence traces were high-pass filtered at 0.05 Hz, then denoised and deconvolved using the OASIS algorithm implemented in CaImAn^69^. Neurons with inferred spike activity greater than 0.5 were considered to have fired a spike. A neuron was considered active when its inferred spike value exceeded 0.5. During burst events, neurons with continuous firing for at least 200 ms were classified as active participants.

#### Preprocessing of electrophysiological data

ECoG signals were first low-pass filtered using a fourth-order Butterworth filter with a 300 Hz cutoff. To eliminate 50 Hz power-line interference and harmonics, a notch filter was applied. The power spectral density and spectrogram was computed using the “pwelch” function, incorporating a Hanning window of variable length based on the target frequency.

#### Identification of structured neuronal activity using Rastermap

The Neuronal Rastermap Algorithm was employed to process raw calcium signals and identify regular neuronal activity during burst-suppression periods. The process included the following steps (Fig.3f): Raw calcium traces were normalized across the neuron axis at each time point. This normalization enhanced the visibility of suppression-period dynamics by compensating for fluorescence fluctuations. Correlation-based sorting was performed using the Rastermap algorithm^70^, which arranged neurons by shared temporal structure, revealing functionally organized activity patterns.

#### Classification of Burst- and Suppression-Associated Neurons

We segmented the anesthetized state into discrete burst and suppression events (Fig. 3f) and classified all neurons into three categories (Fig. 4a). A neuron was labeled as burst- or suppression-related if it exhibited significantly elevated activity during at least one burst or suppression event, respectively, compared to both its own baseline (5 frames prior) and overall mean activity (using scipy.stats.mannwhitneyu for the significance test, requiring *p*-value less than 0.001). Neurons not responsive during any event were categorized as “other.” Representative activity patterns and ensemble dynamics were visualized in Fig. 4b, c and Supplementary Fig. 4a.

To compare the activity of burst-related and suppression-related neurons, the number of these neuronal types are compared across 5 mice (Fig. 4d). The Two-sided Mann-Whitney U rank test revealed the significant difference between burst-related and suppression-related neurons. We quantified both the duration and number of active neurons in each burst or suppression event across the anesthesia stage (Fig. 4e, Supplementary Fig. 6b), limiting neuron counts to 10-second intervals for better comparability. In addition, we conducted the least squares method to establish a linear correlation between the duration of burst (or suppression) events and the number of responding neurons, labeled the fitted correlation coefficients (*r*) and *p*-values (*P*) (Fig. 4f and Supplementary Fig. 6c). We also illustrated the temporal evolution of the spatial distribution of these two neuronal types over time (Supplementary Fig. 7).

#### Ensemble dynamics across burst-suppression events

Throughout the anesthesia stage, neurons exhibited dynamic activation patterns across different burst or suppression events, with observable transitions between burst-related neurons and suppression-related neurons. To characterize these dynamic responses of neurons, we analyzed the repetitive responses from both burst-related and suppression-related neurons. We sorted each neuron category by recruitment and recall patterns across multiple burst or suppression events (Fig. 4g). To quantify neuronal activation behavior, we quantitatively assessed the relationship between the proportion of neurons reactivated in subsequent burst or suppression events and the temporal interval between events, followed by linear regression analysis with the fitted correlation coefficients (*r*) and *p*-values (*P*) (Fig. 4h). To quantify the likelihood of reactivation of newly recruited neurons, we calculated a recurrence ratio matrix, where the element $[i,j]$ represents the proportion of neurons that were newly recruited in event i and subsequently recalled in event j. Specifically,

2 $${{Recurrence}\,{ratio}}_{[i,j]}=\frac{{N}_{{ij}}}{{N}_{i}}$$

${N}_{i}$ refereed to the recruited neurons in event $i$, ${N}_{{ij}}$ refereed to the recruited neuron number in event $i$ that are recalled in $j$. We analyzed how this recurrence ratio varied as a function of the time interval between different event pairs (either burst or suppression), fitting correlation coefficients (*r*) and *p*-values (*P*) to evaluate the relationship. Separate analyses were conducted for burst-burst event pairs and suppression-suppression event pairs. The proportion of reactivated neurons in subsequent events was also quantified and subjected to linear regression analysis to assess temporal trends, with analyses performed independently for burst and suppression events.

#### Quantifying neuronal participation and activation timing

To quantify differences in neuronal dynamics across burst and suppression events, we conducted a comparative analysis of neuronal activation repetitions and speed. Firstly, we calculated the number of repetitive activations of burst-related (or suppression-related) neuron across all burst (or suppression) events and counted the proportion of neurons involved in different numbers of burst events (Fig. 4i). Secondly, for each event, we computed the mean activation intensity of each activated neurons as the threshold, and then binarized the activation state of individual neuron at each timepoint, i.e., High-activation (firing intensity greater than the threshold) and Low-activation (firing intensity less than the threshold). The proportion of neurons in a high-activation state relative to the total number of activated neurons was calculated at each time point during individual burst or suppression events (Fig. 4j). A histogram of min-max normalized peak times, representing when the number of activated neurons reached its maximum, was generated, and distribution curves were fitted across four mice (Supplementary Fig. 6a). This approach revealed distinct temporal patterns in population activation between burst and suppression events, particularly in the speed of coordinated neuronal activation onset.

### Functional connectivity analysis of neural activities

#### Neuron-level functional connectivity

Based on the neuronal classification described above, neurons across the cerebral cortex were categorized into two distinct groups: burst-related and suppression-related neurons. To elucidate neuronal synchronization patterns across different brain states, we extracted representative calcium response traces from 25 neurons within each category (Fig. 4k, l). Functional connectivity at the single-neuron level was quantified by computing Pearson correlation coefficients between neuronal activity traces during awake, burst, and suppression states. Correlation matrices were constructed by averaging coefficients obtained from repeatedly activated neurons across all events (Fig. 4m). For consistency, awake-state events were uniformly defined as 20 frames in duration, whereas burst and suppression events were analyzed over their full temporal extent. The Two-sided Mann-Whitney U rank test revealed a significant increase in functional correlations during burst and suppression events compared with awake-state events.

#### Brain-region-level functional connectivity

To assess functional connectivity at the brain region scale, neuronal activities recorded via calcium imaging were averaged within each anatomically defined cortical region. Pearson correlations were then calculated pairwise between these regional activity profiles. Only correlations with p-values < 0.05 were retained for further analysis. Due to low neuron counts in regions RSPv and ACAd, these areas were excluded, focusing the analysis on 28 cortical regions. This yielded a 28×28 functional connectivity matrix and corresponding network graph with 28 nodes representing bilateral brain regions (Supplementary Fig. 6b, c). Network edges were weighted by correlation strength. For each of the four consciousness states (awake, burst, suppression, and recovery), twenty 1-second time windows were sampled, producing 20 functional connectivity matrices and network graphs per state. These were averaged to generate representative connectivity profiles for each state (Fig. 4n and Supplementary Fig. 6c). Network topology was further analyzed by examining the mean functional connection strength (Fig. 4o), node centrality metrics at the microscale, and average clustering coefficients at the macroscale to characterize state-dependent shifts in network organization (Supplementary Fig. 6d, e). The Two-sided Mann-Whitney U rank test (Fig. 4o and Supplementary Fig. 6e) and the Kolmogorov-Smirnov test (Supplementary Fig. 6d) are applied to detect statistically significant differences in these properties of functional connectivity networks.

#### Propagation analysis of electrical signals

We performed a detailed investigation of the burst waveforms recorded by 32-channel electrical signals, categorizing them into eight clusters based on spatial locations, ECoG waveforms, and dimensionality reduction results (Fig. 5a–c). The dimensionality reduction results were derived from linear discriminant analysis (LDA), applied to the multichannel electrical signals with a parameter setting of 3 (Fig. 5c). By aggregating and sorting the 32-channel electrical signals according to the defined clusters, we illustrated the propagation of neural activity characterized by multichannel electrical signals during a single burst suppression event (Fig. 5d and Supplementary Fig. 9a). To quantify the direction of this propagation process, we calculated the time points at which the peaks of the electrical signals appeared for each channel, as well as the distances of the electrode positions from the central vessel (Fig. 5e and Supplementary Fig. 9b).

### Cross-correlation analysis between neuronal and population signals

#### Neuron-level cross-correlation

To measure the correlation between neuronal activity and group activity, we computed the cross-correlation between calcium activity and electrical signals under various time-delay conditions. We selected neurons within a radius of 13 μm around each electrode location, and calculated the Pearson correlation between the selected neurons and the electrical signals for each burst event (Supplementary Fig. 10a). We aggregated the results across multiple sets of burst events during the anesthesia stage to create a correlation matrix between neural activity and electrical signals (Fig. 5f, left). In this analysis, we assessed the correlation between neuronal activity occurring early (Δtime < 0) or delayed (Δtime > 0) by *N* seconds relative to the electrical signal, using the electrical signal as the zero-reference point(Supplementary Fig. 10b). In addition, we averaged the data from each channel to obtain the cross-correlation at the channel level (Fig. 5f, right). To further investigate the differential mechanisms across distinct regions, we calculated the peak time of each neuron during individual burst events and computed the deviation between the peak time of neurons and the average peak time in each region, taking the absolute value. We then normalized these values over multiple channels to highlight differences. This allowed us to compare the fluctuation in neuronal peak time across different regions. The Two-sided Wilcoxon signed-rank test revealed significant differences in phase differences across different channels (Fig. 5g, bottom). For a more intuitive visualization of calcium activity in different regions, we displayed the average traces pattern of neurons in each cluster during the bursting event (Fig. 5g, top). In addition, the standard deviation of neuronal trace within each region was annotated.

#### Brain-region-level cross-correlation

In addition, we provided a brain heatmap illustrating the distribution of cross-correlations between ECoG and regional averaged neurons in physical space for earlier-than-onset, onset, and later-than-onset conditions, along with a comparison of correlation statistics for each channel (Supplementary Fig. 11a, b). We conducted cross-correlation analysis between single neuron activities obtained through calcium imaging and ECoG signals obtained through electrodes. From the 14 brain regions where both types of signals were recorded simultaneously, we randomly selected up to 100 neurons from each region, totaling 1228 neurons across these regions, along with 28 electrodes. We then computed the Pearson correlation between each neuron and each electrode and generated a cross-correlation matrix (Supplementary Fig. 11c). The values near the diagonal within the yellow box in the third panel depict the correlation coefficients between one or more electrodes and each neuron in the same brain regions, illustrating the coupling of a single neuron to the average population activity in its vicinity. The spatial distribution of these corresponding cross-correlations within brain regions is displayed at the bottom of Fig. 5c.

### Prediction of neuronal calcium dynamics from multi-channel ECoG signals during burst-suppression and awake states

#### Data dimensionality and alignment

Due to inherent differences in temporal and spatial resolution between ECoG and calcium imaging, their data dimensions are complementary yet mismatched. ECoG recordings consist of 32 channels sampled at 1 kHz, forming a matrix of size (T × 1000, 32), while calcium imaging covers ~ 17,655 neurons sampled at 10 Hz, resulting in a matrix of size (T × 10, 17655). To enable modeling, we aligned their temporal and feature dimensions via feature extraction and dimensionality reduction (Fig. 6a).

#### ECoG features extraction

The raw 32-channel ECoG data were segmented into windows of 100 consecutive time points. From each window, 18 statistical and spectral features were computed per channel, including mean, standard deviation, max, min, median, peak-to-peak range, interquartile range, energy, skewness, kurtosis, RMS, waveform factor, crest factor, zero crossings, dominant frequency, spectral centroid, spectral entropy, and bandwidth, resulting in a 576-dimensional feature vector per window (32 channels × 18 features). This transformed the data into a matrix of size (T × 10 × 576), better matching the calcium data’s temporal resolution. The extracted features were normalized prior to model training.

#### Calcium signal dimensionality reduction via shared variance component analysis (SVCA)

We utilized Shared Variance Component Analysis (SVCA)^46,49^ to reduce the dimensionality of calcium signals, assessing the extent to which neural population variance is reliably captured by low-dimensional latent dynamics. By applying maximum covariance analysis to subsets of the neuronal population with cross-validation over time, SVCA offered a robust framework for dimensionality reduction. To prepare the data, neurons were subsampled using strategies such as random selection, central expansion, or lateral field of view placement, followed by z-scoring their activities. Neuronal populations were divided into two sets using a checkerboard pattern to minimize crosstalk. Temporal segments were assigned as training or testing sets. Covariance matrices between neuron sets were decomposed using singular value decomposition to identify shared variance components (SVCs). The reliable variance for each SVC was then normalized against the total variance to determine its contribution percentage. To ensure reliability, an SVC was considered significant if its variance exceeded four standard deviations above the mean from shuffled datasets, which effectively removed temporal alignment. Neuronal populations were visualized by sequencing cells according to their correlation-based clustering patterns across the wide cortex, allowing identification of neurons with relatively weaker activity during the suppression phase. Simultaneous electrophysiological recordings provided clear temporal indicators of burst or suppression events.

#### Prediction of SVCs from ECoG features using reduced rank regression (RRR)

To predict shared variance components (SVCs) of neural activity from electrocorticography (ECoG) features, we employed reduced rank regression (RRR). This method allowed us to explore the relationship between high-dimensional neural data and behavioral states captured through ECoG. We began by identifying feasible ranks for regression based on the dimensionality of the extracted ECoG features. Various regularization parameters were tested to optimize model performance. For each parameter setting, canonical correlation analysis was used to find linear transformations that maximized the covariance between the neural projections and ECoG features. This resulted in predicted projections for both training and testing datasets. To refine these predictions, we applied a Savitzky-Golay filter, which smoothed the time series data without distorting its underlying structure. The filtered results were then centered to ensure zero mean, aiding in the accurate comparison of real versus predicted neural activity over specific time intervals. Visualization played a crucial role in evaluating model performance. We generated plots comparing the actual SVCs with their predicted counterparts across different experimental conditions. A range of model ranks was tested to determine the optimal predictive capacity, focusing on the most informative SVCs. For further validation, we fitted models using a rastermap approach^70^, optimizing parameters such as PCs and cluster count to best capture the variability in combined training and test sets. In addition, comparisons of model predictions under different chunked time segments ensured robustness against temporal variations. The RRR methodology enabled a detailed examination of how well ECoG-derived features could predict neural dynamics at multiple levels, offering insights into the underlying mechanisms of neural information processing.

#### Statistics and reproducibility

This section provides information on study design, statistical analyses, and experimental reproducibility. No statistical method was used to predetermine sample size. Sample sizes were chosen based on established practice in previous studies employing large-scale neuronal population imaging in mice. The numbers of animals, recording sessions, and analytical units used in each experiment are specified in the corresponding figure legends.

Statistical analyses were performed using Python (v3.10) and MATLAB (MathWorks). Data are presented as mean ± s.e.m. unless otherwise indicated. Data normality was assessed using the Shapiro–Wilk test before the application of parametric tests. For comparisons between two groups, two-sided paired or unpaired Student’s *t* tests were used when normality assumptions were satisfied; otherwise, nonparametric tests (Wilcoxon signed-rank test or Mann–Whitney U test) were applied. For comparisons involving more than two groups, one-way or repeated-measures analysis of variance was performed, followed by Tukey’s post hoc multiple-comparison test where appropriate. Correlation analyses were conducted using Pearson’s correlation coefficient unless otherwise specified. The statistical tests used for each analysis, including sidedness and any multiple-comparison adjustments, are specified in the corresponding figure legends. Statistical significance was defined as *P* < 0.05.

Data collection and analysis were not randomised because the experimental conditions were defined by controlled physiological state transitions, including anaesthesia induction and recovery. Blinding was not applied during data acquisition or analysis because experimental conditions were identifiable from the recorded physiological signals. No data were excluded from the analyses unless otherwise stated. Experimental reproducibility is described in the Methods, figure legends, and Reporting Summary.

### Reporting summary

Further information on research design is available in the Nature Portfolio Reporting Summary linked to this article.

## Supplementary information

Supplementary Information

Supplementary Video 1

Supplementary Video 2

Supplementary Video 3

Supplementary Video 4

Supplementary Video 5

Reporting Summary

Transparent Peer Review file

## Source data

Source Data

## Data Availability

The calcium imaging raw data supporting the findings of this study exceed 100 TB in size. To facilitate access, representative demo data used for the analyses are available at 10.5281/zenodo.18295118. All processed data related to each mouse, including extracted neuronal traces and electrophysiological signals, are available at 10.5281/zenodo.18324142. The raw dataset is available from the corresponding authors without restriction. Source data are provided in this paper.

## Code availability

All custom code used for data analysis is available at the following repositories: https://github.com/YangMoTsinghua/Predict_Calcium_From_ECoG, https://github.com/IrisRegion/Burst_suppression_analysis_based_on_cortex-wide_neural_optoelectronic_interface.git, and on Zenodo (10.5281/zenodo.18295118).

## References

[1] Shanker, A., Abel, J. H., Schamberg, G. & Brown, E. N. Etiology of Burst Suppression EEG Patterns. *Front. Psychol.* **12**, 673529 (2021). 34177731 10.3389/fpsyg.2021.673529 PMC8222661

[2] Amzica, F. What does burst suppression really mean? *Epilepsy Behav.* **49**, 234–237 (2015). 26195335 10.1016/j.yebeh.2015.06.012

[3] Brown, E. N., Lydic, R. & Schiff, N. D. General anesthesia, sleep, and coma. *N. Engl. J. Med.* **363**, 2638–2650 (2010). 21190458 10.1056/NEJMra0808281 PMC3162622

[4] Franks, N. P. General anaesthesia: from molecular targets to neuronal pathways of sleep and arousal. *Nat. Rev. Neurosci.* **9**, 370–386 (2008). 18425091 10.1038/nrn2372

[5] Alkire, M. T., Hudetz, A. G. & Tononi, G. Consciousness and Anesthesia. *Science* **322**, 5 (2008). 10.1126/science.1149213 PMC2743249 18988836

[6] Kroeger, D. & Amzica, F. Hypersensitivity of the anesthesia-induced comatose brain. *J. Neurosci.* **27**, 10597–10607 (2007). 17898231 10.1523/JNEUROSCI.3440-07.2007 PMC6673173

[7] Ching, S., Purdon, P. L., Vijayan, S., Kopell, N. J. & Brown, E. N. A neurophysiological-metabolic model for burst suppression. *Proc. Natl. Acad. Sci. USA* **109**, 3095–3100 (2012). 22323592 10.1073/pnas.1121461109 PMC3286963

[8] Vijn, P. C. & Sneyd, J. R. I.v. anaesthesia and EEG burst suppression in rats: bolus injections and closed-loop infusions. *Br. J. Anaesth.* **81**, 6 (1998). 9861133 10.1093/bja/81.3.415

[9] Sirmpilatze, N. et al. Spatial signatures of anesthesia-induced burst-suppression differ between primates and rodents.* Elife*** 11**, e74813 (2022). 10.7554/eLife.74813 PMC9129882 35607889

[10] Donald, L. & Clark, B. S. R. Neurophysiologic effects of general anesthetics. *Anesthesiology* **35**, 17 (1973). 4145825

[11] Krahe, R. & Gabbiani, F. Burst firing in sensory systems. *Nat. Rev. Neurosci.* **5**, 13–23 (2004). 14661065 10.1038/nrn1296

[12] Lewis, L. D. et al. Local cortical dynamics of burst suppression in the anaesthetized brain. *Brain* **136**, 2727–2737 (2013). 23887187 10.1093/brain/awt174 PMC3754454

[13] Muller, L., Chavane, F., Reynolds, J. & Sejnowski, T. J. Cortical travelling waves: mechanisms and computational principles. *Nat. Rev. Neurosci.* **19**, 255–268 (2018). 29563572 10.1038/nrn.2018.20 PMC5933075

[14] Park, D.-W. et al. Graphene-based carbon-layered electrode array technology for neural imaging and optogenetic applications.* Nat. Commun.* **5**, (2014). 10.1038/ncomms6258 10.1038/ncomms6258 PMC4218963 25327513

[15] Thunemann, M. et al. Deep 2-photon imaging and artifact-free optogenetics through transparent graphene microelectrode arrays.* Nature Communications*** 9**, 5258 (2018). 10.1038/s41467-018-04457-5 PMC5964174 29789548

[16] Park, D.-W. et al. Fabrication and utility of a transparent graphene neural electrode array for electrophysiology, in vivo imaging, and optogenetics. *Nat. Protoc.* **11**, 2201–2222 (2016). 27735935 10.1038/nprot.2016.127

[17] Driscoll, N. et al. Multimodal in vivo recording using transparent graphene microelectrodes illuminates spatiotemporal seizure dynamics at the microscale.* Commun. Biol.*** 4**, (2021). 10.1038/s42003-021-01670-9 10.1038/s42003-021-01670-9 PMC7846732 33514839

[18] Transparent arrays of bilayer-nanomesh microelectrodes for simultaneous electrophysiology and two-photon imaging in the brain.* Sci. Adv.*** 4**, 136 (2018–09–07). 10.1126/sciadv.aat0626 PMC6124910 30191176

[19] Seo, J. W. et al. Artifact-Free 2D mapping of neural activity in vivo through transparent gold nanonetwork array.* Adv. Funct. Mater.*** 30**, 2000896 (2020).

[20] Zhang, J. et al. Stretchable transparent electrode arrays for simultaneous electrical and optical interrogation of neural circuits in vivo. *Nano Lett.* **18**, 2903–2911 (2018). 29608857 10.1021/acs.nanolett.8b00087

[21] Wang, X. et al. Subdural neural interfaces for long-term electrical recording, optical microscopy and magnetic resonance imaging.* Biomaterials*** 281**, 121352 (2022). 10.1016/j.biomaterials.2021.121352 34995902

[22] Nishimura, A. et al. Totally transparent hydrogel-based subdural electrode with patterned salt bridge.* Biomed. Microdev.*** 22**, 10.1007/s10544-020-00517-0 (2020). 10.1007/s10544-020-00517-0 32827271

[23] Dijk, G., Kaszas, A., Pas, J. & O’Connor, R. P. Fabrication and in vivo 2-photon microscopy validation of transparent PEDOT:PSS microelectrode arrays.* Microsyst Nanoeng*** 8**, 90 (2022). 10.1038/s41378-022-00434-7 PMC9424218 36051746

[24] Cho, Y. U. et al. Ultra-low cost, facile fabrication of transparent neural electrode array for electrocorticography with photoelectric artifact-free optogenetics.* Adv. Funct. Materi.*** 32**, 2105568 (2021).

[25] Donaldson, P. D. et al. Polymer skulls with integrated transparent electrode arrays for cortex-wide opto-electrophysiological recordings.* Adv. Healthcare Mater.*** 11**, e2200626 (2022). 10.1002/adhm.202200626 PMC9573805 35869830

[26] Liu, X. et al. E-Cannula reveals anatomical diversity in sharp-wave ripples as a driver for the recruitment of distinct hippocampal assemblies.* Cell Rep.*** 41**, 10.1016/j.celrep.2022.111453 (2022). 10.1016/j.celrep.2022.111453 PMC9640218 36198271

[27] Ghanbari, L. et al. Cortex-wide neural interfacing via transparent polymer skulls.* Nat. Commun.*** 10**, 1500 (2019). 10.1038/s41467-019-09488-0 PMC6445105 30940809

[28] Ramezani, M. et al. High-density transparent graphene arrays for predicting cellular calcium activity at depth from surface potential recordings. *Nat. Nanotechnol.* **19**, 504–513 (2024). 38212523 10.1038/s41565-023-01576-z PMC11742260

[29] Rupprecht, P. et al. A database and deep learning toolbox for noise-optimized, generalized spike inference from calcium imaging. *Nat. Neurosci.* **24**, 1324–1337 (2021). 34341584 10.1038/s41593-021-00895-5 PMC7611618

[30] Theis, L. et al. Benchmarking spike rate inference in population calcium imaging. *Neuron* **90**, 471–482 (2016). 27151639 10.1016/j.neuron.2016.04.014 PMC4888799

[31] Kim, T. H. & Schnitzer, M. J. Fluorescence imaging of large-scale neural ensemble dynamics. *Cell* **185**, 9–41 (2022). 34995519 10.1016/j.cell.2021.12.007 PMC8849612

[32] Fan, J. et al. Video-rate imaging of biological dynamics at centimetre scale and micrometre resolution. *Nat. Photonics* **13**, 809–816 (2019).

[33] Allen, W. E. et al. Thirst regulates motivated behavior through modulation of brainwide neural population dynamics. *Science* **364**, 253 (2019). 30948440 10.1126/science.aav3932 PMC6711472

[34] Xiao, G. et al. Microelectrode arrays modified with nanocomposites for monitoring dopamine and spike firings under deep brain stimulation in rat models of Parkinson’s disease. *Acs Sens.* **4**, 1992–2000 (2019). 31272150 10.1021/acssensors.9b00182

[35] Xiao, G. et al. In situ detection of neurotransmitters and epileptiform electrophysiology activity in awake mice brains using a nanocomposites modified microelectrode array. *Sens. Actuators B Chem.* **288**, 601–610 (2019).

[36] Harris, J. A. et al. Anatomical characterization of Cre driver mice for neural circuit mapping and manipulation.* Front. Neural Circuits*** 8**, 10.3389/fncir.2014.00076 (2014). 10.3389/fncir.2014.00076 PMC4091307 25071457

[37] Zhang, Y. et al. Rapid detection of neurons in widefield calcium imaging datasets after training with synthetic data.* Nat. Methods*** 20**, 747–754 (2023). 10.1038/s41592-023-01838-7 PMC10172132 37002377

[38] Zhou, P. et al. Efficient and accurate extraction of in vivo calcium signals from microendoscopic video data.* Elife*** 7**, 10.7554/elife.28728 (2018). 10.7554/eLife.28728 PMC5871355 29469809

[39] Ward, M. P., Rajdev, P., Ellison, C. & Irazoqui, P. P. Toward a comparison of microelectrodes for acute and chronic recordings. *Brain Res.* **1282**, 183–200 (2009). 19486899 10.1016/j.brainres.2009.05.052

[40] Zhao, S. et al. Full activation pattern mapping by simultaneous deep brain stimulation and fMRI with graphene fiber electrodes.* Nat. Commun.*** 11**, 1788 (2020). 10.1038/s41467-020-15570-9 PMC7156737 32286290

[41] Zhao, Z. et al. Two-photon synthetic aperture microscopy for minimally invasive fast 3D imaging of native subcellular behaviors in deep tissue. *Cell* **186**, 2475–2491 (2023). 37178688 10.1016/j.cell.2023.04.016

[42] Raklc, P.S.G. et al. Cellular basis of working memory.* Neuron*** 14**, 477–485 (1995). 10.1016/0896-6273(95)90304-6 7695894

[43] Miller, E. K. An integrative theory of prefrontal cortex function.* Annu. Rev. Neurosci.*** 24**, 167–202 (2001). 10.1146/annurev.neuro.24.1.167 11283309

[44] Li, B. et al. Circuit mechanism for suppression of frontal cortical ignition during NREM sleep. *Cell* **186**, 5739–5750 (2023). 38070510 10.1016/j.cell.2023.11.012

[45] Dong, Y., Li, J., Zhou, M., Du, Y. & Liu, D. Cortical regulation of two-stage rapid eye movement sleep. *Nat. Neurosci.* **25**, 1675–1682 (2022). 36396977 10.1038/s41593-022-01195-2

[46] Stringer, C. et al. Spontaneous behaviors drive multidimensional, brainwide activity. *Science* **364**, 255 (2019). 31000656 10.1126/science.aav7893 PMC6525101

[47] Elsayed, G. F., Lara, A. H., Kaufman, M. T., Churchland, M. M. & Cunningham, J. P. Reorganization between preparatory and movement population responses in motor cortex. *Nat. Commun.* **7**, 13239 (2016). 27807345 10.1038/ncomms13239 PMC5095296

[48] Gallego, J. A., Perich, M. G., Miller, L. E. & Solla, S. A. Neural manifolds for the control of movement. *Neuron* **94**, 978–984 (2017). 28595054 10.1016/j.neuron.2017.05.025 PMC6122849

[49] Manley, J. et al. Simultaneous, cortex-wide dynamics of up to 1 million neurons reveal unbounded scaling of dimensionality with neuron number. *Neuron* **112**, 1694–1709 e1695 (2024). 38452763 10.1016/j.neuron.2024.02.011 PMC11098699

[50] Adam, E., Kwon, O., Montejo, K. A. & Brown, E. N. Modulatory dynamics mark the transition between anesthetic states of unconsciousness. *Proc. Natl. Acad. Sci. USA* **120**, e2300058120 (2023). 37467269 10.1073/pnas.2300058120 PMC10372635

[51] Iqbal, F. et al. Anesthetics: from modes of action to unconsciousness and neurotoxicity. *J. Neurophysiol.* **122**, 760–787 (2019). 31242059 10.1152/jn.00210.2019

[52] Curic, D., Ashby, D. M., McGirr, A. & Davidsen, J. Existence of multiple transitions of the critical state due to anesthetics. *Nat. Commun.* **15**, 7025 (2024). 39147749 10.1038/s41467-024-51399-2 PMC11327335

[53] Tort-Colet, N., Capone, C., Sanchez-Vives, M. V. & Mattia, M. Attractor competition enriches cortical dynamics during awakening from anesthesia. *Cell Rep.* **35**, 109270 (2021). 34161772 10.1016/j.celrep.2021.109270

[54] Palopoli-Trojani, K. et al. High-density cortical microECoG arrays concurrently track spreading depolarizations and long-term evolution of stroke in awake rats. *Commun. Biol.* **7**, 263 (2024). 38438529 10.1038/s42003-024-05932-0 PMC10912118

[55] Davis, Z. W., Muller, L., Martinez-Trujillo, J., Sejnowski, T. & Reynolds, J. H. Spontaneous travelling cortical waves gate perception in behaving primates. *Nature* **587**, 432–436 (2020). 33029013 10.1038/s41586-020-2802-y PMC7677221

[56] Lee, J. M. et al. The ultra-thin, minimally invasive surface electrode array NeuroWeb for probing neural activity.* Nat. Commun.*** 14**, 7088 (2023). 10.1038/s41467-023-42860-9 PMC10625630 37925553

[57] Le Floch, P. et al. 3D spatiotemporally scalable in vivo neural probes based on fluorinated elastomers. *Nat. Nanotechnol.* **19**, 319–329 (2023). 38135719 10.1038/s41565-023-01545-6

[58] Zhang, F. et al. Multimodal electrocorticogram active electrode array based on zinc oxide-thin film transistors.* Adv. Sci.*** 10**, e2204467 (2022). 10.1002/advs.202204467 PMC9839861 36403238

[59] Sun, F. et al. A genetically encoded fluorescent sensor enables rapid and specific detection of dopamine in flies, fish, and mice. *Cell* **174**, 481–496 (2018). 30007419 10.1016/j.cell.2018.06.042 PMC6092020

[60] Pittolo, S. et al. Dopamine activates astrocytes in prefrontal cortex via α1-adrenergic receptors.* Cell Rep.*** 40**, 111426 (2022). 10.1016/j.celrep.2022.111426 PMC9555850 36170823

[61] Feng, J. et al. A genetically encoded fluorescent sensor for rapid and specific in vivo detection of norepinephrine. *Neuron* **102**, 745–761 (2019). 30922875 10.1016/j.neuron.2019.02.037 PMC6533151

[62] Jing, M. et al. A genetically encoded fluorescent acetylcholine indicator for in vitro and in vivo studies.* Nat. Biotechnol.*** 36**, 726–737 (2018). 10.1038/nbt.4184 PMC6093211 29985477

[63] Xie, H. et al. Multifocal fluorescence video-rate imaging of centimetre-wide arbitrarily shaped brain surfaces at micrometric resolution.* Nat. Biomed. Eng.*** 8**, 740–753 (2023). 10.1038/s41551-023-01155-6 PMC11250366 38057428

[64] Huang, J. Y. et al. Intra-somatosensory cortical circuits mediating pain-induced analgesia. *Nat. Commun.* **16**, 1859 (2025). 39984470 10.1038/s41467-025-57050-y PMC11845469

[65] Li, J. et al. Annealing effect on indium tin oxide thin films by DC magnetron sputtering for alkali vapor cell heating.* Mod. Phys. Lett. B*** 33**, 10.1142/S0217984919501781 (2019).

[66] Li, J.* Indium Tin Oxide Thin Films Prepared by DC Magnetron Sputtering for Transparent Heating.* (2019).

[67] Middya, S. et al. Microelectrode arrays for simultaneous electrophysiology and advanced optical microscopy.* Adv. Sci.*** 8**, 2004434 (2021). 10.1002/advs.202004434 PMC9539726 36246164

[68] Zhang, F. et al. Multimodal electrocorticogram active electrode array based on zinc oxide-thin film transistors. *Adv. Sci.* **10**, e2204467 (2023). 10.1002/advs.202204467 PMC9839861 36403238

[69] Vogelstein, J., Friedrich, J., Zhou, P. & Paninski, L. Fast online deconvolution of calcium imaging data.* PLOS Comput. Biol.*** 13**, e1005423 (2017). 10.1371/journal.pcbi.1005423 PMC5370160 28291787

[70] Stringer, C. et al. Rastermap: a discovery method for neural population recordings. *Nat. Neurosci.* **28**, 201–212 (2025). 39414974 10.1038/s41593-024-01783-4 PMC11706777

