# Supplementary material for: Dynamic neuronal ensembles encode burst-suppression revealed by cortex-wide optical-electrical interfaces
Source: Nat Commun. 2026 Apr 29;17:5872. doi: 10.1038/s41467-026-72454-0 (PMC13333928; doi:10.1038/s41467-026-72454-0)
Supplement: Supplementary file 1 — Supplementary Information [file 41467_2026_72454_MOESM1_ESM.pdf]

Supplementary Information

Dynamic neuronal ensembles encode burst-suppression revealed by cortex-wide optical-electrical interfaces

Guihua Xiao, Mo Yang, Lingbo Li, Xintong Yao, Jingyu Xie, Xinyue Wang, Jinyu Zang, Yan Zhao, Tianyi Fang, Shuying Wu, Wandi Qi, Shipeng Lin, Wenxi Sun, Ting Lei, Bo Hong, Jiamin Wu, Qionghai Dai, Xiaochuan Dai

Supplementary Figures

**Supplementary Figure 1**	Detailed fabrication and characterization of the neural interface for *in vivo* recording
**Supplementary Figure 2**	Light-induced artifacts in electrophysiological recordings
**Supplementary Figure 3**	Power spectrum and corresponding ECoG waveforms during light (top), moderate (middle), and deep (bottom) anesthesia stages
**Supplementary Figure 4**	Normalized and sorted calcium neuronal rasterplot during burst and suppression.
**Supplementary Figure 5**	Normalized and sorted calcium neuronal raster plots during burst and suppression states
**Supplementary Figure 6**	Two groups of neurons firing as the burst and suppression stages across multiple mice
**Supplementary Figure 7**	The spatial distribution of responsive burst-related neurons and suppression-related neurons in the burst and suppression events.
**Supplementary Figure 8**	Analysis of functional connectivity at regional level across different stages of consciousness
**Supplementary Figure 9**	Burst propagation from sensory cortex to motor cortex across multiple mice.
**Supplementary Figure 10**	Cross-correlation of electrode and adjacent neurons
**Supplementary Figure 11**	ECoG-neuron cross-correlation analysis during different stages of consciousness across multiple mice
**Supplementary Figure 12**	Characterization of rapidly propagating spatiotemporal patterns and neuronal recruitment during a burst event

**Supplementary Figure 13**	Schematic illustration of Shared Variance Component Analysis (SVCA)
**Supplementary Figure 14**	Prediction of neuronal calcium activity from multi-channel ECoG signals
**Supplementary Figure 15**	ECoG-guided localization of labeled neurons in Thy1-YFP mouse brain sections

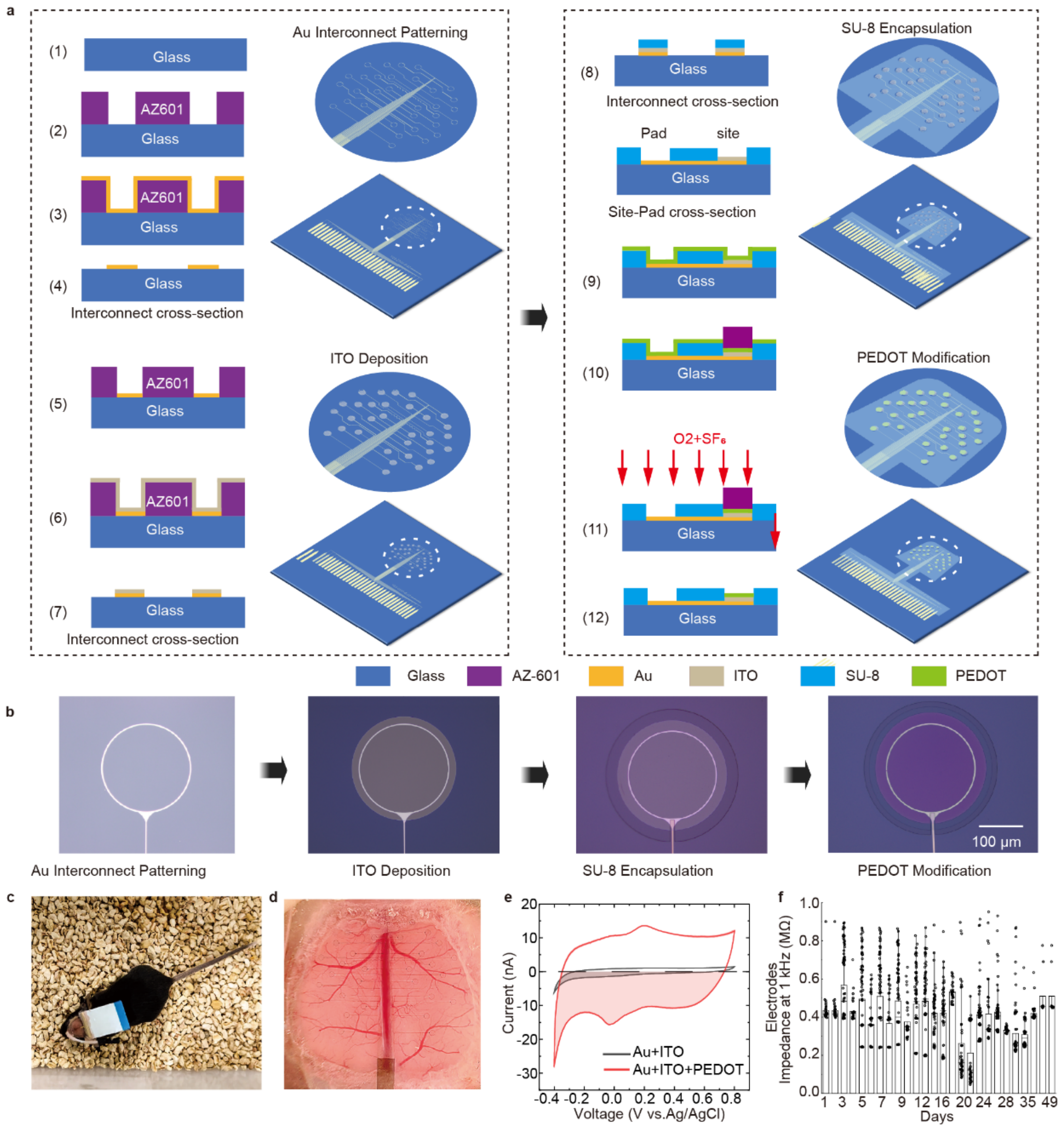

Supplementary Fig.1 | Detailed fabrication and characterization of the neural interface for *in vivo* recording. a. Detailed fabrication process of the neural interface, including patterning of gold interconnects, deposition of ITO, encapsulation with SU-8, and modification with PEDOT. **b**. Top view of each electrode at different stages of the fabrication process. **c**. Image of a live mouse post-surgery with the neural interface implantation. **d.** The exposed cortex with clear blood vessels beneath the transparent neural interface glass. **e**. Cyclic voltammetry curve of a typical electrode before and after PEDOT modification. **f**. Time-dependent impedance tracking for all electrodes in three implanted ECoG arrays (n=3 implanted arrays). Error bars: SEM.

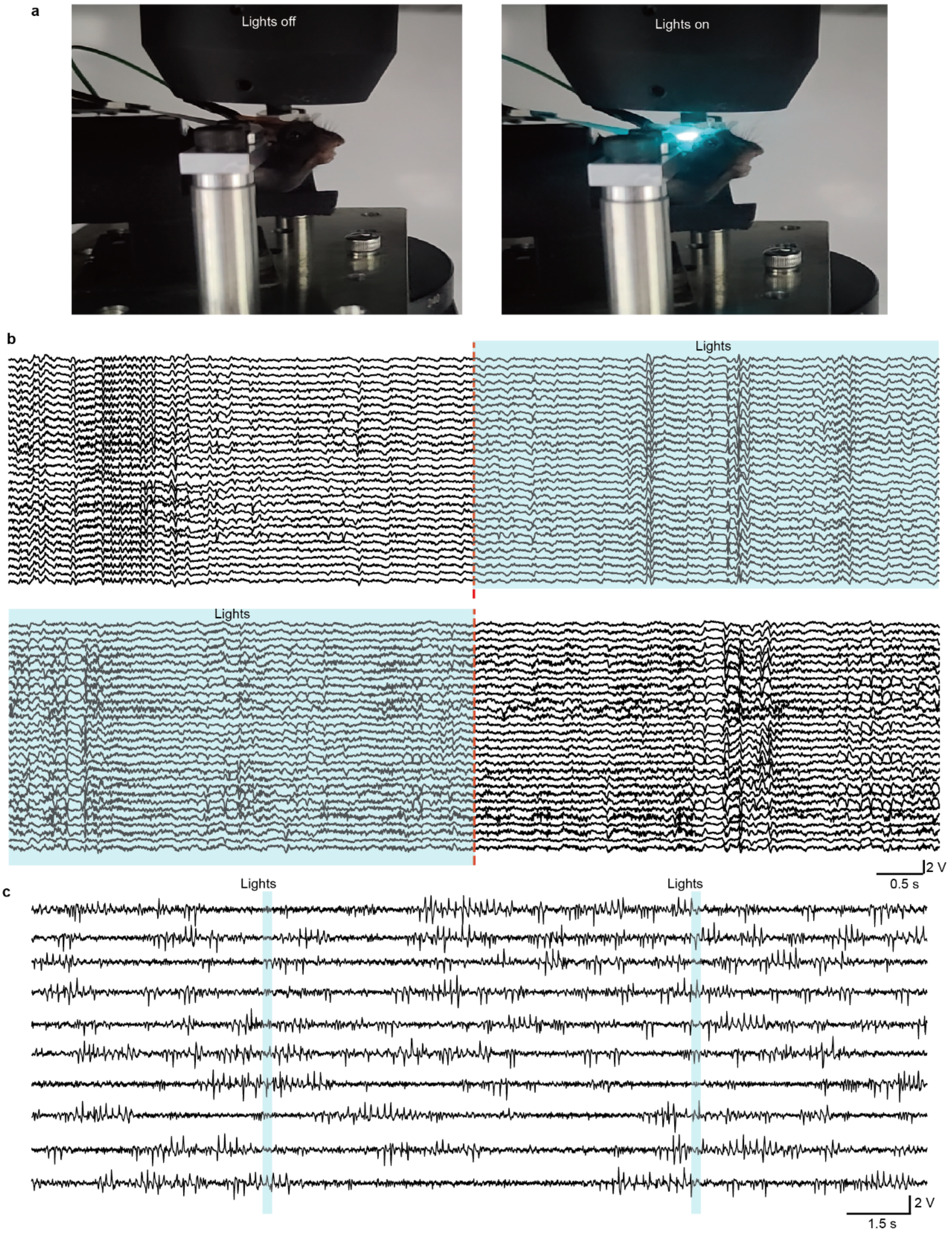

Supplementary Fig.2 | Light-induced artifacts in electrophysiological recordings. a. Images of the mouse brain under the microscope with the illumination off (left) and on (right). **b.** Electrophysiological recordings acquired during continuous illumination used for calcium imaging. **c.** Electrophysiological recordings acquired during pulsed illumination used for calcium imaging.

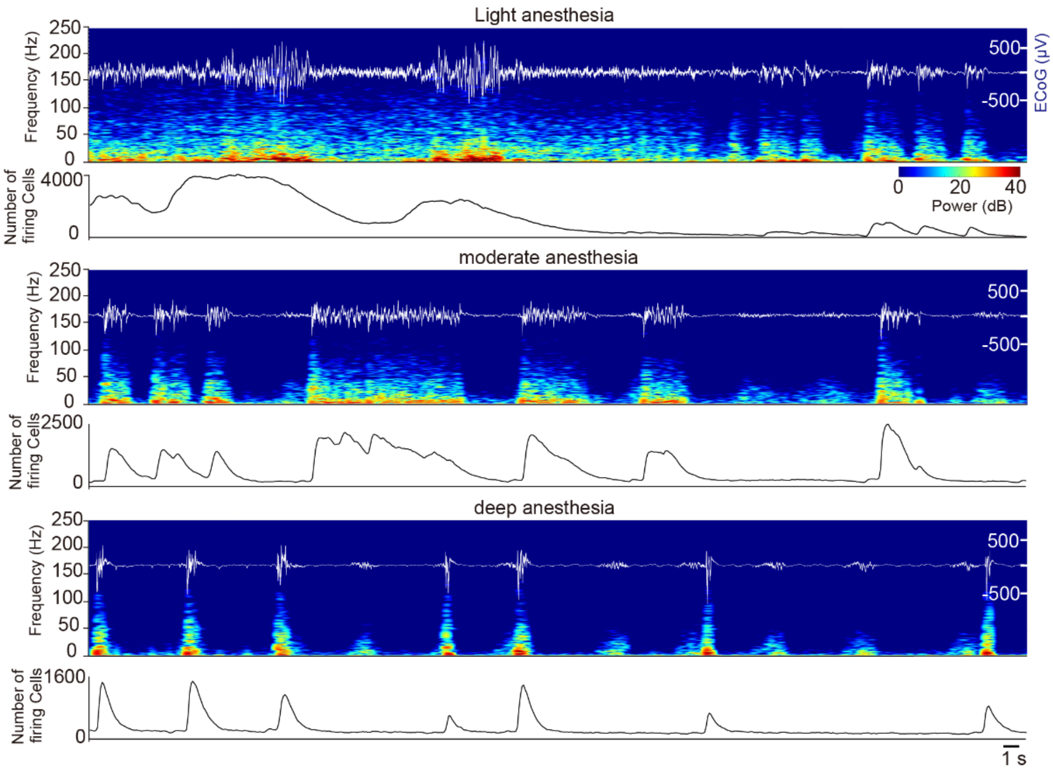

Supplementary Fig.3 | Power spectrum and corresponding ECoG waveforms during light (top), moderate (middle), and deep (bottom) anesthesia stages.

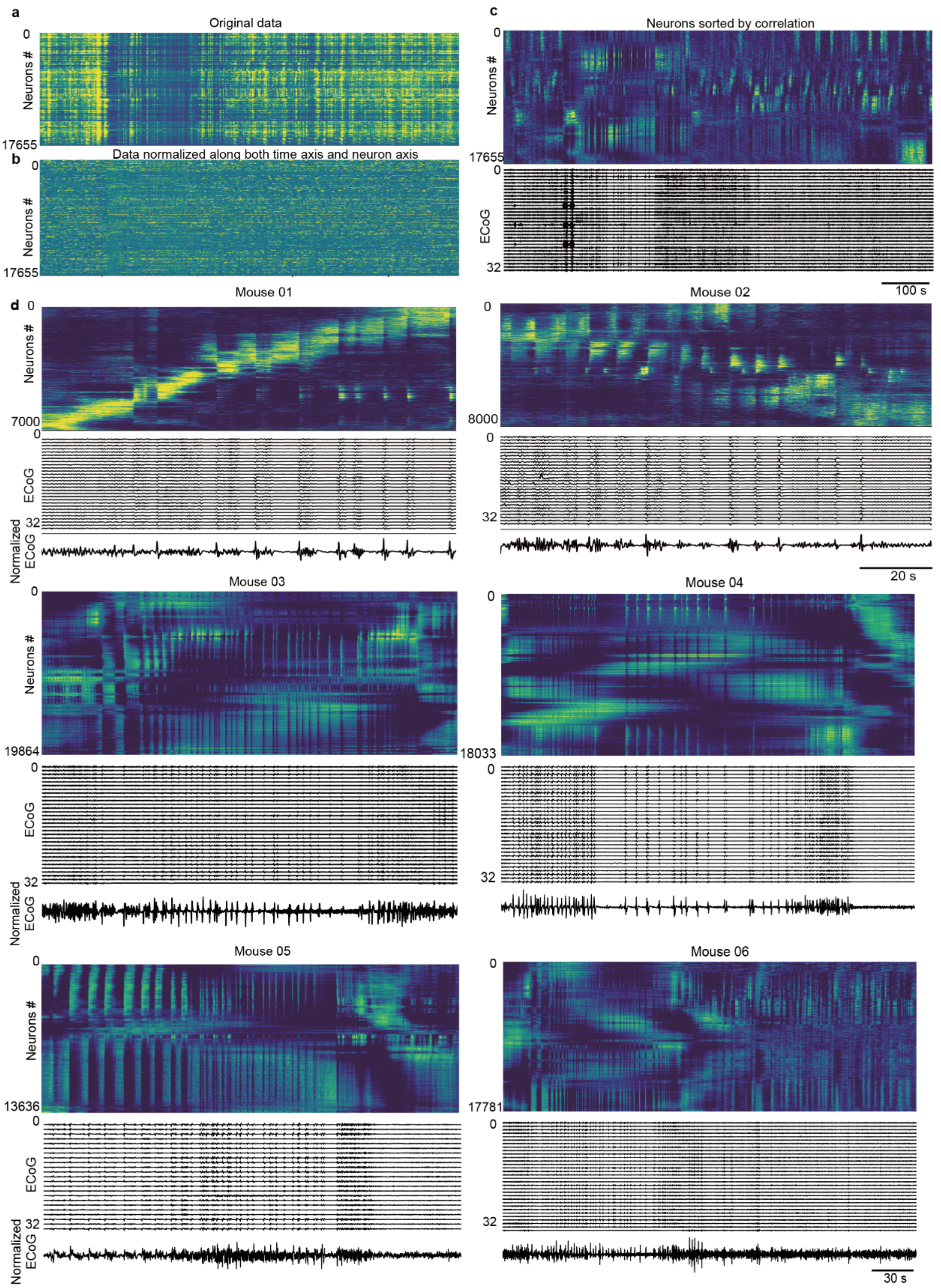

Supplementary Fig.4 | Normalized and sorted calcium neuronal rasterplot during burst and suppression. **a**. Original neuronal calcium raster plot. **b**. Normalized neuronal raster plot across both time axis and neuron axis (see Methods). **c.** The neuron raster plot sorted by correlation (top) and the corresponding ECoG signals (bottom) are presented across the whole recording session. **d**. Neuronal raster plots (top) and the corresponding ECoG signals (bottom) during burst and suppression periods across multiple mice.

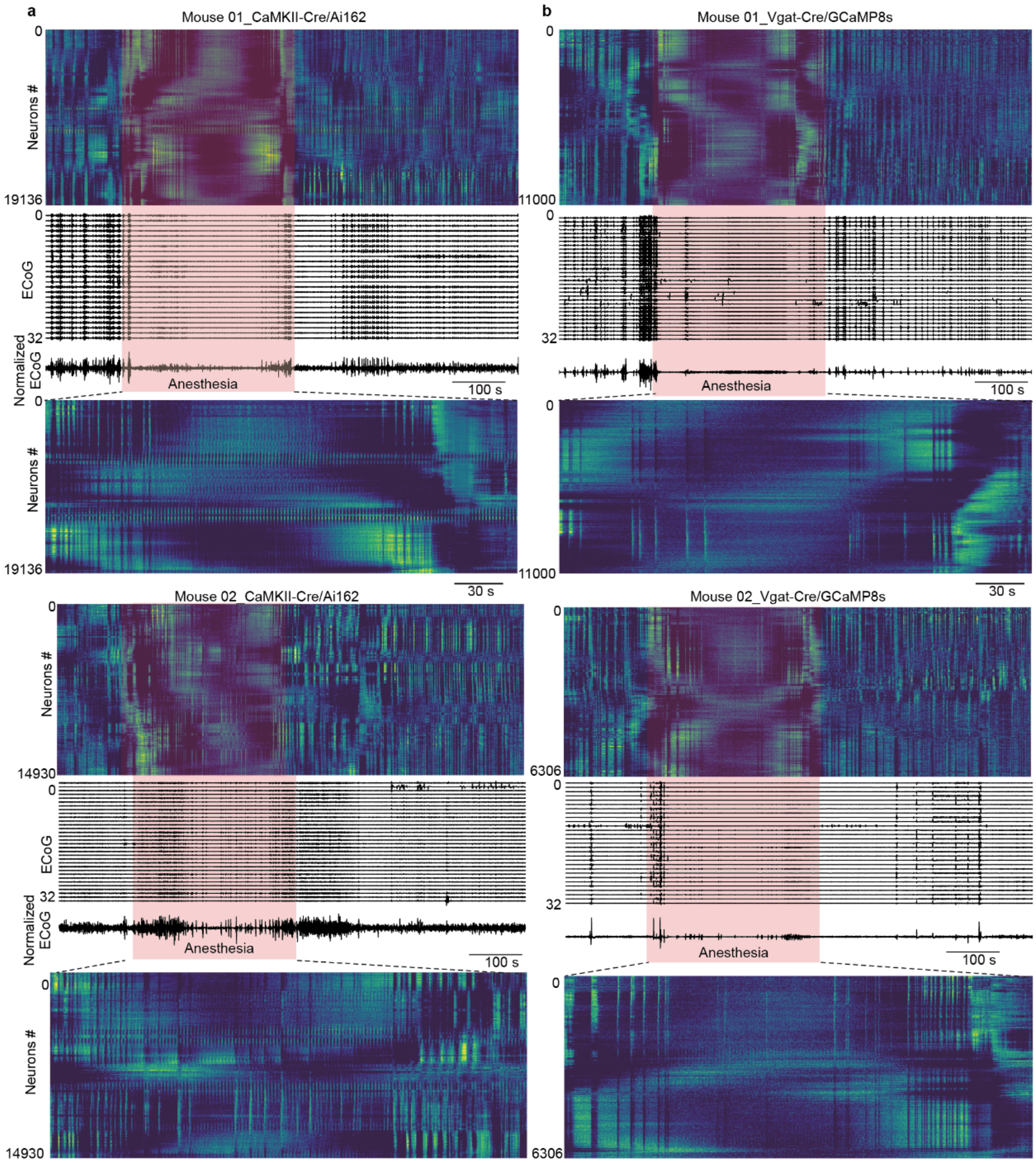

Supplementary Fig.5 | Normalized and sorted calcium neuronal raster plots during burst and suppression states, aligned with corresponding ECoG signals, in transgenic mice expressing GCaMP specifically in excitatory (CaMKII-Cre/Ai162d) (**a**) or inhibitory (Vgat-Cre/GCaMP8s) (**b**) neurons. Each panel includes the neuronal raster plot, the simultaneously recorded raw ECoG trace, a representative ECoG signal from the entire recording session, and enlarged views of neuronal activity during anesthesia.

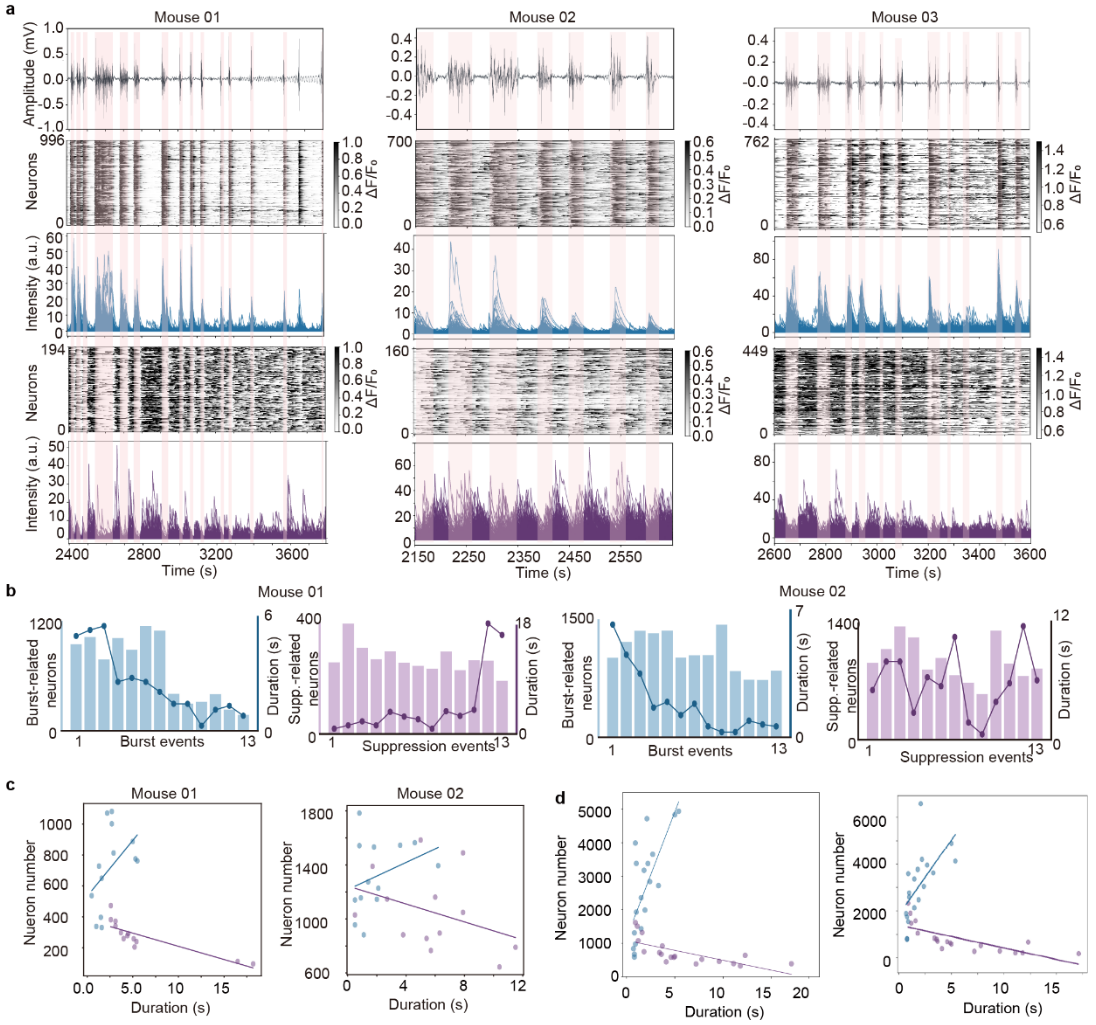

Supplementary Fig.6 | Two groups of neurons firing as the burst and suppression stages across multiple mice. a. Firing patterns of burst neurons and suppression neurons during the burst and suppression events, shown separately for three different mice. **b.** Neuron number and duration changes in the dynamic burst and suppression events across different mice. **c**, The relationship of neuron number and corresponding waveform durations across different mice. **d**. Comparison of neuron number and waveform duration when the comparison data window is 10 frames (left) and all burst frames (right). Correlation analyses were conducted using Pearson's correlation coefficient. Circles represent individual burst or suppression events.

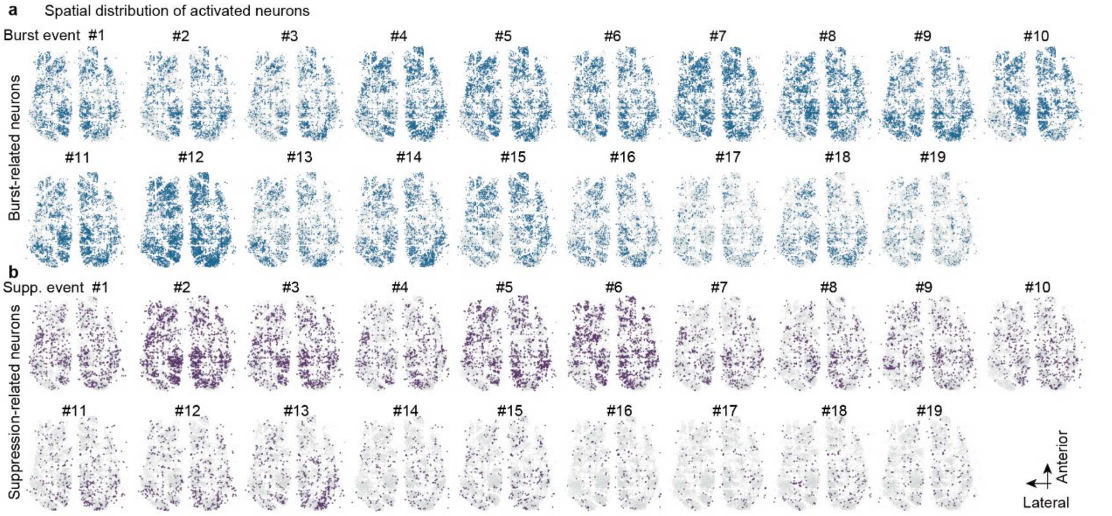

Supplementary Fig.7 | The spatial distribution of responsive burst-related neurons (a) and suppression-related (b) neurons in the burst and suppression events. **a.** Spatial distribution of activated neurons in burst events. Blue circles, activated neurons. Grey circles, inactive neurons. **b.** Spatial distribution of activated neurons in suppression events. Purple circles, activated neurons. Grey circles, inactive neurons.

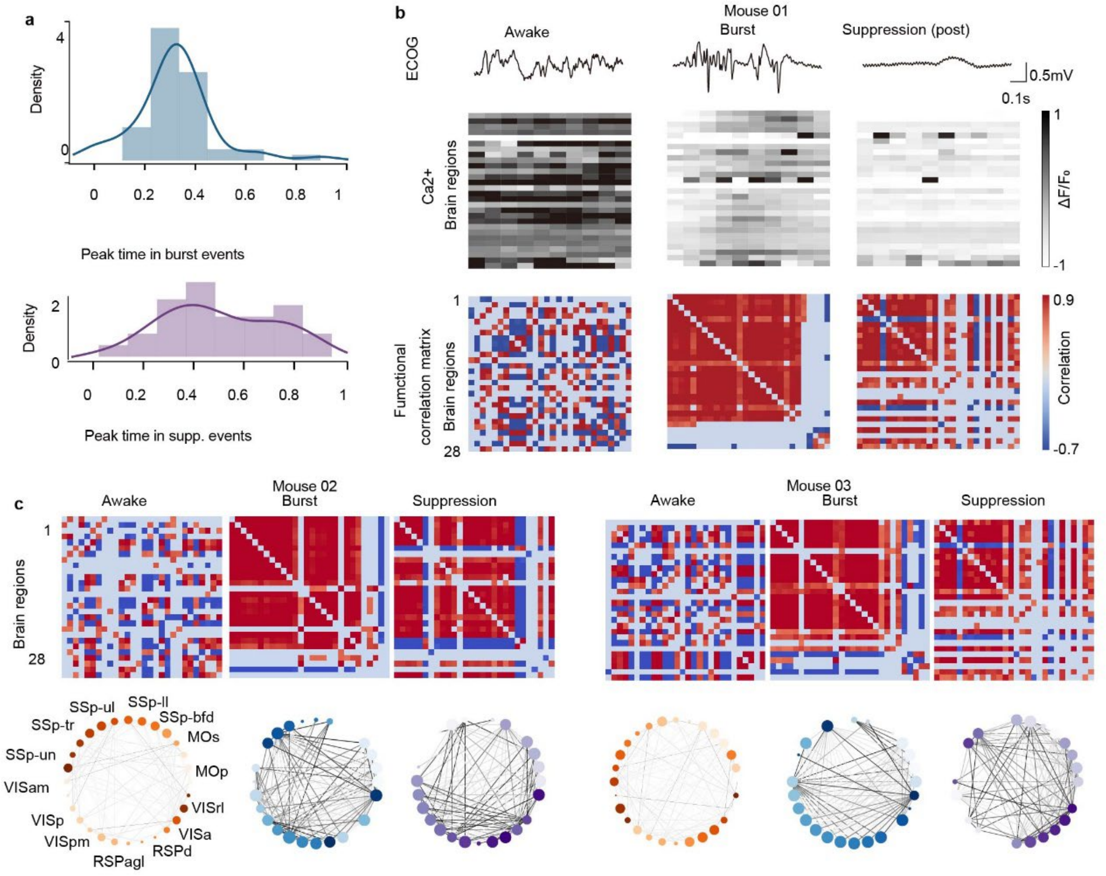

Supplementary Fig.8 | Analysis of functional connectivity at regional level across different stages of consciousness. a. The distribution of peak time of responsive neuron number within each burst or suppression across 4 mice. **b**, Each layer from top to bottom illustrates the representative ECoG waveform, the neural activities of 28 brain regions, the functional correlation matrices, respectively, from one typical mouse. **c,** The functional correlation matrices and the functional connectivity networks of neural activities from another two mice. The coordinates of functional correlation matrices correspond to the nodes of functional connectivity networks, representing 28 brain regions throughout the brain.

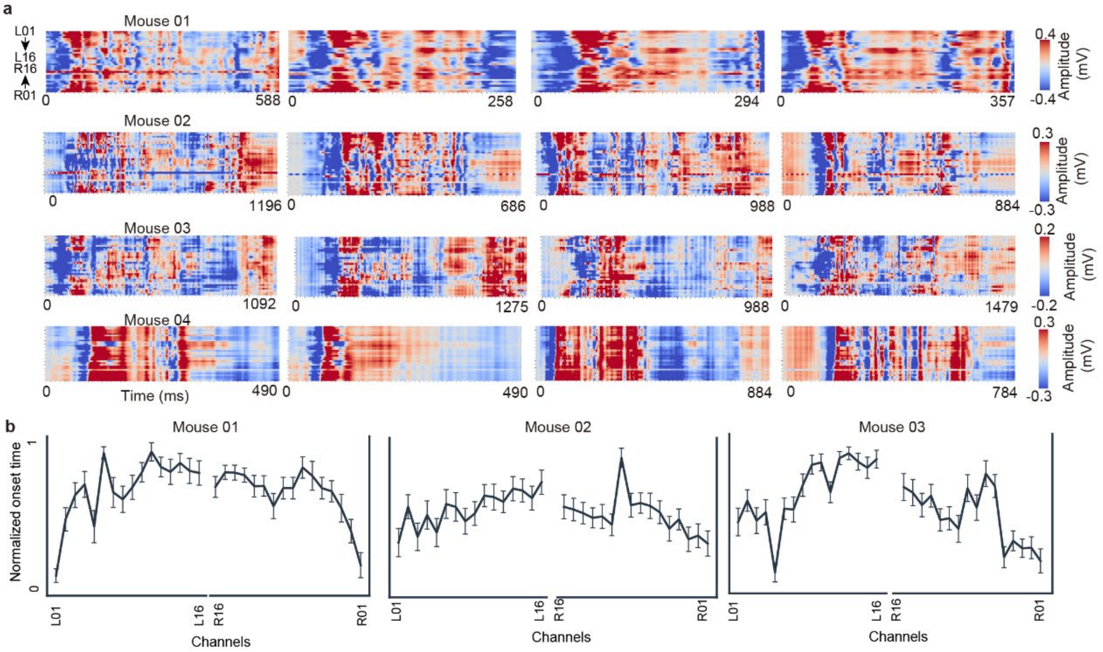

Supplementary Fig.9 | Burst propagation from sensory cortex to motor cortex across multiple mice. a. Heatmap of typical burst patterns from multiple mice. **b**. Statistical analysis of whole burst onset times across anesthesia states in three additional mice. Error bars: SEM.

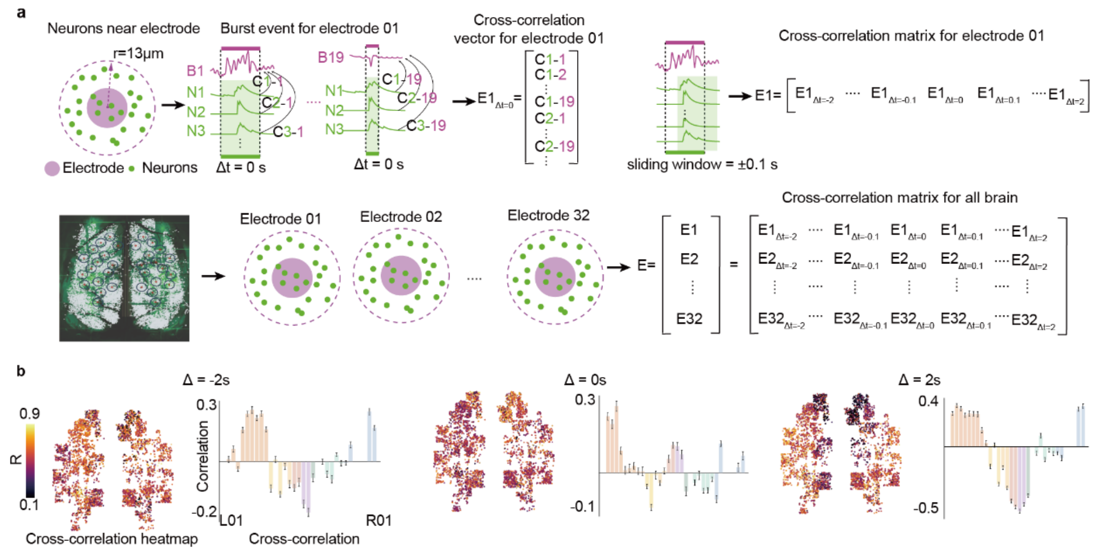

Supplementary Fig.10 | Cross-correlation of electrode and adjacent neurons. a. The calculation of cross-correlation between ECoG and individual neuronal activities, by grouping neurons within a 13 μm radius from each electrode site and calculating cross-correlation within different sliding window. Distribution of ECoG electrodes and single neurons on the dorsal cortex, with circles indicating neurons neighboring the corresponding electrodes. **b**. Cross-correlation heatmap onto the Allen Brain Atlas with phase differences of -2, 0, and +2 seconds, showing a distinct low–high–low transition in coupling strength around electrodes L-15/16 and R-15/16. Data are presented as mean ± SEM.

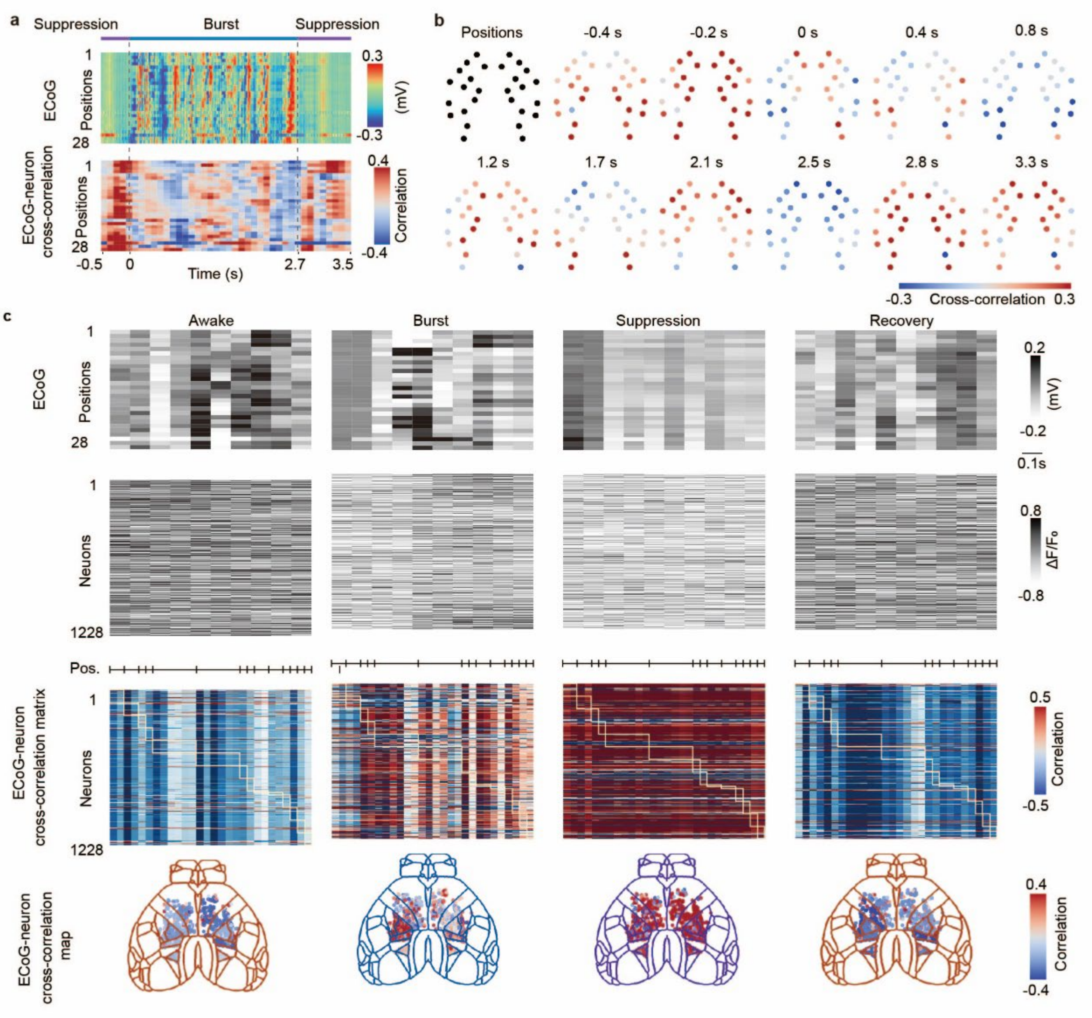

Supplementary Fig.11 | ECoG-neuron cross-correlation analysis during different stages of consciousness across multiple mice. a. The corresponding typical ECoG burst signals (top), and the average cross-correlation between each electrode and the neuronal activities of its corresponding brain region (bottom). **b.** Spatial locations ECoG electrodes, and the spatial distribution of cross-correlations corresponding to each time slice extracted during the time period consistent with (**a**). **c.** The first two layers from top to bottom are the ECoG signals, and the activities of 1228 neurons randomly selected from the whole brain, cross-correlation matrix between neuronal activities and ECoG signals, and the spatial locations of the cross-correlation between each neuron and the corresponding ECoG signals, align with the values on the approximate diagonal positions marked by the yellow boxes in the third layer.

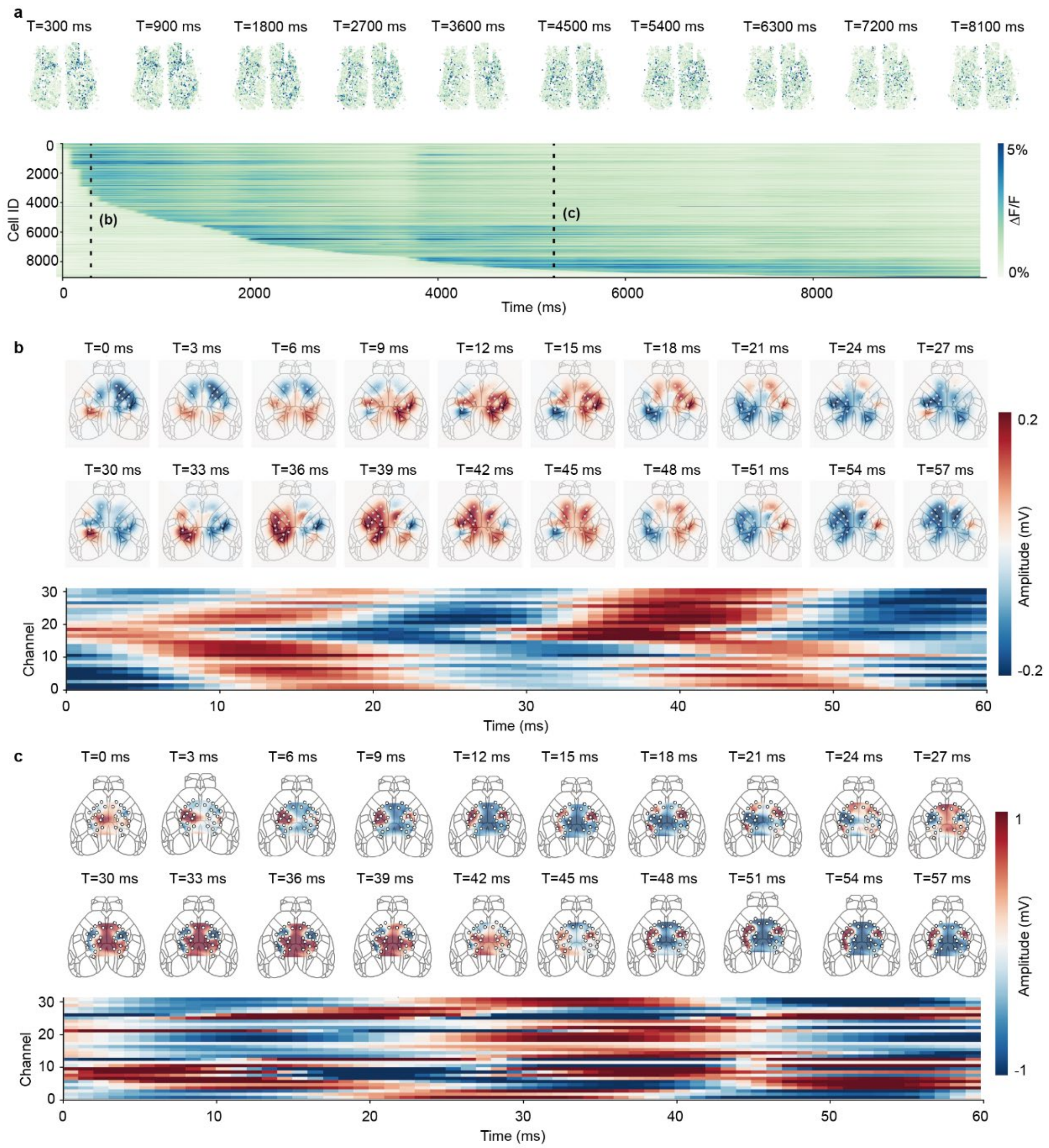

Supplementary Fig.12 | Characterization of rapidly propagating spatiotemporal patterns and neuronal recruitment during a burst event. a, Single-cell–level cortical dynamics characterized using calcium imaging. Neurons are arranged according to their activation onset time (bottom). **b–c,** Dynamics of ECoG signals within a 60-ms window corresponding to the period marked in **a**, shown at burst onset (**b**) and during the burst (**c**). The upper panels visualize 20 snapshots at the latencies indicated above.

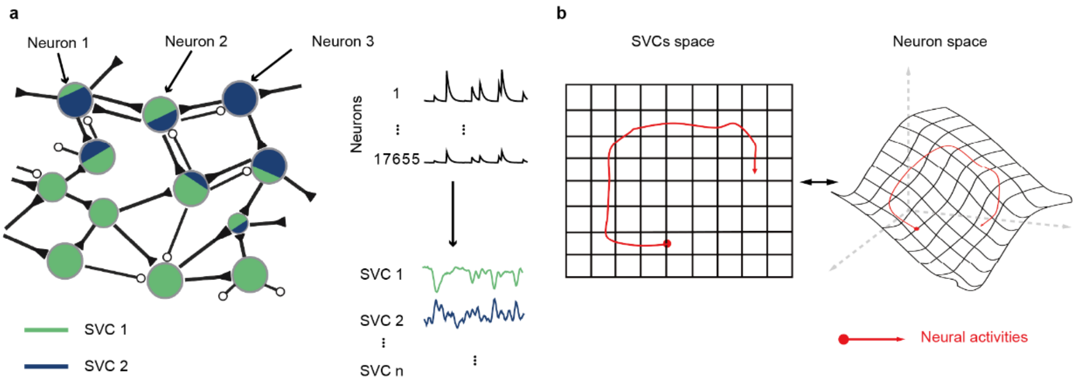

Supplementary Fig.13. Schematic illustration of Shared Variance Component Analysis (SVCA). a. SVCs serve as shared low-dimensional manifolds that capture the dominant population dynamics. **b**. The SVCA algorithm is invertible: while SVCA serves as a dimension reduction technique, neuronal activity can also be reconstructed from the SVCs back into neuron space.

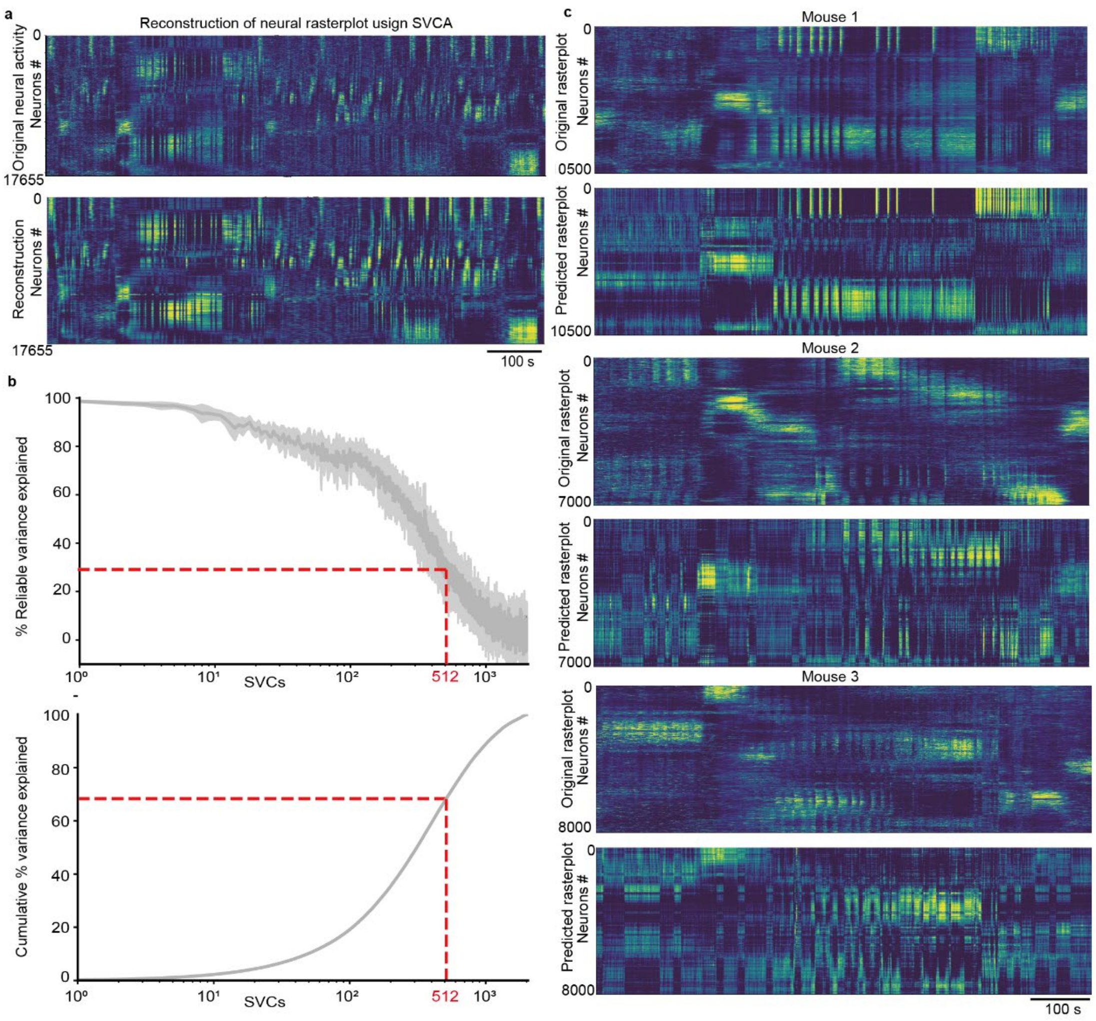

Supplementary Fig.14 | Prediction of neuronal calcium activity from multi-channel ECoG signals. **a**. Neuronal raster plot reconstructed directly from SVCA, demonstrating effective preservation of information. **b**. Evaluation of information retained during reconstruction of neuronal calcium signals from SVCs (mean ± SEM). **c**. Neuronal raster plots predicted from multi-channel ECoG signals across multiple mice. Top: original raster plot, bottom: predicted raster plot.

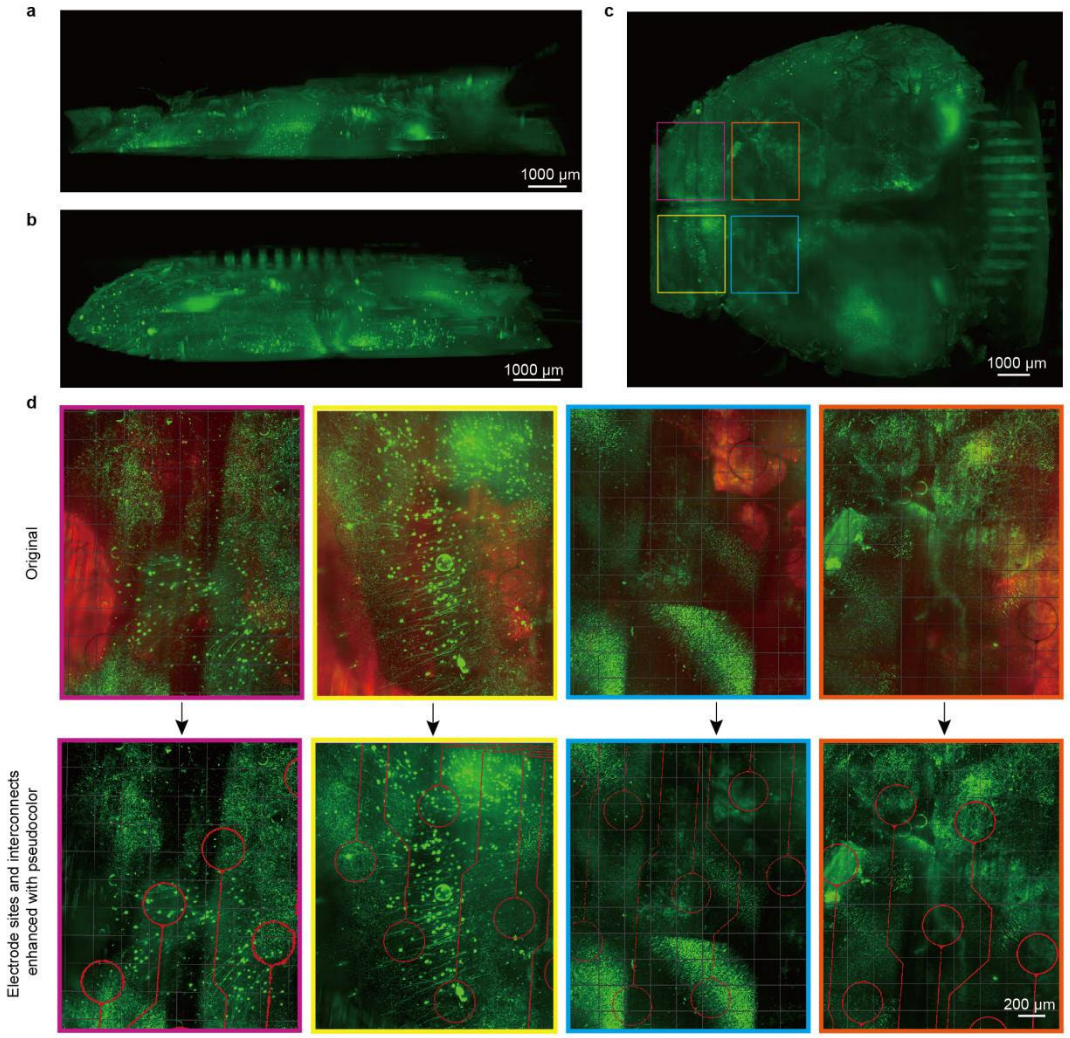

Supplementary Fig.15 | ECoG-guided localization of labeled neurons in Thy1-YFP mouse brain sections. a–c, Three orthogonal views of a mouse brain slice are shown from the frontal (**a**), left lateral (**b**), and dorsal (**c**) perspectives. **d**, An enlarged view of the brain regions marked in (c) with four colored boxes (purple, yellow, blue, and orange). For each region, both the original signal (top) and pseudocolor-enhanced images highlighting electrode sites and wires (bottom) are presented to illustrate the spatial relationship between the electrodes and the labeled neurons. The electrodes were instrumental in mapping signals both in vivo and in vitro.
