# Supplementary material for: Dynamic neuronal ensembles encode burst-suppression revealed by cortex-wide optical-electrical interfaces
Source: Nat Commun. 2026 Apr 29;17:5872. doi: 10.1038/s41467-026-72454-0 (PMC13333928; doi:10.1038/s41467-026-72454-0)
Supplement: Supplementary file 8 — Transparent Peer Review file [file 41467_2026_72454_MOESM8_ESM.pdf]

nature portfolio

Peer Review File

Dynamic neuronal ensembles encode burst-suppression revealed by cortex-wide optical-electrical interfaces

Corresponding Author: Professor Xiaochuan Dai

This file contains all reviewer reports in order by version, followed by all author rebuttals in order by version.

Version 0:

Reviewer comments:

Reviewer #1

(Remarks to the Author)
The manuscript integrates high-resolution optical imaging with microelectrode arrays to monitor activity across the dorsal cortex during anesthesia and awake states. The complementary high spatiotemporal resolution provided by this integration allows the authors to study the dynamics of burst-suppression events under isoflurane. The broad coverage of the microelectrode array, together with calcium signals from latge populations of neurons, makes this approach compelling. The authors have used their rich dataset to show that, contrary to the common assumption that burst-suppression events are global, these events exhibit spatiotemporal dynamics, initiating in lateral areas and propagating medially, with stronger synchronization between LFP and calcium signals around the midline. The manuscript is well-structured in presenting the methods and results; however, there are several major points that need attention before the work can be considered publication. My comments and questions are as follows:

1) Overall, the paper needs to do a better job in citing prior research. All the ideas discussed in the introduction (transparent electrodes, multimodal imaging recording, inferring calcium from ECoG) have been previously demonstrated by others. Authors need to include proper citations. Including citations will not make their paper weaker as novelty is in the application of multimodal approach into a specific neuroscience question related to burst-supression.
2) The last part (line 193) of “CODE system for simultaneous cortex-wide ECoG and calcium imaging” is misleading. For example, with what exactly are the superior chronic stability, optical transparency, and compatibility with calcium imaging being compared? Several recent studies have demonstrated transparent microelectrode arrays capable of stable chronic recordings from the cortical surface and compatibility with two-photon imaging. In addition, impedance alone is not an adequate performance metric. The electrodes used here are quite large, and low impedances in such cases are not unusual. A normalized impedance comparison would be more appropriate, and under such comparison, it is unlikely that the authors could claim superior impedance characteristics compared to similar works.
3) The comparison in Fig. 1i is, in my opinion, not fair. The listed works are mostly single-modality studies, while the multimodal ones have limited FoV due to their specific focus of their studies. This means that the plot effectively compares volumetric imaging with microelectrode arrays, which are fundamentally different modalities and therefore not directly comparable. I think the authors should remove this plot.
4) The number of neurons recorded for mouse 1 (the subject used for most analyses in the main figures) is notably higher than for mice 2 and 3. The authors should clearly state this, instead of emphasizing only the total number (17,000 neurons), which is somewhat misleading.
5) The manuscript does not discuss light-induced artifacts. Did the authors remove these artifacts, or were they not present in the electrophysiology recordings?
6) It is not clear how the authors calculated the SNR for their ECoG signals. A detailed explanation in the methods would be helpful.
7) Many of the closing statements in the results subsections are not sufficiently supported. For example:
• Line 271: The suggestion that coupling between ECoG and calcium signals during bursts or suppressions could mediate transitions is not convincing. To make such claims, more systematic studies are required, including beyond cortical regions. This is particularly important because cortical regions are relatively inactive during anesthesia, while subcortical areas remain active, yet this work does not include subcortical recordings.
• Line 386: The claim that “burst-related synchronization reflects a cortical attempt to restore long-range sensory integration pathways under deep anesthesia” is untested and not supported by the presented data, especially without subcortical recordings.

• Line 461: The suggestion that neurons' selective activation plays a preparatory or gating role in transitions toward bursts is again not substantiated by the current results.
• Line 486: The claim that wave propagation "may represent an intrinsic cortical attempt to re-engage distributed networks, potentially reflecting a homeostatic response to prolonged suppression" also lacks sufficient evidence.

8) In line 431, the authors claim that their cross-modality inference could serve as a metric for tracking consciousness state transitions. I believe this requires much stronger characterization. The authors have overlooked the fact that under anesthesia, neural activity collapses into lower dimensions, which likely explains why prediction accuracy is higher than in the awake state. Using the predictability of neural dynamics from ECoG signals as an indirect metric is, in my view, not appropriate when more reliable approaches are available. This claim is repeated again in the discussion (line 473).
9) In line 471, the authors correctly note that during awake states, neural dynamics are less correlated and calcium signals lie in a higher-dimensional space, making prediction more challenging. However, a recent study in Nature Nanotechnology (https://doi.org/10.1038/s41565-023-01576-z) demonstrated that high-frequency bands contain significant information about neural dynamics in deep brain layers. The authors could mention that with higher-density microelectrode arrays and smaller electrode sites, these results may need to be revisited.

Minor comments:.
• Line 351: "supervised clustering electrophysiological activity" should be "supervised clustering of electrophysiological activity."
• Line 375: The cited figure is incorrect; it should be Fig. 9.
• Line 451: "enabled identification two functionally" should be "enabled identification of two functionally."
• In Fig. 1c, the recording area is too small for readers to adequately evaluate the transparency of the electrode array, which makes this panel redundant unless the authors replace it with a zoomed image.
• The authors should specify which channel is used for representative examples in the main and extended figures (e.g., Fig. 3b).
• The x-axis in Fig. 4m needs clarification.

Reviewer #2

(Remarks to the Author)
This manuscript by Xiao et al combines the recent real-time, ultra-large-scale, high-resolution (RUSH) system and transparent 32-channel ECoG array and studies burst suppression phenomena in mouse models. The authors dubbed this combination the Cortex-wide Optical-electrical Dual-modal Explorer (CODE). They first characterized the transparent ECoG array's recording performance and longevity over 4 weeks. Then they investigated the burst suppression under isoflurane and examined the detailed on-off pattern in ECoG and Calcium activities. They further classified neurons into burst or suppression-related ensembles and examined neuronal ensemble-specific ECoG signals. Another discovery is that ECoG bursts rapidly propagate from bilateral sensory cortices to central motor areas with increasing synchrony with calcium activity. Finally, the team established a predictive model that infers calcium signals from ECoG features.

There is no doubt that this is a comprehensive summary of a large amount of work and data with thoughtful presentations. The technologies and methodologies in use are also cutting-edge. However, this reviewer cannot support the publication of this manuscript in Nature Communications due to its two major weaknesses.

1. Lack of fundamental innovation. While touted as a Cortex-wide Optical-electrical Dual-modal Explorer, the approach itself is really a combination of two published technologies, namely the RUSH wide-field Ca imaging system and ITO/PEDOT:PSS transparent ECoG arrays. The former was published in Nature Photonics 13, 809–816 (2019), and the latter was established by other research teams for quite a while now (see e.g., IEEE Trans Nanotechnol. 2017 Dec 20;17(4):701–704). Thus, while the work involves substantial systems neuroscience efforts, it lacks the kind of substantial innovation and quantum leap that are typically read in Nature Communications.

2. Lack of substantially novel findings. Many of the findings in neuroscience presented in this manuscript are not new. For example, first, Br J Anaesth 2025 Apr;134(4):1122-1133 has reported a similar on-off pattern in neural activities under isofluorane and performed a similar classification of neurons into burst-related and suppression-related ensembles.

Second, Front Syst Neurosci. 2021 Jan 12;14:599781 has reported the spatial structure and propagation of isoflurane-induced burst suppression, with much finer spatial resolution compared to ECoG-based clustering in this manuscript.

Third, Nature Nanotechnology 19, 504–513 (2024) has reported the prediction of cellular calcium activity at depth from surface potential recordings.

As a result, while this reviewer commends the grand effort and high-quality presentation, this manuscript appears to be more suitable for a more specialized journal in systems neuroscience, where the details of the findings can be fully debated and disseminated.

A minor issue is that none of the statistical results mentions the specific test used.

Reviewer #3

(Remarks to the Author)
I co-reviewed this manuscript with one of the reviewers who provided the listed reports. This is part of the Nature Communications initiative to facilitate training in peer review and to provide appropriate recognition for Early Career Researchers who co-review manuscripts.

Version 1:

Reviewer comments:

Reviewer #1

(Remarks to the Author)
The authors have addressed all my comments and revised unnecessarily strong claims that would need more experimental support. I do not have further comments. The manuscript can be accepted.

Reviewer #2

(Remarks to the Author)
This reviewer appreciates the extensive effort that the authors have taken to address the previous comments. In the spirit that science thrives on criticism, this reviewer has the following re-rebuttal:

1. “prior transparent electrode demonstrations have been limited by interfacial instability, optical degradation, or restricted coverage and longevity.”

This reviewer disagrees. Interfacial instability or optical degradation are not major issues from previous transparent electrodes. Previous demonstrations may have specific designs for their corresponding studies, but are not fundamentally restricted in coverage. Lastly, longevity is rarely an issue for animal ECoG.

2. “Conventional soft ECoG electrodes often fracture or lose functionality when compressed under a coverslip,”

This reviewer disagrees. How the brain is closed matters indeed, but flexible electrodes should not easily fracture under a coverslip that is flat.

3. This manuscript indeed conducted much wider field of view imaging than Br J Anaesth 2025 Apr;134(4):1122-1133, and achieved faster imaging than Front Syst Neurosci. 2021 Jan 12;14:599781. However, these are all enabled by the RUSH system, which has been published and has little to do with multimodality. At a minimum, the results and figures should focus on the cortex-wide maps and ms-scale dynamics.

That being said, this reviewer can appreciate the strong utility demonstrations from the integrated system, multimodal analytics and their potential broad appeal to the systems neuroscience research community.

In summary, this reviewer agrees with reviewer 1 that for this manuscript, the “novelty is in the application of multimodal approach into a specific neuroscience question related to burst-suppression.” Therefore, the authors should at least downplay many big innovation claims on the technology side, revise many seemingly inaccurate statements, and better report their biological data/findings.

Reviewer #3

(Remarks to the Author)
I co-reviewed this manuscript with one of the reviewers who provided the listed reports. This is part of the Nature Communications initiative to facilitate training in peer review and to provide appropriate recognition for Early Career Researchers who co-review manuscripts.

Open Access This Peer Review File is licensed under a Creative Commons Attribution 4.0 International License, which permits use, sharing, adaptation, distribution and reproduction in any medium or format, as long as you give appropriate credit to the original author(s) and the source, provide a link to the Creative Commons license, and indicate if changes were made.
In cases where reviewers are anonymous, credit should be given to 'Anonymous Referee' and the source.
The images or other third party material in this Peer Review File are included in the article's Creative Commons license, unless indicated otherwise in a credit line to the material. If material is not included in the article's Creative Commons license and your intended use is not permitted by statutory regulation or exceeds the permitted use, you will need to obtain permission directly from the copyright holder.
To view a copy of this license, visit https://creativecommons.org/licenses/by/4.0/

Manuscript: NCOMMS-25-58362-T

Title: Dynamic neuronal ensembles encode burst-suppression revealed by cortex-wide optical-electrical interfaces

Dec 8, 2025

Point by point responses to reviewer comments:

Reviewer #1:

Comments: The manuscript integrates high-resolution optical imaging with microelectrode arrays to monitor activity across the dorsal cortex during anesthesia and awake states. The complementary high spatiotemporal resolution provided by this integration allows the authors to study the dynamics of burst-suppression events under isoflurane. The broad coverage of the microelectrode array, together with calcium signals from latge populations of neurons, makes this approach compelling. The authors have used their rich dataset to show that, contrary to the common assumption that burst-suppression events are global, these events exhibit spatiotemporal dynamics, initiating in lateral areas and propagating medially, with stronger synchronization between LFP and calcium signals around the midline. The manuscript is well-structured in presenting the methods and results; however, there are several major points that need attention before the work can be considered publication. My comments and questions are as follows:

Response: We sincerely thank the reviewer for the thorough and constructive evaluation of our manuscript. We greatly appreciate the positive assessment of our integrated high-resolution optical imaging and microelectrode array platform, as well as the recognition of the significance of our findings on the spatiotemporal dynamics of burst-suppression under isoflurane anesthesia. We are grateful for the reviewer's insightful comments and questions, which have substantially helped us improve the clarity, rigor, and interpretation of the study.

Below, we provide detailed point-by-point responses. Reviewer comments are reproduced in black, followed by our responses.

Q1: Overall, the paper needs to do a better job in citing prior research. All the ideas discussed in the introduction (transparent electrodes, multimodal imaging recording, inferring calcium from ECoG) have been previously demonstrated by others. Authors need to include proper citations. Including citations will not make their paper weaker as novelty is in the application of multimodal approach into a specific neuroscience question related to burst-suppression.

Response: We thank the reviewer for this important comment. We fully agree that the Introduction should more

thoroughly acknowledge prior advances in transparent electrodes, multimodal electrophysiology–imaging approaches, and efforts to infer neuronal activity from ECoG signals. We also appreciate the reviewer's perspective that proper citation does not detract from the novelty of our study, which lies in the unified cortex-wide platform and its application to uncover previously unrecognized spatiotemporal structure in burst-suppression.

In response, we have substantially revised and expanded the Introduction to better situate our work within the existing literature. The updated text now includes comprehensive citations covering key developments in (i) transparent electrode technologies, (ii) multimodal optical–electrical recordings, and (iii) cross-modal inference of calcium or spiking activity from surface electrophysiology. These additions clarify how our approach builds upon and differs from prior studies.

The revised Introduction appears in the updated manuscript (Lines 69–90). The relevant section is reproduced below for convenience:

Revised Introduction excerpt (Lines 69–90):

Electroencephalogram (EEG) and ECoG recordings have traditionally portrayed burst-suppression as a globally synchronized process[1-4]. However, recent discoveries—such as traveling waves across cortical areas[5]—challenge this view and suggest the presence of spatiotemporally structured dynamics. While EEG or ECoG recordings offer millisecond-level temporal precision, their poor spatial resolution precludes insights into single-cell behaviors. On the other hand, two-photon microscopic calcium imaging has provided single-neuron resolution within sub-millimeter field-of-view (FOV), revealing synchronized pyramidal neuron activity during anesthesia. [6] Yet, these optical approaches suffer from limited spatial coverage and frame rates insufficient to resolve rapid brain-state transitions across extensive cortical regions. ECoG fabrication increasingly relies on transparent electrode technologies—including graphene[7-10], metal nanoparticles[11, 12], carbon nanotubes[13], hydrogel[14, 15], and PEDOT-only systems[16-18], which make it possible to combine calcium imaging with electrophysiology. Attempts to integrate these modalities, such as pairing two-photon calcium imaging with transparent ECoG arrays[7, 13, 19] or with whole-cell patch-clamp recordings[20] have offered improved spatial resolution but remain constrained to small field of view and relatively sparse neuronal populations, or employing methods such as wide-field imaging combined with ECoG to monitor large cortical areas[21], low spatial resolutions, limited the identification of specific functional neuron types. Additionally, several studies have explored the inference of calcium or spiking activity directly from surface electrophysiology, revealing both the potential and limitations of such cross-modal prediction approaches[22-24].

Q2: The last part (line 193) of "CODE system for simultaneous cortex-wide ECoG and calcium imaging" is misleading. For example, with what exactly are the superior chronic stability, optical transparency, and compatibility with calcium imaging being compared? Several recent studies have demonstrated transparent

microelectrode arrays capable of stable chronic recordings from the cortical surface and compatibility with two-photon imaging.

In addition, impedance alone is not an adequate performance metric. The electrodes used here are quite large, and low impedances in such cases are not unusual. A normalized impedance comparison would be more appropriate, and under such comparison, it is unlikely that the authors could claim superior impedance characteristics compared to similar works.

Response: We thank the reviewer for these critical and constructive comments. We apologize for the unintended implication in the previous text. Our intention was not to claim that the CODE system offers superior chronic stability, optical transparency, or imaging compatibility relative to all previously reported transparent microelectrode arrays. Rather, the contribution of CODE lies in integrating cortex-wide optical access, single-cell–resolution calcium imaging, and millisecond-scale electrophysiology within a unified and chronically stable platform. To avoid any misleading interpretation, we have revised the corresponding statements in the manuscript to clarify that our focus is on the system-level multimodal integration rather than on outperforming prior technologies on individual metrics. We have also removed the previous Figure 1i to prevent overemphasis on this point.

In the revised manuscript, we now explicitly state that the chronic stability, optical transparency, and imaging compatibility are demonstrated as attributes of the integrated system, without implying superiority over existing transparent MEA designs.

Regarding the impedance concern, we appreciate the reviewer's guidance. Our original intention was to report impedance simply as a basic specification confirming that the electrodes are well suited for reliable ECoG recordings, as impedance values below approximately 100 kΩ at 1 kHz are widely considered suitable for cortical surface neural signal acquisition [25]. We agree that impedance alone is not a sufficient performance metric and that comparisons based on absolute impedance would not be appropriate given the relatively large electrode size used here. In the revised text, we therefore present impedance measurements solely as validation of recording capability, without any comparative claims. The relevant section in the main text has been rewritten accordingly.

Revised manuscript excerpt (Lines 171–180):

We characterized electrode performance via electrochemical impedance spectroscopy (EIS). Bare ITO electrodes exhibit an average impedance of 67.7 ± 16.7 kΩ at 1 kHz, which is significantly reduced to 26.8 ± 2.5 kΩ at 1 kHz following PEDOT:PSS coating (Fig.1g,h). Impedance values in this range are widely considered suitable for reliable cortical surface electrophysiological recording[25].Cyclic voltammetry (CV) revealed enhanced cathodic charge storage capacity [26], as indicated by increased shaded area under the CV curve (Extended Data Fig.1e), supporting the potential for future stimulation applications. Together, the CODE system integrates high spatiotemporal resolution electrophysiology with large-scale optical access, enabling cortex-wide, high-throughput, dual-modality neural recording.

Q3. The comparison in Fig. 1i is, in my opinion, not fair. The listed works are mostly single-modality studies, while the multimodal ones have limited FoV due to their specific focus of their studies. This means that the plot effectively compares volumetric imaging with microelectrode arrays, which are fundamentally different modalities and therefore not directly comparable. I think the authors should remove this plot.

Response: We thank the reviewer for this insightful and well-taken comment. We agree that directly comparing spatial coverage across fundamentally different recording modalities within a single quantitative plot is not appropriate and may lead to misleading interpretations. In particular, volumetric optical imaging and surface microelectrode array recordings differ substantially in their measurement principles, dimensionality, and intended experimental scope, making direct comparison problematic.

While our original intention was to provide contextual reference for the capabilities of the CODE system, we acknowledge that the visualization in Fig. 1i did not constitute a fair or informative comparison.

In accordance with the reviewer's suggestion, we have removed Fig. 1i from the revised manuscript. The associated text has been rewritten to focus instead on a qualitative description of the system-level integrative capabilities of CODE—namely, its ability to combine cortex-wide coverage with single-neuron–resolution optical access and millisecond-scale electrophysiology—without attempting direct quantitative comparisons across disparate modalities.

We believe that this revision improves the clarity and accuracy of the manuscript and more appropriately conveys the contribution of our integrated recording platform.

Q4. The number of neurons recorded for mouse 1 (the subject used for most analyses in the main figures) is notably higher than for mice 2 and 3. The authors should clearly state this, instead of emphasizing only the total number (17,000 neurons), which is somewhat misleading.

Response: We thank the reviewer for highlighting this important point, which helps improve the transparency and clarity of our data reporting. We agree that the number of neurons extracted varied across individual mice. This variability primarily reflects differences in GCaMP expression levels and imaging quality between animals, both of which directly influence the number of neurons detected by the analysis pipeline.

Importantly, despite these differences in neuron yield, all mice exhibited consistent trends in the burst-suppression analyses, supporting the robustness of the observed phenomena.

To further strengthen the reliability of our conclusions, we have repeated the experiments and incorporated additional datasets into the revised manuscript and figures. The newly added animals also show high numbers of detected layer 2/3 neurons (Mouse 53: 19,864; Mouse 62: 18,033; Mouse 600: 13,636; Mouse 456: 17,781), further confirming the reproducibility of our results. We now explicitly report per-mouse neuron counts in both

the Methods and Results sections for clarity. The additional datasets have also been included in Extended Data Fig. 4.

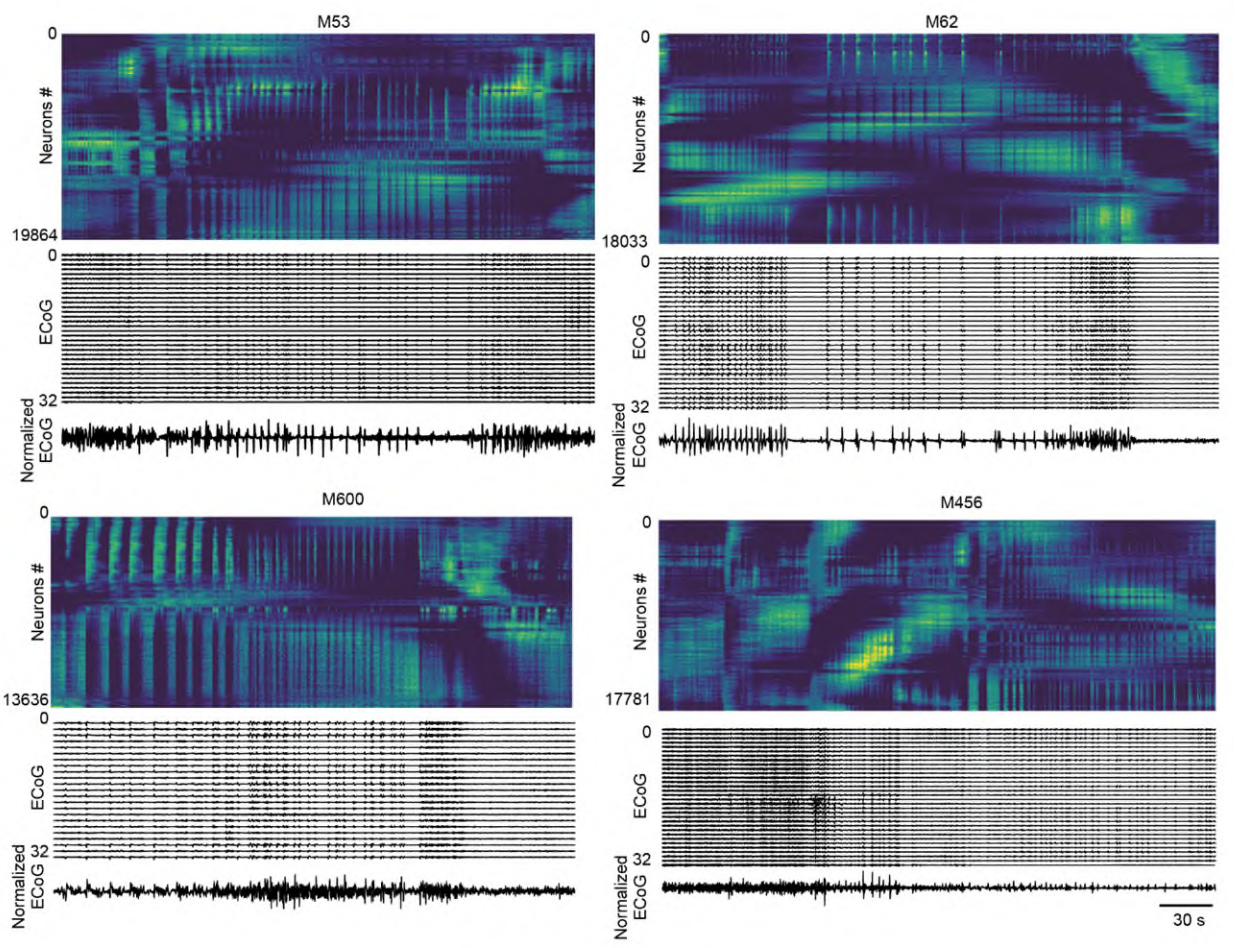

Figure R1. The neuron number for each mouse. Neuronal raster plots (top), and the corresponding raw ECoG signals and a typical normalized ECoG signal (bottom) during burst and suppression periods across multiple mice.

Q5. The manuscript does not discuss light-induced artifacts. Did the authors remove these artifacts, or were they not present in the electrophysiology recordings?

Response: We thank the reviewer for raising this important question regarding potential light-induced artifacts in the electrophysiological recordings. In our experiments, we did not observe detectable light-induced artifacts in the ECoG signals during simultaneous calcium imaging.

To further and directly address this concern, we performed additional control experiments specifically designed to test for illumination-related artifacts. In these experiments, ECoG recordings were acquired while the

excitation light was periodically switched on and off every 10 seconds under pulsed illumination conditions. Representative electrophysiological traces aligned to the onset and offset of illumination are shown in Fig. R2.

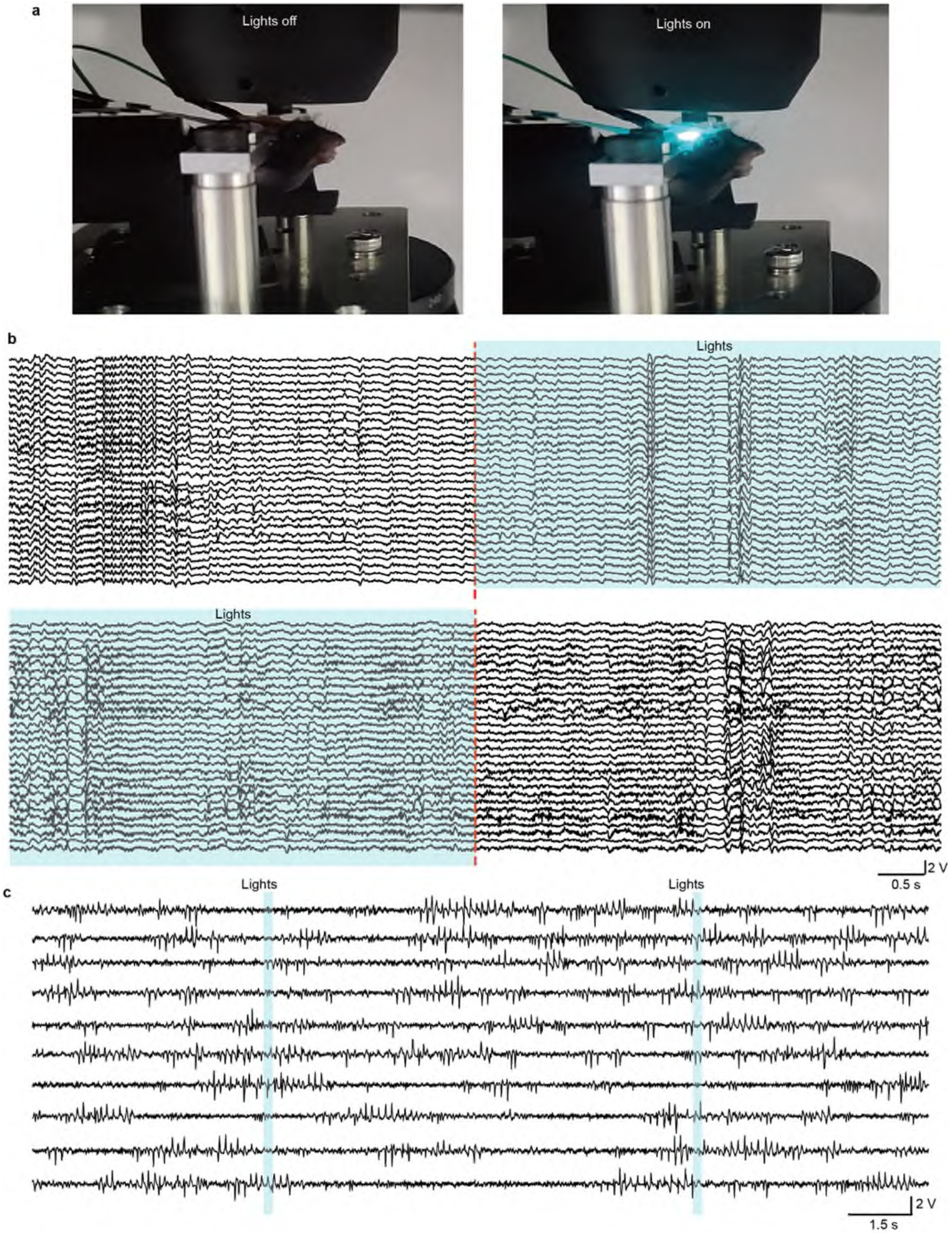

Figure R2. Light-induced artifacts on electrophysiology recordings. Light-induced artifacts in electrophysiological recordings. **a.** Images of the mouse brain under the microscope with the illumination off (left) and on (right). **b**. Electrophysiological recordings acquired during continuous illumination used for calcium imaging. **c**. Electrophysiological recordings acquired during pulsed illumination used for calcium imaging.

Notably, no transient disturbances, synchronous deflections, or systematic changes in the ECoG signals were observed at either light onset or offset. The electrical recordings remained stable throughout the imaging sessions, indicating that the optical excitation did not introduce measurable artifacts into the electrophysiological data.

These control results have now been incorporated into the revised manuscript (Lines 220–223) and are presented in Extended Data Fig. 2.

We believe that the observed robustness against light-induced artifacts is attributable to the optical and materials design of the electrode array. First, the gold interconnects are fabricated with minimal thickness, substantially reducing their interaction with incident light. Second, and more importantly, the electrode sites themselves are composed of materials with high optical transmittance (ITO/PEDOT:PSS), which markedly suppress the generation of photoelectric currents that could otherwise introduce artifacts into electrophysiological recordings.

As a result, even under pulsed illumination, the electrode design exhibits minimal susceptibility to light-induced interference. We note that at substantially higher illumination intensities, photoelectric artifacts may arise, particularly in electrodes fabricated from conventional opaque metals such as gold. However, for all experiments reported in this study, the excitation power was maintained within a range that the electrode design robustly tolerates. Artifact-free signal quality was verified both by visual inspection and by quantitative analysis.

These additional control results, together with representative raw electrophysiological traces, have been included in the revised manuscript as a Supplementary Figure to further document the absence of light-induced artifacts under all experimental conditions relevant to this study.

Q6. It is not clear how the authors calculated the SNR for their ECoG signals. A detailed explanation in the methods would be helpful.

Response: We thank the reviewer for this helpful suggestion and agree that the original manuscript did not provide sufficient detail regarding the SNR calculation. We have now added a comprehensive description of the Signal-to-Noise Ratio (SNR) computation to the revised Methods section (Lines 678–691).

Briefly, SNR for the ECoG recordings was quantified in the frequency domain using the power spectral density (PSD), a commonly used and robust approach for assessing electrophysiological signal quality. The SNR was calculated as:

$$SNR = 10 \times log_{10}(\frac{P_s}{P_n})$$

where:

- P_s is the average signal power within the frequency band of interest (1–100 Hz for our ECoG analysis);

- P_n is the average noise power. The noise floor was estimated from the same recording by averaging the PSD within a high-frequency band (500–1000 Hz), where true neural activity is negligible, providing a stable estimate of baseline noise.

Estimating the noise floor from this high-frequency range is a standard practice in electrophysiological analyses [27]. The revised Methods section now explicitly states:

Revised Methods excerpt (Lines 678–691):

Signal-to-Noise Ratio (SNR) calculation for ECoG signals. *The Signal-to-Noise Ratio (SNR) of the ECoG recordings was quantified in the frequency domain using the power spectral density (PSD). For each channel, PSD estimates were obtained using Welch's method with 1-s Hamming windows and 50% overlap. The SNR was computed as:*

$$SNR = 10 \times log_{10}(\frac{P_s}{P_n}) \qquad \text{Equation 1}$$

where P_s represents the average signal power within the frequency band of interest (1–100 Hz), corresponding to the range containing physiologically relevant ECoG activity. Noise power P_n was estimated from the same recording by calculating the mean PSD within a high-frequency band (500–1000 Hz), where neural activity is negligible and the spectrum reflects baseline noise. This high-frequency band is commonly used to approximate the noise floor in electrophysiological recordings [27]. The resulting SNR provides a channel-specific measure of recording quality and was used for data inclusion and quality assessment.

Q7. Many of the closing statements in the results subsections are not sufficiently supported.

Response: We thank the reviewer for these critical observations regarding the interpretation of our results. We agree that several closing statements in the Results section could be interpreted as extending beyond what is directly supported by the current dataset, particularly given the absence of subcortical recordings. In response, we have thoroughly revised the manuscript to ensure that all conclusions are more carefully aligned with the presented evidence. Specifically, we have removed or softened mechanistic or goal-directed interpretations, reframing them as descriptive observations or correlative associations where appropriate.

Below, we address each point in detail.

- a. Line 271: The suggestion that coupling between ECoG and calcium signals during bursts or suppressions could mediate transitions is not convincing. To make such claims, more systematic studies are required, including beyond cortical regions. This is particularly important because cortical regions are relatively inactive during anesthesia, while subcortical areas remain active, yet this work does not include subcortical recordings.

Response: We agree that the original wording could be interpreted as implying a causal role in mediating state transitions, which is not supported by our cortical-only recordings. Although the original sentence used

speculative language (e.g., “may”), we recognize that it could still be misread as a mechanistic claim. To avoid this ambiguity, we have revised the text to describe only the observed association, without implying causality.

The original sentence:

These two neuronal ensembles appear to be closely coupled to the electrical rhythms of burst-suppression and may play a key role in mediating the transitions between these alternating states.

has been changed to:

These two neuronal ensembles appear to be closely coupled to the electrical rhythms of burst-suppression, and display dynamically alternating activation patterns that accompany transitions between burst and suppression states.

This revision appears in the updated manuscript (Lines 257–263).

- b. Line 386: The claim that “burst-related synchronization reflects a cortical attempt to restore long-range sensory integration pathways under deep anesthesia” is untested and not supported by the presented data, especially without subcortical recordings.

Response: We appreciate this insightful comment and agree that the original phrasing implied a goal-directed or functional interpretation (“attempt to restore”), which is not warranted by the data. To avoid overinterpretation, we have revised the statement to focus solely on the observed cortical dynamics during bursts, without invoking functional intent.

The original sentence:

We hypothesize that burst-related synchronization reflects a cortical attempt to restore these long-range sensory integration pathways under deep anesthesia.

has been changed to:

Burst-related synchronization reflects a globally coordinated spiking pattern across the cortex, in which cortical activity shifts from a relatively suppressed state to a more active state during bursts.

This change can be found in the revised manuscript (Lines 382–384).

- c. Line 461: The suggestion that neurons’ selective activation plays a preparatory or gating role in transitions toward bursts is again not substantiated by the current results.

Response: We agree that the original phrasing implied an untested functional role (e.g., “preparatory” or “gating”) in driving state transitions. Our observations are limited to correlative cortical activity patterns and do not support causal inference. To avoid overstatement, we have revised the sentence to describe only the observed neuronal activity during suppression.

The original sentence:

By capturing cortex-wide single-neuron activity, CODE uncovered these neurons' selective activation during suppression events—suggesting they may play a preparatory or gating role in transitioning toward bursts.

has been changed to:

By capturing cortex-wide single-neuron activity, CODE uncovered these neurons' selective activation during suppression events, indicating that suppression is not a state of complete silence as implied by the macroscopic electrical signal.

This revision appears in the updated manuscript (Lines 459–462).

• d. Line 486: The claim that wave propagation "may represent an intrinsic cortical attempt to re-engage distributed networks, potentially reflecting a homeostatic response to prolonged suppression" also lacks sufficient evidence.

Response: We thank the reviewer for noting that the original phrasing implied a goal-directed or homeostatic mechanism, which is beyond what our data support. To avoid such interpretation, we revised the statement to focus exclusively on the observed propagation dynamics accompanying state transitions.

The original sentence:

Such propagation may represent an intrinsic cortical attempt to re-engage distributed networks, potentially reflecting a homeostatic response to prolonged suppression.

has been changed to:

Such propagation reliably accompanies transitions from suppression to bursts, suggesting that cortical activity is not random but instead follows structured dynamics during anesthetized unconsciousness.

This change can be found in the revised manuscript (Lines 497–500).

By implementing these revisions, we believe the manuscript now more accurately reflects the correlative nature of our findings while maintaining their significance. The revised text avoids mechanistic overinterpretation and instead emphasizes empirically supported observations that motivate future investigation.

Q8. In line 431, the authors claim that their cross-modality inference could serve as a metric for tracking consciousness state transitions. I believe this requires much stronger characterization. The authors have overlooked the fact that under anesthesia, neural activity collapses into lower dimensions, which likely explains why prediction accuracy is higher than in the awake state. Using the predictability of neural dynamics from ECoG signals as an indirect metric is, in my view, not appropriate when more reliable approaches are available. This claim is repeated again in the discussion (line 473).

Response: We thank the reviewer for this insightful and important comment, which raises a fundamental issue regarding the interpretation of our cross-modal inference results. We fully agree that the collapse of neural activity into a lower-dimensional dynamical regime under anesthesia is a defining characteristic of unconscious brain states and is a key factor underlying the higher prediction accuracy observed during burst-suppression, as also noted in our original manuscript.

In response to this comment, we have substantially refined our interpretation and toned down the associated claims. Specifically, we no longer present cross-modal prediction accuracy as a direct or standalone metric for tracking consciousness state transitions. We have removed statements implying that prediction accuracy itself could serve as a quantitative measure of consciousness, including the sentence:

Beyond demonstrating modality compatibility, this approach may offer a powerful metric for tracking consciousness state transitions or diagnosing cortical disorganization in pathological conditions.

Instead, we now emphasize that state-dependent changes in predictability reflect underlying shifts in the complexity of cortical dynamics, rather than consciousness per se. The revised text in the Results section (Lines 428–433) now reads:

The state-dependent variation in cross-modal prediction accuracy likely reflects the fundamental shift in the complexity of cortical dynamics between unconscious (burst-suppression) and awake states. The higher predictability during anesthesia is consistent with a collapse into lower-dimensional, more stereotyped dynamics, while the lower predictability in the awake state mirrors the high-dimensional, complex neural activity associated with consciousness.

Consistent revisions were also made in the Discussion section. The original statement:

These results suggest that cross-modal predictability may serve as a proxy for the degree of coherence in cortical activity under different brain states, and potentially offer new metrics for assessing consciousness or network integrity.

has been revised to (Lines 473–475):

Therefore, the degree to which large-scale population activity can be predicted from macroscopic ECoG signals may provide an indirect, yet informative, signature of the complexity of cortical dynamics, which is intrinsically linked to brain state.

We fully agree with the reviewer that established measures—such as spectral power, entropy, or complexity metrics—are more direct and widely accepted proxies for assessing consciousness and brain state. Our intention is not to propose cross-modal predictability as a replacement for these metrics, but rather to highlight that the CODE framework offers a complementary perspective by explicitly linking macroscopic electrical signals to microscopic population dynamics within a unified experimental paradigm.

We appreciate the reviewer's guidance, which has helped us clarify the conceptual framing and strengthen the rigor of this part of the manuscript.

Q9. In line 471, the authors correctly note that during awake states, neural dynamics are less correlated and calcium signals lie in a higher-dimensional space, making prediction more challenging. However, a recent study in Nature Nanotechnology (https://doi.org/10.1038/s41565-023-01576-z) demonstrated that high-frequency bands contain significant information about neural dynamics in deep brain layers. The authors could mention that with higher-density microelectrode arrays and smaller electrode sites, these results may need to be revisited.

Response: We sincerely thank the reviewer for this excellent and forward-looking suggestion. We fully agree with the reviewer's point and appreciate the reference to the recent Nature Nanotechnology study (https://doi.org/10.1038/s41565-023-01576-z), which compellingly demonstrates that high-frequency components of electrophysiological signals can encode rich information about neural dynamics in deeper cortical layers, which is not accessible through surface calcium imaging alone.

In response, we have incorporated this important perspective into the Discussion section of the revised manuscript. We now explicitly acknowledge that the current cross-modal prediction performance, particularly in the awake state where neural activity becomes less correlated and higher dimensional, is likely constrained by the spatial resolution and frequency bandwidth of the standard ECoG array used in this study. We further note that high-frequency signals, such as spiking-related activity and high-gamma oscillations, are only partially captured with the present electrode configuration.

We therefore propose that future studies employing next-generation, high-density microelectrode arrays with smaller electrode sites and broader recording bandwidths may enable the capture of these high-frequency components. Such advances could allow a more detailed electrophysiological representation of local neural computation and, in turn, improve cross-modal inference of population calcium dynamics—even in the high-dimensional regime of wakefulness.

This addition strengthens the Discussion by situating our findings within the context of emerging electrophysiological technologies and by outlining a clear path for revisiting and extending our results as recording capabilities continue to advance.

The revised text has been added to the Discussion section (Lines 475–486) and now reads:

Recent evidence suggests that high-frequency bands can encode detailed neural activity originating from deeper cortical layers—information that is inaccessible to surface calcium imaging[22]. Our current ECoG-based predictions, especially during awake states when neural activity becomes high dimensional and less correlated, may constrained by the spatial resolution and limited bandwidth of the standard ECoG array used here. Looking forward, the use of next-generation, high-density microelectrode arrays with smaller electrode

sites and broader recording bandwidths may allow future studies to capture these high-frequency signals, including spiking-related activity and high-gamma oscillations. Such advances could enable more accurate cross-modal inference of neural population dynamics, even during wakefulness, by providing a more complete electrophysiological representation of local neural computation.

Q10. Minor comments:

- a. Line 351: “supervised clustering electrophysiological activity” should be “supervised clustering of electrophysiological activity.”

Response: We thank the reviewer for noting this typographical error. The phrase has been corrected from “*supervised clustering electrophysiological activity*” to “*supervised clustering of electrophysiological activity*” in the revised manuscript (Line 345).

- b. Line 375: The cited figure is incorrect; it should be Fig. 9.

Response: We thank the reviewer for pointing out this citation error. The figure reference has been corrected in the revised manuscript and now correctly refers to the appropriate Extended Data figure (Line 360).

- c. Line 451: “enabled identification two functionally” should be “enabled identification of two functionally.”

Response: We thank the reviewer for identifying this grammatical error. The phrase has been corrected accordingly in the revised manuscript (Line 451).

- d. In Fig. 1c, the recording area is too small for readers to adequately evaluate the transparency of the electrode array, which makes this panel redundant unless the authors replace it with a zoomed image.

Response: We thank the reviewer for this helpful suggestion and agree that the original view in Fig. 1c did not sufficiently convey the transparency of the electrode array. We have therefore replaced the panel with a higher-magnification image that more clearly illustrates the recording area and electrode transparency. The updated visualization is shown in Fig. 1c–d and is further supported by a zoomed view in Supplementary Fig. S1d (see also Fig. R3).

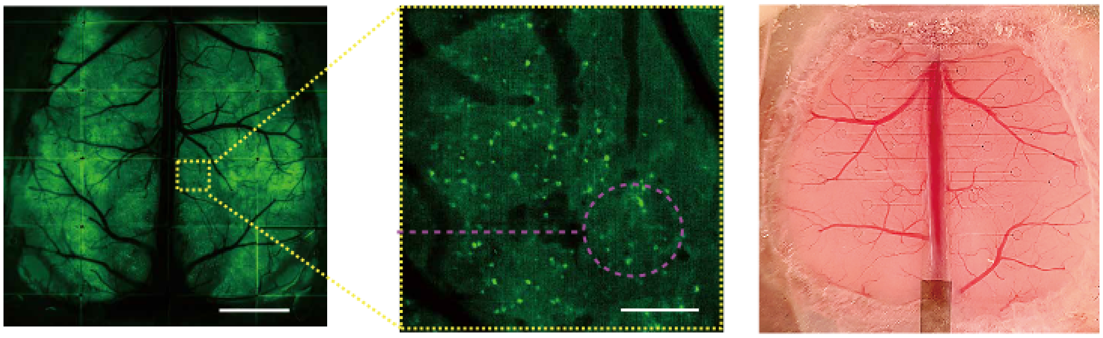

Figure R3. The transparent electrode array image. Wide-field calcium imaging of the mouse cortex via CODE, showing clear visualization of individual neurons, Scale bar, 2 mm, (Left), magnified view of the region, with the purple dashed circle indicating an electrode site on the transparent ECoG array, Scale bar, 200 μm, (middle), and the exposed cortex with clear blood vessels beneath the transparent neural interface glass (right).

- e. The authors should specify which channel is used for representative examples in the main and extended figures (e.g., Fig. 3b).

Response: We thank the reviewer for this valuable suggestion. In the revised manuscript, we now explicitly specify the channel used for all representative electrophysiological examples shown in both the main and extended figures. Specifically, the ECoG traces and corresponding spectrograms are taken from a representative electrode (channel 25), as now indicated in the relevant figure captions (including Fig. 3b) and detailed in the Methods section (Line 1260). The revised text reads:

…the ECoG waveform and its corresponding spectrogram from a representative electrode (channel 25)…

- f. The x-axis in Fig. 4m needs clarification.

Response: We thank the reviewer for pointing out the need for clarification. In the revised manuscript, we have updated the x-axis label in Fig. 4m to explicitly indicate the plotted variable and its units. In addition, the figure caption has been revised to clearly describe the meaning of the x-axis. These changes ensure that the figure can be interpreted unambiguously. The updated figure and corresponding text have been included in the revised manuscript.

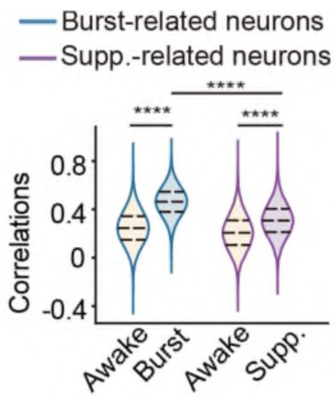

Figure R4. The violin plot compares the average correlations within burst-related neurons and suppression-related neurons, respectively, across awake stage, burst stage and suppression stages.

Reviewer #2

Comments: This manuscript by Xiao et al combines the recent real-time, ultra-large-scale, high-resolution (RUSH) system and transparent 32-channel ECoG array and studies burst suppression phenomena in mouse models. The authors dubbed this combination the Cortex-wide Optical-electrical Dual-modal Explorer (CODE). They first characterized the transparent ECoG array's recording performance and longevity over 4 weeks. Then they investigated the burst suppression under isoflurane and examined the detailed on-off pattern in ECoG and Calcium activities. They further classified neurons into burst or suppression-related ensembles and examined neuronal ensemble-specific ECoG signals. Another discovery is that ECoG bursts rapidly propagate from bilateral sensory cortices to central motor areas with increasing synchrony with calcium activity. Finally, the team established a predictive model that infers calcium signals from ECoG features.

There is no doubt that this is a comprehensive summary of a large amount of work and data with thoughtful presentations. The technologies and methodologies in use are also cutting-edge. However, this reviewer cannot support the publication of this manuscript in Nature Communications due to its two major weaknesses.

Response: We thank the reviewer for the careful and detailed evaluation of our manuscript and for acknowledging the technical sophistication, scale of the dataset, and the breadth of analyses presented. We appreciate the reviewer's recognition that this study represents a comprehensive effort combining advanced technologies with extensive experimental data.

At the same time, we believe that some aspects of the novelty and significance of this study may not have been fully appreciated, and we therefore clarify below the basis of our contribution.

1. CODE represents a qualitative technological innovation, not an incremental combination

We fully agree with the reviewer that wide-field calcium imaging and transparent ECoG arrays have each been reported previously. However, the primary innovation of CODE does not lie in the existence of these individual components, but in the creation of a **single and integrated platform** that achieves a combination of capabilities that were previously incompatible in practice.

Specifically, CODE simultaneously provides:

- **cortex-wide spatial coverage**,
- **single-neuron–resolution optical access**
- **millisecond-scale temporal precision**,
- **and stable chronic operation (>7 weeks),**

all within a single implantable cranial window.

This combination cannot be achieved through a straightforward "A + B" assembly of existing tools. In practice, conventional flexible ECoG arrays are mechanically incompatible with rigid cranial windows, while prior

transparent electrode demonstrations have been limited by interfacial instability, optical degradation, or restricted coverage and longevity.

By monolithically patterning ITO/PEDOT:PSS electrodes directly onto a glass cranial window through MEMS fabrication and interface engineering, CODE establishes a plug-and-play, electrode-integrated cranial window that maintains optical transparency and stable electrophysiological performance over weeks. We therefore view CODE as a **new class of multimodal neural interface**, rather than an incremental combination of previously reported technologies.

2. The integrated capability of CODE enabled fundamentally new biological insights

Beyond technical innovation, the key scientific contribution of this work lies in the biological discoveries that were **only made possible** by this integrated platform. While prior studies have examined isolated aspects of burst–suppression using either local calcium imaging or surface electrophysiology, none were able to simultaneously observe cortex-wide electrical dynamics and single-cell–resolved population activity across nearly the entire dorsal cortex.

This unique capability enabled several findings that go beyond the existing literature:

a. Cortex-wide identification and mapping of functional neuronal ensembles

Previous studies typically sampled tens to hundreds of neurons within a limited cortical region. By tracking >17,000 neurons across the cortex, we identified burst-related and suppression-related neuronal ensembles distributed across multiple cortical areas, revealing a large-scale organizational principle that could not be inferred from local recordings.

b. Discovery of dynamic ensemble switching across burst–suppression cycles

Contrary to the assumption that neurons have fixed roles during burst or suppression, we found that ensemble membership is highly dynamic and systematically reorganizes with anesthetic depth and suppression duration. This phenomenon was previously inaccessible due to limited neuronal sampling.

c. Population synchrony bifurcation at single-neuron resolution

We uncovered a cortex-wide bifurcation in population dynamics, in which burst-ensemble neurons exhibit strong, rapid synchrony while suppression-ensemble neurons remain asynchronous. This systems-level distinction requires simultaneous macro–micro resolution and has not been described previously.

d. Millisecond-scale mapping of burst onset propagation

CODE revealed a consistent, rapid propagation pathway from bilateral sensory cortices toward central motor areas during burst onset. Prior studies focused primarily on slower calcium waves during burst maintenance and lacked the temporal resolution to resolve this transition window.

e. Direct macro–micro linkage between ECoG waves and neuronal population dynamics

By recording ECoG and single-neuron activity simultaneously across the cortex, we demonstrated a progressive and spatially heterogenous alignment between ECoG propagation and underlying neuronal synchrony, providing a direct cross-scale linkage that was previously unavailable.

f. State-dependent predictability between electrophysiology and calcium activity

We showed that the ability to infer calcium activity from ECoG features is strongly brain-state dependent—maximal during burst-suppression and minimal during wakefulness—offering new insight into how global brain state shapes cross-modal coupling.

3. Contextualization with prior work

To clarify how these advances relate to existing literature, we compared with the related work mentioned in the major concerns through technology and also biological findings (Table R1 and Discussion Lines 501-522), contrasting prior multimodal studies with the present work. This comparison highlights that earlier studies were constrained by limited field of view, sparse neuronal sampling, or single-modality focus, whereas CODE uniquely enables cortex-wide, single-neuron–resolved, temporally precise investigations. Only when we can record the a large number of neurons accompany with corresponding ECoG signals across wide cortex, can we define two neuronal ensembles and also compare the distribution across wide cortex, different firing patterns and dynamic changes between them.

Table R1: Comparison of related multimodal works.

Ref.		[28]	[29]	[30]	[31]	[22]	Our work
Technology innovation for two modes	ECoG channel count	6	---	1 (EEG)	1 (LFP)	64 (256)	32
	ECoG coverage (mm^2)	---	---	---	---	3.1*2.8	6*8
	Calcium coverage (mm^2)	---	10*12	0.1*0.1	hemisphere	0.96*0.96	10*12
	Neuron number	---	---	18	Wide-field	136	17000
CODE enabled findings(“4D+2P”)	Definition of neuronal ensembles	---	---	ECoG-prior classification (Hypothesis-driven)	---	---	Unsupervised clustering (Data-driven)
	Distribution of neuronal ensembles	---	---	N/A (local sampling)	---	---	Cortex-wide mapping (Large-scale organizational principle)
	Difference of Burst & Supp.	---	---	PV+ interneurons modulate local pyramidal synchrony. (Local mechanism)	---	---	Population synchrony bifurcation underlies cortex-wide burst-suppression. (systems-level organization)
	Dynamic switch of two ensembles	---	---	Static (assumed fixed roles, limited neurons)	---	---	Dynamic recruitment (systematic switching, 17000 neurons across cortex)
	Propagation pathway	---	---	---	Slow Ca^{2+} wave propagation during burst maintenance at second-level timescale	---	Fast propagation during burst onset at millisecond-level timescale
	Predictability of two modes	---	---	---	---	Local high-fidelity decoding during awake (~136 neurons)	Whole-cortex prediction at different brain state (>17000 neurons)

4. Integrated Impact

Taken together, the contribution of this work is inherently **dual and inseparable**:

1. **A substantial technological advance** that overcomes long-standing barriers in multimodal neural interfaces.
2. **A set of biological discoveries** regarding the organization, dynamics, and propagation of burst–suppression that were inaccessible with previous methodologies.

We believe this tight coupling between technical innovation and biological insight—where new technology directly enables new understanding—is precisely the type of synergistic advance sought by *Nature Communications*.

Q1. Lack of fundamental innovation. While touted as a Cortex-wide Optical-electrical Dual-modal Explorer, the approach itself is really a combination of two published technologies, namely the RUSH wide-field Ca imaging system and ITO/PEDOT:PSS transparent ECoG arrays. The former was published in Nature Photonics 13, 809–816 (2019), and the latter was established by other research teams for quite a while now (see e.g., IEEE Trans Nanotechnol. 2017 Dec 20;17(4):701–704). Thus, while the work involves substantial systems neuroscience efforts, it lacks the kind of substantial innovation and quantum leap that are typically read in Nature Communications.

Response: We thank the reviewer for acknowledging the substantial systems-level effort in this work. We respectfully disagree that the CODE platform lacks fundamental innovation. While wide-field calcium imaging and transparent ECoG technologies have each been reported previously, achieving a **seamless, chronic, cortex-wide integration** of these modalities is *not* a matter of simply combining two existing approaches.

- **First, technological innovation:**

  (1) Precision integration beyond simple combination of existing methods

  While transparent ECoG arrays and wide-field calcium imaging have each been demonstrated, directly integrating them into a single chronic platform poses significant engineering challenges. Conventional soft ECoG electrodes often fracture or lose functionality when compressed under a coverslip, making long-term stable recording extremely difficult. In contrast, we designed and fabricated the CODE array directly on the coverslip using a customized MEMS microfabrication process. This required systematic optimization of material selection, process parameters, and encapsulation strategies. It is a bespoke design that eliminates the issues of misalignment, mechanical stress, and interfacial failure common when attempting to combine a separate flexible electrode array with a glass window. These steps were essential to achieve durable, chronic electrical recording without sacrificing optical clarity or spatial coverage. If the system were merely a simple combination of prior technologies, such long-term stability and high surgical success rates would not be achievable in practice. Early attempts in our lab to combine separate transparent ECoG arrays with the RUSH window failed due to chronic stability problems, such as interfacial bubbles, electrode delamination, and motion artifacts. The CODE system's integrated design specifically solves these problems, enabling the >7 weeks of stable simultaneous recording demonstrated here, which is critical for long-term studies.

  (2) Scalable, cortex-wide mapping with precise anatomical correspondence

  Our goal was to support cortex-wide, simultaneous optical–electrical recording across an unusually large cranial window. This introduces an additional challenge: ensuring each ECoG channel is precisely

positioned over anatomically defined cortical regions while maintaining a uniform, optically compatible surface. Our integrated, one-piece design ensures that electrode positions remain stable and accurately registered to the wide-field optical map. This level of alignment is difficult—if not impossible—to achieve reliably when transparent electrodes and imaging windows are assembled as separate components. Implanting a stable, large-area device that maintains perfect optical clarity and electrical contact across the entire dorsal cortex presents immense surgical challenges. We have developed and refined a surgical procedure that achieves this with high success rates, a non-trivial advancement in itself.

(3) High practicality and broad adoptability enabled by the integrated design
A major advantage of our design is its immediate usability for the neuroscience community. Researchers who already perform cranial window imaging or ECoG recordings can adopt the CODE system without increasing surgical difficulty: during surgery, the standard coverslip is simply replaced with our electrode-integrated coverslip. This preserves existing workflows while enabling simultaneous optical and electrical recordings over the entire cortical surface. Such plug-and-play deployability is not provided by previous methods and represents a meaningful step toward making multimodal, large-scale brain mapping broadly accessible.

- **Second, the potential for future applications:**

(1) Unique advantages and broad scientific potential enabled by this integrated technology
By overcoming the above engineering barriers, the seamless integration of high-density transparent ECoG with cortex-wide single-cell–resolution calcium imaging provides capabilities that no existing technology can offer. The CODE platform uniquely enables **simultaneous high-temporal-resolution electrophysiology and high-spatial-resolution, large-scale optical mapping** across virtually the entire dorsal cortex. This combination—millisecond precision, single-neuron spatial resolution, and cortex-wide coverage—has not been achieved by any prior method. This multimodal capability opens new opportunities across multiple fundamental areas of neuroscience. For example:

- **Sleep and anesthesia research:** CODE allows real-time linkage of global ECoG rhythms with distributed neuronal population dynamics across the cortex, supporting mechanistic studies of slow waves, up/down states, and state transitions.
- **Cognition and decision-making:** The platform can simultaneously track mesoscale network oscillations and single-cell activity patterns during memory, attention, or sensorimotor tasks, offering insight into how distributed circuits coordinate behavior.
- **Consciousness and brain-state transitions:** CODE provides a unique window into how global electrical signatures relate to distributed cortical activation patterns, enabling experiments that probe theories of consciousness at both macro- and micro-scales.

Beyond the applications demonstrated in our manuscript, the platform is compatible with a broad range of optical indicators. It can therefore be extended to study **the relationships between electrical activity and**

cortex-wide neurotransmitter dynamics, specific neuron types, neuromodulatory signals, metabolic states, or other chemical processes. Such multimodal measurements were previously inaccessible due to limitations in window size, optical obstruction from electrodes, or the incompatibility of chronic electrophysiology with large optical cranial windows.

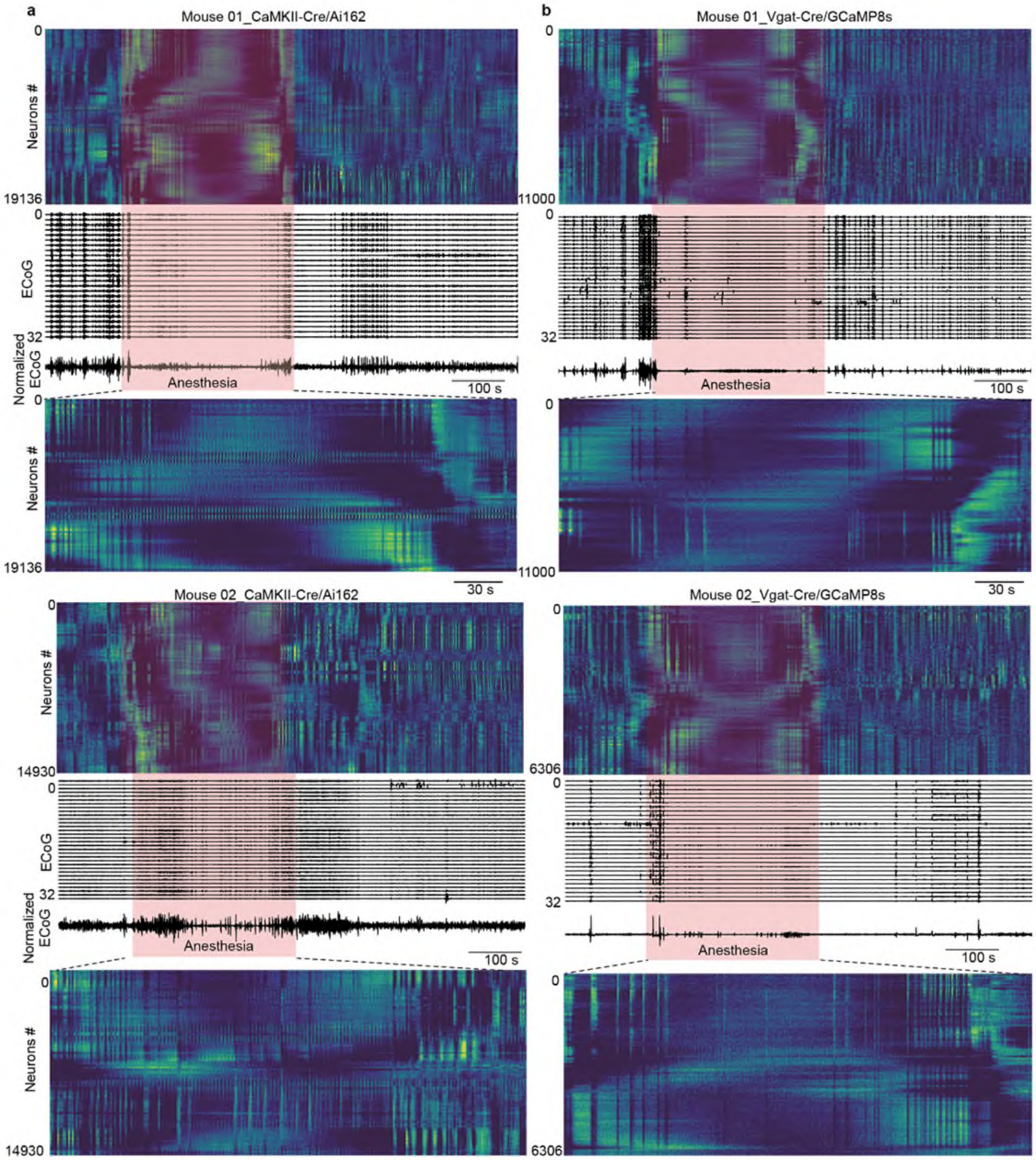

Figure R5. Normalized and sorted calcium neuronal raster plots during burst and suppression states, aligned with corresponding ECoG signals, in transgenic mice expressing GCaMP specifically in excitatory (CaMKII-Cre/Ai162d) (a) or inhibitory (Vgat-Cre/GCaMP8s) (b) neurons. Each panel includes the neuronal raster plot, the simultaneously recorded raw ECoG trace, a representative ECoG signal from the entire recording session, and enlarged views of neuronal activity during anesthesia.

We further tested the CODE system in transgenic mouse lines that selectively label excitatory neurons (CaMKII-Cre × Ai162d) and inhibitory neurons (Vgat-Cre × GCaMP8s), respectively, during anesthesia experiments. This enables us to examine cell-type–specific activity across the wide cortex. With these models, we can identify which neuronal subtypes participate in specific brain-state transitions and characterize their distinct response patterns. Moreover, the combination of cell-type specificity and cortex-wide recordings allows us to investigate how rhythm-associated neurons (e.g., neurons coupled to delta, theta, or spindle oscillations) are distributed across different cortical regions. This highlights the unique potential of CODE to dissect the contributions of defined neuronal populations to global brain dynamics. This was added in the main text (Line 257-263) and Extended data Figure 5.

(2) Enables accurate *in vivo–ex vivo* alignment for anatomical and molecular mapping

Beyond its advantages for in vivo multimodal recording, the CODE system also offers a unique benefit for in vivo–ex vivo mapping, a challenge that has limited many post hoc anatomical and molecular studies. Brain tissue typically undergoes deformation, swelling, and vascular loss during fixation and clearing (e.g., PFA perfusion and tissue clearing), which makes it extremely difficult to reliably align in vivo functional data with ex vivo histology, such as immunohistochemistry or cell-type–specific labeling.

In the CODE platform, however, the electrode sites and conductive traces embedded on the coverslip remain visible after fixation and tissue processing, effectively serving as stable, intrinsic fiduciary markers. These structural features help preserve spatial correspondence and allow us to align in vivo functional activity with ex vivo cellular or molecular readouts more accurately than previous approaches. We have already performed pilot experiments demonstrating the feasibility of this alignment strategy, and we believe this capability offers a promising direction for future applications, especially for studies aiming to link neural dynamics with genetically or molecularly defined cell types. This was also added in the main text (Line 513-522), methods session (Line 571-611) and Extended Data Figure 14.

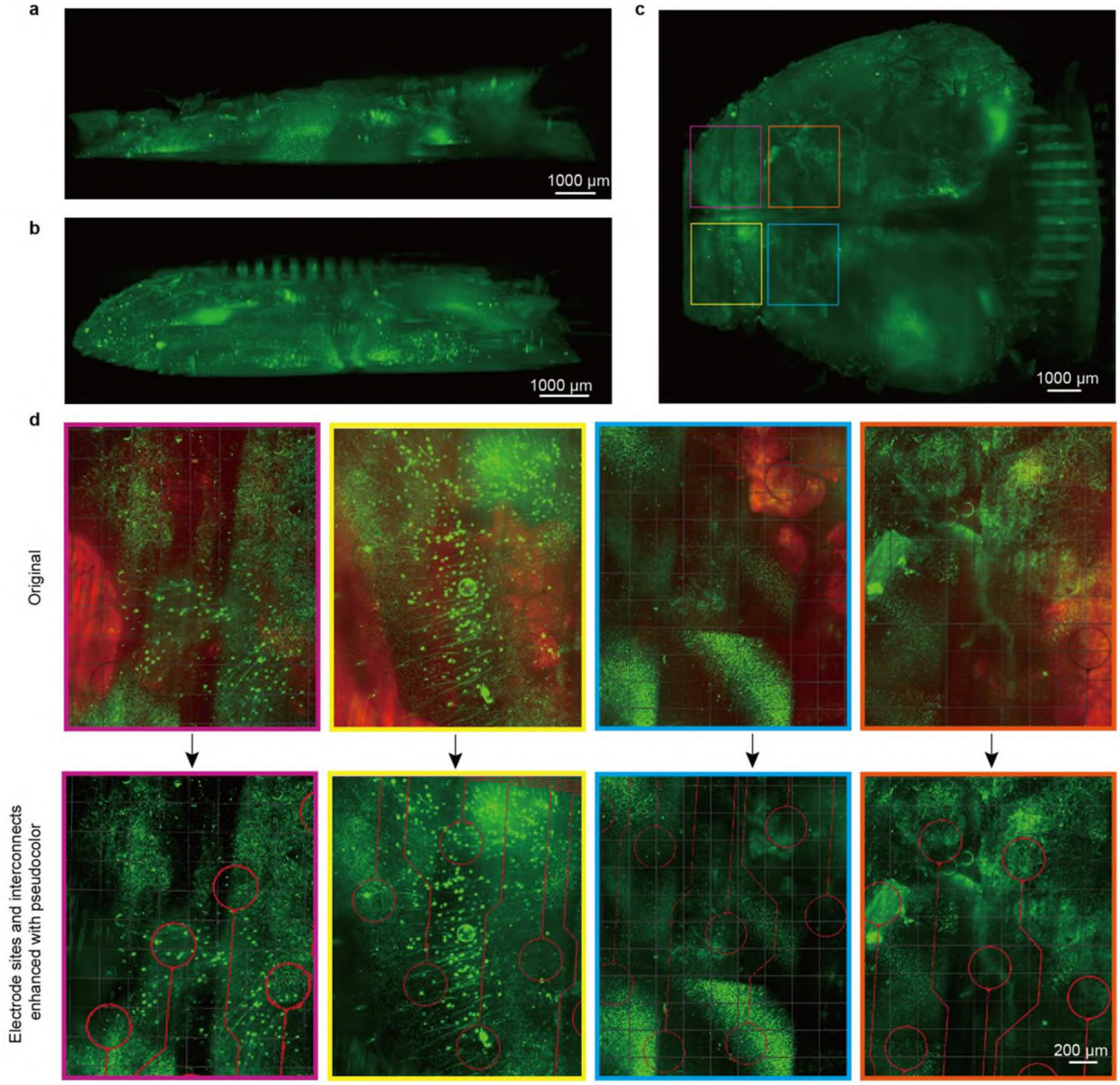

Figure R6. ECoG-guided localization of labeled neurons in Thy1-YFP mouse brain sections. a–c, Three orthogonal views of a mouse brain slice are shown from the frontal (a), left lateral (b), and dorsal (c) perspectives. d, An enlarged view of the brain regions marked in (c) with four colored boxes (purple, yellow, blue, and orange). For each region, both the original signal (top) and pseudocolor-enhanced images highlighting electrode sites and wires (bottom) are presented to illustrate the spatial relationship between the electrodes and the labeled neurons. The electrodes were instrumental in mapping signals both in vivo and in vitro.

In summary, although CODE builds upon previous conceptual components, the innovations in integration strategy, chronic stability, spatial precision, and broad usability collectively constitute a **substantial technical advance**, offering a new enabling technology with the potential to accelerate multiple domains of systems and cognitive neuroscience.

Q2. Lack of substantially novel findings. Many of the findings in neuroscience presented in this manuscript are not new.

• a. Br J Anaesth 2025 Apr;134(4):1122-1133 has reported a similar on-off pattern in neural activities under isofluorane and performed a similar classification of neurons into burst-related and suppression-related ensembles.

• b. Front Syst Neurosci. 2021 Jan 12;14:599781 has reported the spatial structure and propagation of isoflurane-induced burst suppression, with much finer spatial resolution compared to ECoG-based clustering in this manuscript.

• c. Nature Nanotechnology 19, 504–513 (2024) has reported the prediction of cellular calcium activity at depth from surface potential recordings.

• d. As a result, while this reviewer commends the grand effort and high-quality presentation, this manuscript appears to be more suitable for a more specialized journal in systems neuroscience, where the details of the findings can be fully debated and disseminated.

Response: We thank the reviewer for raising these insightful points and for citing several relevant studies. We agree that individual elements of burst–suppression dynamics have been examined previously. However, we respectfully argue that the novelty of our work lies not in isolated observations, but in the **systems-level insights that emerge uniquely from cortex-wide, single-neuron–resolved, dual-modality recordings**, which were not accessible in prior studies.

Below, we clarify how our findings extend beyond the cited literature:

(a) Br J Anaesth (2025): Neuronal ensemble classification

The cited study identified burst-related and suppression-related neurons using local two-photon imaging (Diameter is 100 µm, only one small brain regions) but was fundamentally constrained by its **very small sampling (~18 neurons)**. This limitation restricts the ability to infer generalizable principles or to characterize the full dynamics of burst–suppression.

In contrast, our work tracks **over 17,000 neurons across the entire dorsal cortex** (FOV is 1×1.2cm^2) enabling several advances that are qualitatively new:

1. Cortex-wide, unbiased ensemble mapping

We provide the first panoramic map of how burst- and suppression-related neurons are distributed across multiple cortical areas. Such large-scale organizational principles cannot be inferred from small local fields of view.

2. Discovery of dynamic ensemble switching

Crucially, we show that ensemble membership is not static.

- Burst neurons do not remain burst neurons across cycles.
- Suppression neurons are not stably suppression neurons.
- Ensemble identity changes systematically with anesthetic depth and burst–suppression duration.

This dynamic recruitment and reactivation is a new biological finding made possible only by large-scale single-neuron tracking.

3. Population-level synchrony structure

We quantify, for the first time, that burst-related neurons exhibit high functional synchrony, whereas suppression-related neurons remain asynchronous. This cortex-wide synchrony bifurcation cannot be resolved when only a few neurons are recorded.

4. Data-driven classification

Our ensemble identification arises from unsupervised clustering of large-scale calcium signals without using ECoG priors—allowing a global, data-driven view of neuronal population structure rather than relying on hand-picked neurons.

5. Methodological scalability

Traditional local-imaging approaches cannot study global propagation, multi-region coordination, or cortex-scale synchrony. The CODE system uniquely enables these analyses.

Thus, our results move far beyond simply confirming the existence of burst/suppression ensembles; they reveal their large-scale organization, dynamic transitions, and network-level interactions, fundamentally advancing the understanding of burst–suppression.

(b) Front. Syst. Neurosci. (2021): Spatial propagation

The cited work used LFP to examine spatial propagation of slow calcium waves during burst maintenance. While valuable, it differs significantly from our approach in resolution, modality, and scientific focus.

1. Millisecond-scale analysis of burst onset

We focus on the critical tens of milliseconds surrounding burst onset, a window essential for understanding state transitions. This temporal precision is not accessible with slower imaging or LFP-only approaches.

2. Consistent cortex-wide propagation patterns

We identify a stereotyped propagation trajectory across animals—from bilateral sensory regions toward medial motor cortex. This cross-animal consistency provides a mechanistic anatomical pathway that was not previously reported.

3. Simultaneous macro–micro comparison

CODE uniquely enables side-by-side comparison of macroscopic ECoG wave propagation and microscopic single-neuron activation sequences. This reveals a synchrony gradient, showing that ECoG waves become increasingly aligned with underlying calcium activity along the propagation path—a mechanistic insight inaccessible to LFP alone.

4. Single-neuron resolution

We validate propagation at the level of individual neurons, providing a direct link between local recruitment and global waveform, which was not possible with previous methods. Importantly, our study addresses a different scientific question—the fast suppression-to-burst transition—rather than slower calcium-wave propagation during burst periods. Thus, the two studies are complementary rather than overlapping.

(c) Nat. Nanotechnol. (2024): Cross-modal prediction

We thank the reviewer for highlighting this excellent technical advance. Indeed, the cited study impressively advances single-cell decoding fidelity from high-density nanoelectrode arrays within a localized cortical region. However, our work addresses a complementary and distinct scientific question. Rather than maximizing reconstruction of individual neurons in a small area, we seek to uncover system-level principles linking brain-wide population dynamics to macroscopic field potentials at different brain states involving awake, burst and suppression. This fundamental difference in objective underpins our key findings:

1. Brain State as a Central Determinant of Predictability:

Whereas the cited study demonstrates decoding performance under certain states, we place brain state itself at the center of our investigation. Crucially, we show that cross-modal predictability is strongly state-dependent (burst > suppression > awake), suggesting that macro–micro coupling reflects underlying variations in population synchrony and dynamical complexity. This mechanistic insight into state-dependent cross-scale integration extends beyond the scope of the technology-driven goals of the cited work.

2. From Local Decoding to Global Coupling at superior prediction scale:

We developed a framework relating standard-resolution ECoG (32 channels) to the activity of >17,000 neurons across wide cortex. Our focus is on how much population-level structure (captured by shared variance components) can be recovered from low-dimensional macroscopic signals—a fundamentally different challenge from point-to-point, high-density decoding. Our approach enables cross-modal prediction of population calcium dynamics at unprecedented scale—over 17,000 neurons simultaneously—using only 32-channel ECoG signals, far exceeding the scope of the cited work (136 neurons). Moreover, our predictive framework also achieves high accuracy: during burst-suppression states, the top 10 predicted SVCs explained ~60% of reliable population variance, compared to ~30% in the awake state. These results demonstrate both the scalability and quantitative robustness of our method for inferring large-scale neural dynamics from clinically relevant recordings.

(d) Suitability for *Nature Communications*

Taken together, although prior studies have explored individual aspects of burst–suppression, **none have provided a unified, cortex-wide, single-neuron–resolved view that directly links macroscopic ECoG dynamics to microscopic population activity across brain states.**

We therefore believe this work is well aligned with the scope of *Nature Communications*, as it combines:

- a substantial technological advance enabling stable, cortex-wide multimodal recording, and
- new biological insights into ensemble organization, dynamic switching, rapid propagation, and state-dependent cross-scale coupling.

Q3. none of the statistical results mentions the specific test used.

Response: We thank the reviewer for pointing this out. We apologize for the lack of explicit reporting of statistical tests in the initial submission. We have now carefully revised the Results, Methods, and figure legends to mention the detail statistical figures.

Reviewer #3

I co-reviewed this manuscript with one of the reviewers who provided the listed reports. This is part of the Nature Communications initiative to facilitate training in peer review and to provide appropriate recognition for Early Career Researchers who co-review manuscripts.

Response: We thank the reviewer for informing us of this and appreciate the contribution of both reviewers to the evaluation of our manuscript. We are grateful for the thoughtful and detailed feedback provided.

Reference:

1. Sirmpilatze, N., et al., *Spatial signatures of anesthesia-induced burst-suppression differ between primates and rodents.* Elife, 2022. **11**.
2. Donald L. Clark, B.S.R., *Neurophysiologic effects of general anesthetics.* Anesthesiology, 1973. **35**: p. 17.
3. Krahe, R. and F. Gabbiani, *Burst firing in sensory systems.* Nat Rev Neurosci, 2004. **5**(1): p. 13-23.
4. Lewis, L.D., et al., *Local cortical dynamics of burst suppression in the anaesthetized brain.* Brain, 2013. **136**(Pt 9): p. 2727-37.
5. Muller, L., et al., *Cortical travelling waves: mechanisms and computational principles.* Nat Rev Neurosci, 2018. **19**(5): p. 255-268.
6. Bharioke, A., et al., *General anesthesia globally synchronizes activity selectively in layer 5 cortical pyramidal neurons.* Neuron, 2022. **110**(12): p. 2024-2040.e10.
7. Park, D.-W., et al., *Graphene-based carbon-layered electrode array technology for neural imaging and optogenetic applications.* Nature Communications, 2014. **5**(1).
8. Thunemann, M., et al., *Deep 2-photon imaging and artifact-free optogenetics through transparent graphene microelectrode arrays.* Nature Communications, 2018. **9**(1).
9. Park, D.-W., et al., *Fabrication and utility of a transparent graphene neural electrode array for electrophysiology, in vivo imaging, and optogenetics.* Nature Protocols, 2016. **11**(11): p. 2201-2222.
10. Driscoll, N., et al., *Multimodal in vivo recording using transparent graphene microelectrodes illuminates spatiotemporal seizure dynamics at the microscale.* Communications Biology, 2021. **4**(1).
11. *Transparent arrays of bilayer-nanomesh microelectrodes for simultaneous electrophysiology and two-photon imaging in the brain.* Science Advances, 2018-09-07. **4**(9): p. eaat0626.
12. Seo, J.W., et al., *Artifact-Free 2D Mapping of Neural Activity In Vivo through Transparent Gold Nanonetwork Array.* Advanced Functional Materials, 2020. **30**(34).
13. Zhang, J., et al., *Stretchable Transparent Electrode Arrays for Simultaneous Electrical and Optical Interrogation of Neural Circuits in Vivo.* Nano Letters, 2018. **18**(5): p. 2903-2911.
14. Wang, X., et al., *Subdural neural interfaces for long-term electrical recording, optical microscopy and magnetic resonance imaging.* Biomaterials, 2022. **281**.
15. Nishimura, A., et al., *Totally transparent hydrogel-based subdural electrode with patterned salt bridge.* Biomedical Microdevices, 2020. **22**(3).
16. Dijk, G., et al., *Fabrication and in vivo 2-photon microscopy validation of transparent PEDOT:PSS microelectrode arrays.* Microsystems & Nanoengineering, 2022. **8**(1).
17. Cho, Y.U., et al., *Ultra-Low Cost, Facile Fabrication of Transparent Neural Electrode Array for Electrocorticography with Photoelectric Artifact-Free Optogenetics.* Advanced Functional Materials, 2021. **32**(10).
18. Donaldson, P.D., et al., *Polymer Skulls With Integrated Transparent Electrode Arrays for Cortex-Wide Opto-Electrophysiological Recordings.* Advanced Healthcare Materials, 2022. **11**(18).

19. Liu, X., et al., *E-Cannula reveals anatomical diversity in sharp-wave ripples as a driver for the recruitment of distinct hippocampal assemblies.* Cell Reports, 2022. **41**(1).

20. Jia, H., et al., *In vivo two-photon imaging of sensory-evoked dendritic calcium signals in cortical neurons.* Nature Protocols, 2010. **6**(1): p. 28-35.

21. Ghanbari, L., et al., *Cortex-wide neural interfacing via transparent polymer skulls.* Nature Communications, 2019. **10**(1).

22. Ramezani, M., et al., *High-density transparent graphene arrays for predicting cellular calcium activity at depth from surface potential recordings.* Nat Nanotechnol, 2024. **19**(4): p. 504-513.

23. Rupprecht, P., et al., *A database and deep learning toolbox for noise-optimized, generalized spike inference from calcium imaging.* Nature Neuroscience, 2021. **24**(9): p. 1324-1337.

24. Theis, L., et al., *Benchmarking Spike Rate Inference in Population Calcium Imaging.* Neuron, 2016. **90**(3): p. 471-482.

25. Ward, M.P., et al., *Toward a comparison of microelectrodes for acute and chronic recordings.* Brain Res, 2009. **1282**: p. 183-200.

26. Zhao, S., et al., *Full activation pattern mapping by simultaneous deep brain stimulation and fMRI with graphene fiber electrodes.* Nature Communications, 2020. **11**(1).

27. Zhang, F., et al., *Multimodal Electrocorticogram Active Electrode Array Based on Zinc Oxide-Thin Film Transistors.* Adv Sci (Weinh), 2023. **10**(2): p. e2204467.

28. Yang, W., et al., *Characteristics of Transparent, PEDOT:PSS Coated Indium-Tin-Oxide (ITO) Microelectrodes.* IEEE Trans Nanotechnol, 2018. **17**(4): p. 701-704.

29. Fan, J., et al., *Video-rate imaging of biological dynamics at centimetre scale and micrometre resolution.* Nature Photonics, 2019. **13**(11): p. 809-816.

30. Yin, M., et al., *Synchronicity of pyramidal neurones in the neocortex dominates isoflurane-induced burst suppression in mice.* Br J Anaesth, 2025. **134**(4): p. 1122-1133.

31. Ming, Q., et al., *Isoflurane-Induced Burst Suppression Is a Thalamus-Modulated, Focal-Onset Rhythm With Persistent Local Asynchrony and Variable Propagation Patterns in Rats.* Front Syst Neurosci, 2020. **14**: p. 599781.

Manuscript: NCOMMS-25-58362-A

Title: Dynamic neuronal ensembles encode burst-suppression revealed by cortex-wide optical-electrical interfaces

Jan 30, 2026

Point by point responses to reviewer comments:

Reviewer #1:

Comments: The authors have addressed all my comments and revised unnecessarily strong claims that would need more experimental support. I do not have further comments. The manuscript can be accepted.

Response: We sincerely thank the reviewer for the thorough and constructive evaluation of our manuscript. The reviewer's comments played an important role in strengthening the manuscript and helped us further improve the clarity of the text, refine several key arguments, and ensure that the conclusions are firmly grounded in the presented data. We believe that these revisions guided by the reviewer's suggestions have meaningfully enhanced the rigor and overall quality of the study.

Reviewer #2

Q1: This reviewer appreciates the extensive effort that the authors have taken to address the previous comments. In the spirit that science thrives on criticism, this reviewer has the following re-rebuttal:

"prior transparent electrode demonstrations have been limited by interfacial instability, optical degradation, or restricted coverage and longevity." This reviewer disagrees. Interfacial instability or optical degradation are not major issues from previous transparent electrodes. Previous demonstrations may have specific designs for their corresponding studies, but are not fundamentally restricted in coverage. Lastly, longevity is rarely an issue for animal ECoG.

Response: We thank the reviewer for this thoughtful assessment and for highlighting limitations in our previous wording. We agree that our earlier statement overstated the generality of issues such as interfacial instability, optical degradation, or limited coverage/longevity in prior transparent electrode demonstrations. We appreciate the reviewer for pointing this out.

Our intention was not to imply that these limitations are intrinsic or general shortcomings of transparent electrodes. Rather, our point was more specific and context-dependent. Many prior transparent electrode

implementations are thoughtfully designed and optimized for particular experimental goals, and their architectures therefore reflect deliberate trade-offs among material choice, mechanical configuration, optical access, spatial coverage, and experimental duration. As a result, these approaches are often highly effective within their intended applications.

In the present work, our experimental goals imposed a specific combination of constraints, including large contiguous cortical coverage, high optical quality compatible with wide-field single-cell imaging, and mechanical robustness suitable for large-aperture imaging geometries. Not all transparent electrode designs are expected to simultaneously satisfy all of these requirements, nor should they, as different studies naturally prioritize different performance dimensions. Importantly, this does not imply that prior approaches are fundamentally unable to meet these requirements; rather, they were not designed with this particular combination of constraints in mind.

We acknowledge that our earlier discussion did not sufficiently distinguish between approaches that were *not designed to address* certain requirements and those that are *unable to address* them. This distinction was not made clearly enough, and we have now revised the manuscript to correct this overgeneralization.

In response to the reviewer's comment and the editor's guidance, we have carefully revised the text throughout the manuscript to tone down broad claims regarding technological innovation, remove overgeneralized statements about prior transparent electrode studies, and more precisely frame the electrode design as an enabling component tailored to the specific biological questions addressed here. At the same time, we have strengthened the presentation of the biological results, emphasizing that the primary contribution of this work lies in the multimodal, cortex-wide characterization of burst–suppression dynamics, rather than in proposing a fundamentally new electrode technology.

Q2. "Conventional soft ECoG electrodes often fracture or lose functionality when compressed under a coverslip" This reviewer disagrees. How the brain is closed matters indeed, but flexible electrodes should not easily fracture under a coverslip that is flat.

Response: We thank the reviewer for this clarification and agree that, under ideal conditions, well-designed flexible ECoG electrodes should not fracture simply as a result of compression by a flat coverslip. Our earlier wording in the previous response letter was not intended to imply that mechanical failure is inevitable, nor to suggest intrinsic deficiencies in existing flexible ECoG designs.

Our concern instead relates to practical considerations encountered in wide-area optical imaging experiments. In such settings, electrode arrays are deployed over extended cortical areas and may experience non-uniform mechanical stress or strain, including curvature mismatch, wrinkling, or local buckling between the electrode and the cortical surface. These effects can be exacerbated by pressure applied during cranial window sealing, surgical mounting, or adjustment. Under such conditions, certain electrode architectures may exhibit localized

delamination, interconnect deformation, increased impedance, or partial loss of functionality, even in the absence of macroscopic fracture.

We appreciate the reviewer's emphasis on this point. We have re-checked the entire manuscript and confirm that it does not contain statements implying that flexible ECoG electrodes may fracture under flat-coverslip compression.

Q3. This manuscript indeed conducted much wider field of view imaging than Br J Anaesth 2025 Apr;134(4):1122-1133, and achieved faster imaging than Front Syst Neurosci. 2021 Jan 12;14:599781. However, these are all enabled by the RUSH system, which has been published and has little to do with multimodality. At a minimum, the results and figures should focus on the cortex-wide maps and ms-scale dynamics.

Response: We fully agree with the reviewer that the wide field of view and fast imaging performance reported in this study are enabled by the previously published RUSH system, and we appreciate the reviewer for emphasizing this important distinction. The goal of the present manuscript is not to re-establish or extend the imaging capabilities of RUSH, but to demonstrate that transparent ECoG recording can be integrated without compromising these established imaging strengths, and to apply this integrated optical–electrical framework to a specific biological question.

Accordingly, the novelty of this work lies in the biological insights enabled by simultaneous multimodal measurements, rather than in imaging performance per se. Our focus is on how cortex-wide spatial organization and millisecond-scale electrophysiological dynamics jointly evolve across burst and suppression states, and how these macro–micro dynamics can be captured only when optical and electrical recordings are acquired concurrently.

In line with the reviewer's suggestion, we have refined the presentation to further emphasize cortex-wide maps and millisecond-scale dynamics as the core results of the study. Specifically, we adjusted several key figures (e.g., Fig. 1e, Fig. 4b) to more clearly highlight synchronized, cortex-wide activity patterns across multiple regions, and to better foreground the temporal dynamics at the millisecond scale that emerge from simultaneous optical–electrical recordings. Additional examples illustrating these features have been included in the Supplementary Figures.

We thank the reviewer for this constructive guidance, which helped sharpen the focus of the manuscript and strengthen the presentation of its biological contributions.

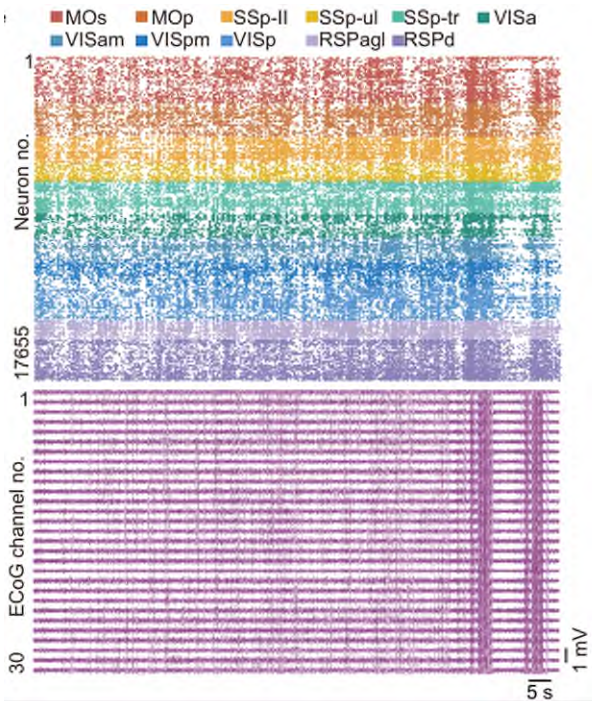

Figure R1. Simutanously recording of cortex-wide maps and ms-scale dynamics. Representative traces of simultaneously recorded calcium fluorescence (top) involving MOs, Mop, SSp-ll, SSp-ul, VISa, VISam, VISpm, VISp, RSPagl, RSPd regions and electrophysiological signals (bottom) from the 32-channel ECoG array, illustrating temporal synchronization between optical and electrical modalities.

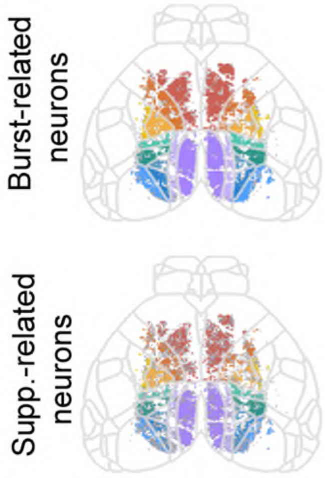

Figure R2. Two kinds of neurons distributed across wide cortex that respond to burst events, suppression events, and awake stages. Cortex map across multiple regions involving MOs, Mop, SSp-ll, SSp-ul, VISa, VISam, VISpm, VISp, RSPagl, RSPd spread out on both sides.

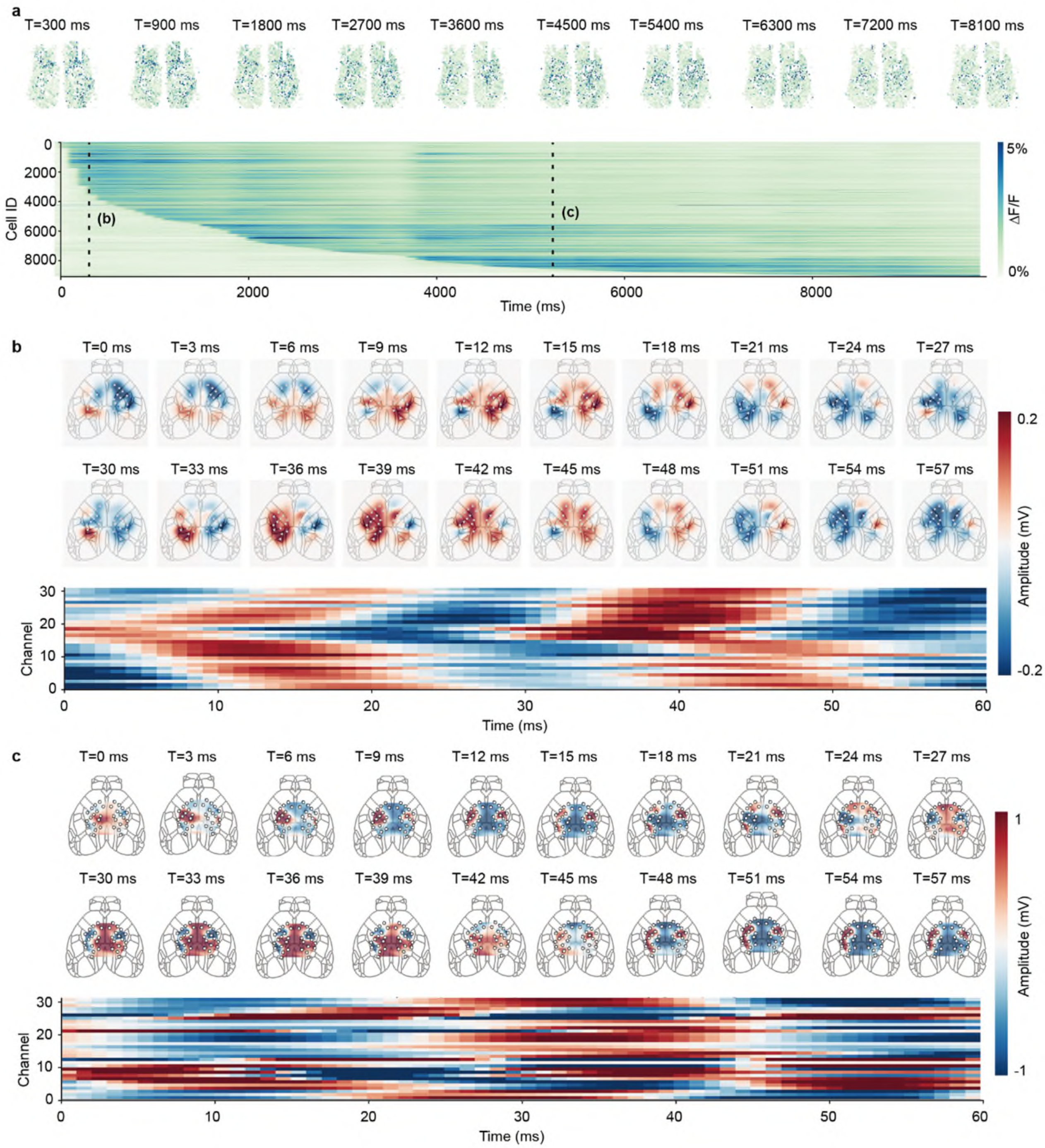

Figure R3. **Characterization of rapidly propagating spatiotemporal patterns and neuronal recruitment during a burst event. a,** Single-cell–level cortical dynamics characterized using calcium imaging. Neurons are arranged according to their activation onset time (bottom). **b–c,** Dynamics of ECoG signals within a 60-ms window corresponding to the period marked in **a**, shown at burst onset (**b**) and during the burst (**c**). The upper panels visualize 20 snapshots at the latencies indicated above.

Q4. That being said, this reviewer can appreciate the strong utility demonstrations from the integrated system, multimodal analytics and their potential broad appeal to the systems neuroscience research community.
In summary, this reviewer agrees with reviewer 1 that for this manuscript, the "novelty is in the application of multimodal approach into a specific neuroscience question related to burst-suppression." Therefore, the authors should at least downplay many big innovation claims on the technology side, revise many seemingly inaccurate statements, and better report their biological data/findings.

Response: We sincerely thank the reviewer for recognizing the utility of the integrated system, the multimodal analytics, and their potential broad appeal to the systems neuroscience community. We also appreciate the reviewer's clear articulation of where the core novelty of this work lies. We fully agree that, for this manuscript, the primary novelty is not the invention of a fundamentally new imaging or electrode technology. Rather, the central contribution is the application of an integrated multimodal approach to address a specific and important neuroscience question related to burst–suppression, while also demonstrating how such integrated measurements can enable the investigation of a broader range of biological questions that require simultaneous access to cortex-wide electrical dynamics and large-scale cellular activity.

We acknowledge that some of our original wording overstated the degree of technological innovation. In direct response to the reviewer's comments, we have comprehensively revised the manuscript to reposition it as a question-driven study. Specifically, throughout the revised manuscript, both the RUSH imaging system and the transparent ECoG interface are consistently framed as enabling measurement components rather than as primary technological innovations. Correspondingly, we have refocused the narrative on how the integration of these established tools supports biological discovery. Throughout the revision, we emphasized biological interpretation and data analysis, while carefully removing or revising statements that could be interpreted as overgeneralized or inaccurate with respect to prior transparent electrode and soft ECoG studies.

Consistent with this refocusing, we revised the **Abstract**, **Introduction**, **Results** and **Discussion** sections to emphasize biological findings derived from the combined optical–electrical measurements, including cortex-wide spatial organization, millisecond-scale dynamics, neuronal ensemble recruitment, propagation patterns, and state-dependent optical–electrical relationships. Several figures were replotted or reformatted (e.g., Fig. 1e, Fig. 4b, and related Supplementary Figures) to more clearly highlight cortex-wide maps and ms-scale dynamics, and additional Supplementary Figures were included to strengthen the presentation of these biological insights.

All revisions are highlighted in yellow in the revised manuscript for the reviewer's convenience. Below, we list several representative examples of major textual changes.

- In **Abstract** section:

  The original wording "*…yet its neuronal underpinnings remain elusive due to the lack of recording technologies with both high spatiotemporal resolution and large-scale coverage. Here, we present a Cortex-wide Optical-electrical Dual-modal Explorer (CODE) system enabling simultaneous mesoscale calcium imaging of over 17,000 neurons and 32-channel electrocorticography (ECoG) recordings.*" was revised to "*…yet its neuronal organization across the cortex remains incompletely understood. Here, we present an integrated Cortex-wide Optical-electrical Dual-modal Explorer (CODE) system to examine cortex-wide neuronal dynamics during burst-suppression under*

isoflurane anesthesia." to remove emphasis on technological novelty and to foreground the biological question and cortex-wide neuronal organization.

- In **Introduction** section: Several statements emphasizing technological limitations of prior approaches were removed or revised. For example:

(Line 72–78) "*While EEG or ECoG recordings offer millisecond-level temporal precision, their poor spatial resolution precludes insights into single-cell behaviors. On the other hand, two-photon microscopic calcium imaging has provided single-neuron resolution within sub-millimeter field-of-view (FOV), revealing synchronized pyramidal neuron activity during anesthesia. Yet, these optical approaches suffer from limited spatial coverage and frame rates insufficient to resolve rapid brain-state transitions across extensive cortical regions.*" have been removed to avoid statements regarding technological limitations of prior studies.

(Line 74–79) "*ECoG fabrication increasingly relies on transparent electrode technologies—including graphene, metal nanoparticles, carbon nanotubes, hydrogel, and PEDOT-only systems, which make it possible to combine calcium imaging with electrophysiology. Attempts to integrate these modalities, such as pairing two-photon calcium imaging with transparent ECoG arrays or with whole-cell patch-clamp recordings have offered improved spatial resolution but remain constrained to small field of view and relatively sparse neuronal populations, or employing methods such as wide-field imaging combined with ECoG to monitor large cortical areas, low spatial resolutions, limited the identification of specific functional neuron types.*" have been changed to "*In recent years there have been increasing efforts to integrate optical imaging with surface electrophysiology using transparent electrodes based on graphene, metal nanoparticles, carbon nanotubes, hydrogel, and PEDOT-based systems. These studies have enabled multimodal measurements and have been optimized for specific experimental goals, such as pairing two-photon cellular-resolution calcium imaging with transparent ECoG arrays or wide-field monitoring with limited cellular specificity.*" to emphasize that prior multimodal approaches were optimized for specific experimental goals, rather than being fundamentally constrained.

(Line 99–101) "*Unlike traditional methods that offer either high spatial resolution with limited field-of-view (e.g., two-photon imaging) or broad cortical coverage without single-cell access (e.g., electrophysiology), the CODE system bridges these scales by simultaneously capturing population-level and single-neuron dynamics across the entire dorsal cortex.*" have been changed to "*Using this integrated multimodal measurement strategy, we investigated the neuronal organization and spatiotemporal dynamics of anesthesia-induced burst-suppression at cortex-wide scale." to remove the statement describing previous methods*" to focus instead on the biological investigation enabled by an integrated measurement strategy.

- In **Results** section: In the first two subsections describing the approach and the experimental configuration, language emphasizing technological novelty was systematically downplayed. The focus was shifted toward characterization and application of the measurement configuration to address specific biological questions. For example:

(Line 128–130) "*However, combining optical and electrophysiological modalities presents significant challenges, including imaging obstruction by opaque electrodes and mechanical instability of cranial implants over chronic timescales.*" have been removed.

(Line 141–142) "*The completed ECoG interface exhibits excellent optical clarity. When mounted over the mouse cortex following skull removal, cortical vasculature remains clearly visible (Extended Data Fig.1c,d).*" have been changed to "*Optical clarity was confirmed after implantation, with cortical vasculature remaining clearly visible (Extended Data Fig. 1c,d).*" to ensure neutral and descriptive tone.

(Line 344–352) "*Building on our characterization of cortex-wide electrical and calcium dynamics during burst–suppression, we further examined millisecond-scale spatiotemporal patterns in ECoG signals aligned to cortex-wide maps. During the suppression-to-burst transition, ECoG activity exhibited temporally ordered spatial patterns across channels (Extended Data Fig. 12a,b); in contrast, same analysis applied to ECoG signals during the sustained burst phase revealed more spatially heterogeneous patterns (Extended Data Fig. 12c). These observations indicate that the spatiotemporal organization of ECoG activity differs across phases of burst–suppression, with more structured*

patterns emerging transiently around state transitions." was added to highlight millisecond-scale spatiotemporal ECoG dynamics during suppression-to-burst transitions to emphasize biological insights uniquely revealed by these combined measurements.

We also carefully reviewed the entire **Results** sections to ensure that there are no overstatements of our technology. All corresponding revisions are highlighted in yellow throughout the main manuscript for the reviewer's convenience.

- In **Discussion** section: We likewise revised the entire section to align with these requirements. For example:
- (Line 479–480) "*Our innovative approach—enabling simultaneous recording of large-scale neuronal activity and ECoG across wide cortical areas—allowed us…*" have been changed to "*By combining the recording of large population calcium activity and ECoG across wide cortical areas, CODE allowed us…*" to tone down the degree of novelty on the technology side.

  (Line 508–513) "*This expands the potential of the CODE platform, which uniquely combines high-temporal-resolution electrophysiology with high-spatial-resolution optical mapping across nearly the entire dorsal cortex. This enables the study of electrical activity in relation to cortex-wide neurochemical, metabolic, and modulatory processes, and contributes to research on sleep, anesthesia, cognition, decision-making, consciousness, and brain-state transitions.*" have been removed to leave out forward-looking statements regarding technology and future electrode designs, and to maintain focus on the biological findings presented in this study enabled by dual modal interfaces.

Overall, these revisions reposition the manuscript as a problem-driven study, in which integrated multimodal measurements are uniquely used as analytical tools to exploit its broad spatiotemporal resolution on the study of burst–suppression. We sincerely thank the reviewer for these constructive comments, which played an important role in sharpening the focus, rigor, and clarity of the manuscript.

Reviewer #3

I co-reviewed this manuscript with one of the reviewers who provided the listed reports. This is part of the Nature Communications initiative to facilitate training in peer review and to provide appropriate recognition for Early Career Researchers who co-review manuscripts.

Response: We thank the reviewer for informing us of this and appreciate the contribution of both reviewers to the evaluation of our manuscript. We are grateful for the thoughtful and detailed feedback provided.
